# ﻿*Dicranota* Zetterstedt, 1838 crane flies (Diptera, Pediciidae) of Korea

**DOI:** 10.3897/zookeys.1253.146576

**Published:** 2025-09-23

**Authors:** Sigitas Podenas, Jin Whoa Yum, Neung-Ho Ahn, Soen Yi Kim, Jisoo Kim, Virginija Podeniene

**Affiliations:** 1 State Scientific Research Institute Nature Research Centre, Akademijos str. 2, LT-08412 Vilnius, Lithuania Life Sciences Centre of Vilnius University Vilnius Lithuania; 2 Life Sciences Centre of Vilnius University, Sauletekio str. 7, LT-10257 Vilnius, Lithuania State Scientific Research Institute Nature Research Centre Vilnius Lithuania; 3 Biodiversity Research Department, Species Diversity Research Division, National Institute of Biological Resources, Incheon 22689, Republic of Korea Species Diversity Research Division, National Institute of Biological Resources Incheon Republic of Korea; 4 Biological Specimen Conservation Division, Nakdonggang National Institute of Biological Resources, Sangju, Gyeongsangbuk-do 37242, Republic of Korea Biological Specimen Conservation Division, Nakdonggang National Institute of Biological Resources Sangju Republic of Korea

**Keywords:** East Palaearctic, habitat, key, Pediciinae, taxonomy

## Abstract

Pediciinae crane flies belonging to the genus *Dicranota* Zetterstedt, 1838 of the Korean Peninsula were studied beginning in 1933, but only seven species have been recorded from North Korea so far; the genus was unknown from South Korea. Seventeen species were found during our studies on the Peninsula, three of them described as new: D. (Eudicranota) distincta Podenas, **sp. nov.**, D. (Rhaphidolabis) seoi Podenas, **sp. nov.**, D. (Rhaphidolabis) yeongokia Podenas, **sp. nov.** Ten new species are added to the Korean species list, five of them new for North Korea with total number reaching twelve, and eleven species new for South Korea. Habitat, elevation range, and seasonality data is presented for each species. Images of taxonomically important morphological characters, distribution maps, and an identification key for all Korean species of the genus *Dicranota* are presented.

## ﻿Introduction

Research of Korean crane flies is on-going since 2012, when it was started together with researchers from the National Institute of Biological Resources. The aim of the study is to register, redescribe, illustrate, and prepare keys for all Korean species. We decided that redescriptions, illustrations, and keys should be based on Korean specimens, because variation of morphological features was noticed in crane flies belonging to same species but collected in different localities across the Palaearctic. Forested mountains cover more than two-thirds of South Korea‘s territory. Even though they are not very high, most of them are granitic with many beautiful small and fast running streams and springs. Larvae of Pediciidae (Pediciinae) crane flies belonging to the genus *Dicranota* Zetterstedt, 1838 develop on the bottom of these aquatic habitats. They prefer clean cold water and are predatory feeding on different small invertebrates. Adults fly nearby and hide in herbaceous vegetation during the day, becoming more active in the dusk, and some species are attracted to light. They are often met flying together with other Pediciidae crane flies, especially *Tricyphona* Zetterstedt, 1837 and could be separated from them based on wing venation, especially the short radial sector. The first *Dicranota* specimens from the Korean peninsula included in this publication were collected more than 90 years ago, while the oldest specimens from neighbouring countries, especially Japan, were obtained more than 100 years ago. Despite intense work on Korean crane flies for many years, only seven *Dicranota* species had been recorded from North Korea, and the genus was unknown from South Korea (Oosterbroek 2025).

## ﻿Materials and methods

Despite the many museum collections that were checked, *Dicranota* crane flies from the Korean Peninsula were found only at the National Institute of Biological Resources (**NIBR**), Incheon, South Korea; the Korea University Entomological Museum (**KUEM**), Seoul, South Korea; the Snow Entomological Museum, University of Kansas, Lawrence, KS, USA (**SMEK**); the National Museum of Natural History, Smithsonian Institution, Washington, DC, USA (**USNM**); the Hungarian Natural History Museum (**HNHM**) in Budapest, Hungary; and at the Nature Research Centre (**NRC**), Vilnius, Lithuania (Table 1). Comparative material from Mongolia was obtained from The Academy of Natural Sciences of Drexel University, Philadelphia, PA, USA (**ANSP**).

**Table 1.** Collecting sites in Korea.

Locality	Year	Latitude, Longitude*	Collector	Method	Depository
N. Korea, Mt. Kongo San (Mt. Geumgangsan) [Kongô-san, Kôgen-dô]	1933	38.65713°N, 128.10167°E	G. Machida	Net	USNM
N. Korea, Chonsani Paiktusan (Yanggang-do, Dachongdan-gun)	1937	41.99360°N, 128.75250°E	A.M. Yankovsky	Net	USNM
N. Korea, Ompo (now called Onbo, Hamgyeongbuk-do, Gyeongsung-gun)	1937 1938	41.51357°N, 129.57812°E	A. M. Yankovsky	Net	USNM
N. Korea, Seren Mountains (Hamgyeongbuk-do, Gyeongsung-gun)	1937 1938	41.68730°N, 129.30918°E	A. M. Yankovsky	Net	USNM
N. Korea, Kankyo Nando, Puksu Pyaksan (now Yanggang-do, Pungseo-gun, Mt. Buksubaeksan)	1939	40.69985°N, 127.71601°E	A. M. Yankovsky	Net	USNM
N. Korea, Pontani Paiktusan (Mt. Baekdusan)	1940	42.00670°N, 128.10650°E	A. M. Yankovsky	Net	USNM
N. Korea, Prov. South Phenan (Pyongyang), Bong-ha ri	1982	39.00777°N, 125.69404°E	ForrÓ, Ronkay	Net	HNHM
S. Korea, Kwangju [Gwangju]	1946	35.15641°N, 126.83745°E	S. Kramer	Net	USNM
S. Korea, #3, 7 miles W of Chungju	1954	36.97844°N, 127.80099°E	G. W. Byers	Net	USNM
S. Korea, #8, #9, #28, #39 Central National Forest, 18 miles NE Seoul	1954	37.74813°N, 127.29364°E	G. W. Byers	Net	SMEK, USNM
S. Korea, #12, Hwy. #20, 8 mi. SW Kangnung	1954	37.70000°N, 128.78333°E	G. W. Byers	Net	SMEK
S. Korea, #37, #38 Hill 1468, 16 mi. NW Chunchon	1954	38.00000°N, 127.50000°E	G. W. Byers	Net	SMEK
S. Korea, Jeollabuk-do, Muju-gun, Seolcheon-myeon, Jangdeok-ri, Gucheondong	1972	35.85996°N, 127.76578°E	C.-H. Kim	Net	KUEM
S. Korea, Gangwon-do, Pyeongchang-gun, Daegwallyeong-myeon, Yongsan-ri, Mt. Balwangsan	2008	37.61458°N, 128.67147°E	J. D. Yeo et al.	Malaise trap	NIBR
S. Korea, Gangwon-do, Gangneung, Yeongok-myeon, Samsan-ri, Odaesan National Park	2012	37.81161°N, 128.70116°E	S. Podenas	Net	NIBR
S. Korea, Gangwon-do, Pyeongchang-gun, Jinbu-myeon, Dongsan-ri, Odaesan National Park	2012 2015	37.73920°N, 128.59398°E; 37.73767°N, 128.59166°E	S. Podenas	Net	NIBR
S. Korea, Gyeongsangnam-do province, Samjeong village	2012	35.30246°N, 127.63439°E	S. Podenas	Net	NIBR
S. Korea, Jeollanam-do, Gurye-gun, Toji-myeon, Naedong-ri	2012	35.26137°N, 127.60302°E	S. Podenas	Net	NIBR
S. Korea, Jeollanam-do, Gurye-gun, Toji-myeon, Naeseo-ri, Jirisan National Park, Piagol valley	2012 2013 2015 2016 2019	35.26586°N, 127.58090°E; 35.26580°N, 127.58128°E; 35.27177°N, 127.57146°E; 35.28589°N, 127.55605°E; 35.26590°N, 127.58096°E; 35.27448°N, 127.56378°E; 35.27123°N, 127.57133°E; 35.27333°N, 127.56924°E	S. Podenas, V. Podeniene	Net, at light	NIBR
S. Korea, Jeollanam-do, Gurve, Masan-myeon, Hwangjeon-ri	2013	35.24366°N, 127.48964°E	S. Podenas	Net	NIBR
S. Korea, Jeollabuk-do, Namwon, Sannae-myeon, Deokdong-ri	2013	35.33692°N, 127.53230°E	S. Podenas	Net	NIBR
S. Korea, Gyeongsangnam-do, Hamyang, Macheon-myeon, Samjeong-ri	2013	35.35880°N, 127.63672°E; 35.34243°N, 127.64102°E	S. Podenas	Net	NIBR
S. Korea, Jeollabuk-do, Namwon, Jucheon-myeon, Gogi-ri	2013	35.38131°N, 127.48412°E	S. Podenas, H. Byun	Net	NIBR
S, Korea, Gyeongsangnam-do, Sancheong, Sicheon-myeon, Jungsan-ri	2013	35.30996°N, 127.75163°E	S. Podenas	Net	NIBR
S. Korea, Jeollabuk-do, Namwon, Unbong-eup, Hwasu-ri	2013	35.45098°N, 127.57596°E; 35.45345°N, 127.57759°E	S. Podenas, H. Byun	Net	NIBR
S. Korea, Gyeongsangnam-do, Hadong-gun, Hwagae-myeon, Beomwang-ri	2013	35.27655°N, 127.61796°E	S. Podenas	Net	NIBR
S. Korea, Gyeonggi-do, Gapyeong-gun, Buk-myeon, Hwaak-ri	2014	37.98402°N, 127.52676°E	S. Podenas, S. Kim	Net	NIBR
S. Korea, Gangwon-do, Goseong-gun, Ganseong-eup, Jinbu-ri	2015	38.26678°N, 128.35706°E	S. Kim, S. Podenas	Net	NIBR
S. Korea, Gangwon-do, Gapyeong-gun, Buk-myeon, Jeokmok-ri	2015	37.07312°N, 129.30764°E	Y. Bae	Net	KUEM
S. Korea, Gangwon-do, Inje-gun, Buk-myeon, Hangye-ri, Jayang 3 gyo (bridge), Seoraksan National Park	2015	38.10415°N, 128.37973°E	S. Kim, S. Podenas	Net	NIBR
S. Korea, Gyeongsangbuk-do, Gyeongju, Jinhyeon-dong, Tohamsan (Mt.)	2016	35.78755°N, 129.34274°E	H. M. Baek, S. Podenas	Net	NIBR
S. Korea, Gyeongsangbuk-do, Gyeongju, Yangbuk-myeon, Janghang-ri	2016	35.76236°N, 129.36407°E	H. Baek, S. Podenas	Net	NIBR
S. Korea, Gyeongsangbuk-do, Gyeongju-si, Jinhyeong-dong, Tohamsan (Mt.)	2016	35.78706–35.78947°N, 129.34211–129.34700°E	S. Podenas, H.-M. Baek	Net	NIBR
S. Korea, Gyeonggi-do, Gunpo-si, Suri-dong	2017	37.35022°N, 126.91527°E; 37.35058°N, 126.91558°E	S. Podenas, V. Podeniene	Net, at light	NIBR
S. Korea, Gyeonggi-do, Pocheon-si, Yeongjung-myeon, Yeongpyeong-ri, MPRC	2017 2019	38.03644°N, 127.23226°E; 38.03644°N, 127.23226°E	T. A. Klein, H.-C. Kim	New Jersey trap	NIBR
S. Korea, Gyeonggi-do, Yangpyeong, Cheongun-myeon, Dowon-ri	2017	37.54507°N, 127.79483°E	S. Podenas	At light	NIBR
S. Korea, Gangwon-do, Chuncheon-si, Dongsan-myeon, Kangwon National University Experimental Forest	2018	37.77909°N, 127.81580°E	S. Podenas	At light, net	NIBR
S. Korea, Gangwon-do, Chuncheon-si, Namsan-myeon, Gongchon-ri	2018	37.81159°N, 127.64919°E	S. Podenas	At light, net	NIBR
S. Korea, Doiryung Valley, Bukhansan National Park	2018	37.69037°N, 126.98972°E	H.-Y. Seo, S. Podenas	Net	NIBR
S. Korea, Gyeonggi-do, Yangju-si, Jangheung-myeon, Uldae-ri	2018	37.74258°N, 127.00329°E	A. Petrasiunas	Stream margin	NRC
S. Korea, Gyeonggi-do, Yongpyeong-gun, Cheongun-myeon, Dowon-ri, Dowon Valley	2018	37.54514°N, 127.79449°E	S. Podenas	Net	NIBR
S. Korea, Gyeonggi-do, Gapyeong-gun, Gapyeong-eup, Kalbong Natural Recreation Forest	2018	37.83651°N, 127.46537°E	S. Podenas	Net	NIBR
S. Korea, Gyeonggi-do, Paju-si, Jindong-myeon, 1417 Dongpa-ri, Bonifas	2019	37.92582°N, 126.77410°E	T. A. Klein, H. C. Kim	New Jersey trap	NIBR
NetS. Korea, Gyeonggi-do, Dongducheon, Tapdong-dong, Casey	2019	37.87845°N, 127.14566°E	T. A. Klein, H. C. Kim	New Jersey trap	NIBR
S. Korea, Jeollanam-do, Gurye-gun, Gwangui-myeon, Nogodan-ro	2019	35.29250°N, 127.49548°E	S. Podenas	Net	NIBR
S. Korea, Gyeonggi-do, Yangju-si, Jangheung-myeon, Hoguk-ro	2019	37.71058°N, 126.98719°E	S. Podenas	Net	NIBR
S. Korea, Gyeongsangbuk-do, Yeongju-si, Punggi-eup, Sucheol-ri	2019	36.91772°N, 128.45811°E	C. Lim, C. V. Duong	Net	KUEM
S. Korea, Gyeonggi-do, Paju-si, Jinseo-myeon	2020	37.95433°N, 126.68263°E	T. A. Klein, H. C. Kim	Green-LED	NIBR
S. Korea, Jeju-do, Seogwipo-si, Hawon-dong	2021	33.33516°N, 126.47013°E; 33.34919°N, 126.49536°E	J. Kim, C. Lim, D. Lee	Malaise trap, net	KUEM
S. Korea, Jeju-do, Seogwipo-si, Namwon-eup, Sillye-ri	2021	33.33728°N, 126.62075°E	J. Kim, D. Lee	Malaise trap	KUEM
S. Korea, Gyeongsangbuk-do, Hamyang-gun, Macheon-myeon, Samjeong-ri	2021	35.34214°N, 127.64049°E	J. Kim, C. Lim, D. Lee	Net	KUEM
S. Korea, Jeollabuk-do, Jucheon-myeon, Jinan-gun, Daebul-ri, Site 2	2022	35.97650°N, 127.40115°E		Net, light trap	NIBR

* Coordinates for old collecting sites are approximate.

Together with entomologists from NIBR we started intense studies of Korean crane flies in 2012. Crane flies were collected every year, field work conducted from early spring to late fall in different localities throughout the country; thousands of specimens were collected and put on permanent storage at the NIBR collections. We managed to obtain 784 *Dicranota* specimens from Korea and 22 specimens from surrounding countries for our study. Some of them are new records and even new species.

Adults were collected in various ways, including insect nets, Malaise traps, Green-LED light traps, New Jersey (NJ) traps, and other light sources. The collected specimens were dry-mounted laterally on paper points. Wet specimens were preserved in 96% ethanol (EtOH). Some male wings were slide mounted in Euparal and photographed. Dissected terminalia were cleared in 10% KOH and preserved in microvials with glycerol.

Information on the examined material is given according to the journal requirements, thus altitudes are given in metric system regardless of the system on the label. For specimens collected by SP and his colleagues, the date on the label is followed by a number in brackets. Different localities where insects were collected on the same date were given separate numbers and all information from those localities, whether in the field notes and database, photographs, or other locality information, were marked with this specific number. Specimens are arranged according to the collecting date.

Specimens were examined with an Olympus SZX10 dissecting microscope and Nikon Eclipse Ti microscope. Photographs were taken with a Canon R5 camera through a Canon MP-E 65 mm macro lens and through Mitutoyo M Plan apo 10 × and 20 × lenses mounted on the same camera at NRC.

Total genomic DNA was extracted from the legs of D. (Eudicranota) distincta Podenas, sp. nov. and D. (E.) perdistincta specimens using a QIAamp DNA Micro Kit (Qiagen, Hilden, Germany). Standard PCR amplification and sequencing protocols were used to generate COI fragment sequences. The target fragment of COI was amplified in 20 µL reactions containing AccuPower PCR PreMix (Bioneer Co., Daejeon, Korea), 1 U Top DNA polymerase, dNTPs (10 mM), Tris-HCl (pH 9.0), KCl (30 mM), MgCl2 (1.5 mM), 3 µL (5–50 ng) template DNA, and 1 µL of each primer (LCO1490 and HCO2198; 10 pM each). Amplification was performed using the following thermal cycling program: 94 °C for 4 min; 35 cycles of 94 °C for 0.5 min; 48 °C for 0.5 min; 72 °C for 1 min; and a final extension at 72 °C for 10 min. PCR products were sequenced by Macrogen Inc. (Korea). Before further analysis, DNA sequences for each specimen were aligned in the BioEdit Sequence Alignment Editor. COI sequences were submitted to GenBank: D. (E.) distincta Podenas, sp. nov. has the accession numbers PQ590791 (215 bp) and PQ590791 (398 bp); The D. (E.) perdistincta accession number is PQ590790. Genetic distances between examined species were calculated as proportion of differences (p-distances) as implemented in the program MEGA X.

The terminology of adult morphological features generally follows that of Cumming and Wood (2017), while terminology of wing venation follows de Jong (2017).

## ﻿Taxonomy

### ﻿ Dicranota

Taxon classificationAnimaliaDipteraPediciidae

Zetterstedt, 1838

2C645F50-FFE0-580E-965F-D1BDDF698789

Dicranota Zetterstedt, 1838: 851; Edwards 1938: 51, 58; Ishida 1958: 37; Savchenko and Krivolutskaya 1976: 34; Savchenko 1983: 33; Savchenko 1986: 165; Savchenko 1989: 14, 15.

#### Type species.

*Dicranota guerini* Zetterstedt, 1838 (by monotypy) (Western and Eastern Palaearctic).

#### Description.

Small to medium-sized Pediciidae crane flies with body length 4.3–9.0 mm and wing length 5.0–11.5 mm. Body colouration varies from pale yellow to dark brown, dark grey, or even black.

***Head*.** Rounded posteriorly, vertex with low but wide tubercle. Eyes with short erect setae between ommatidia. Antenna usually short, reaching approximately to frontal margin of prescutum, if bent backwards, sometimes longer in males and reaching posterior margin of first abdominal segment if bent backwards. Number of flagellomeres varies from 10 to 15, most usual number being 11–13. Flagellomeres slightly elongate or oval, covered with short pubescence, verticils variable, but usually not exceeding length of respective segments. Apical antennomere usually large, as long or longer than preceding segment.

***Thorax***. Pronotum rather big, covered with erect setae medially. Mesonotal prescutum without tubercular pits, pseudosutural fovea indistinct. Katepisternum often bare, bearing few setae in some species. Meron small. Middle and posterior coxae close to each other. Wing usually long and narrow, often ~ 4 × as long as wide, similar in both sexes, sometimes dimorphic: posterior margin of male wing extended, widest at tip of anal vein, or wing reduced, with some brachypterous females, sometimes brachypterous individuals occurring among both sexes. Some species, especially living in cold climate or at higher altitudes are all brachypterous. Wings of most species without any pattern, even stigma often is missing. Wings of only few species with smaller or larger dark spots, mostly surrounding cross-veins, base of *Rs* and cord. Venation: *arculus* present, vein *Sc* very long, reaching wing margin far beyond branching point of *Rs*, *sc-r* long distance before base of *Rs*, sometimes close to the middle between base of *Rs* and humeral vein, *R_1_* short, usually not exceeding *R_2_* in length. Radial sector short, ≤ 3 × as long as *m-cu*, often shorter than that. Cell *r_3_* long with short stem or stem is missing completely. Cell *m_1_* usually short, much shorter than its stem. Discal cell missing due to reduction of vein *m-m*, rarely present, but even when present, *m-m* is weak. Cross-vein *m-cu* beyond branching point of *M*. Tip of anal vein far not reaches level of *Rs* base. Anal angle usually wide. Tibial spurs present on all legs. Claw usually simple, without spines.

***Abdomen*.** Male terminalia not enlarged, approximately as wide as rest abdominal segments. Epandrium often with medial and lateral lobes, lateral lobes could be elongate and complicated. Gonocoxite simple or with larger or smaller dorsal lobe at apex. Usually this lobe covered with small dark spines. One or two pairs of gonostyli, outer gonostylus usually bearing lots of small spines, interbase large, often complicate. Aedeagus short and simple, paramere elongate. Ovipositor with long cercus and hypogynial valve, tip of cercus raised upwards, dorsal margin of hypogynial valve usually covered with long and strong setae. Most females with two spermathecae, some with three.

#### Remarks.

The genus *Dicranota* is one of the largest not only among Pediciidae, but among all crane flies. It includes 256 species worldwide (Oosterbroek 2025), two of them with two subspecies each, and the subspecies of a third species raised to species level in this publication. The Palaearctic Region is the most species rich with 104 recognised species, 72 of them occurring in the Eastern and 39 in the Western Palaearctic. This publication adds three more species to the East Palaearctic fauna, now 75, the same number as the Oriental Region. The Nearctic is also rich in *Dicranota*, 69 recognised species; only three species occur in Neotropics. The genus is unknown from Afrotropics and Australian Region. Most species have rather limited distributions, only few have very wide distribution ranges, seven species are known from both Eastern and Western Palaearctic, and four species overlap between the Eastern Palaearctic and Oriental regions. The genus *Dicranota* has 11 recognised subgenera, four of them occurring in Korea. *Dicranota* is poorly represented in fossils with only one species described from the Nearctic Oligocene (Evenhuis 1994) with no subgenus.

##### ﻿List of Korean *Dicranota* Zetterstedt, 1838

Dicranota (Dicranota) coreana Alexander, 1940, stat. nov.

Dicranota (Dicranota) crassicauda Tjeder, 1972

Dicranota (Dicranota) guerini Zetterstedt, 1838

Dicranota (Dicranota) yezoensis Alexander, 1924

Dicranota (Eudicranota) distincta Podenas, sp. nov.

Dicranota (Eudicranota) perdistincta Alexander, 1940

Dicranota (Eudicranota) sibirica sibirica (Alexander, 1925)

Dicranota (Ludicia) emarginata (Alexander, 1945)

Dicranota (Rhaphidolabis) gibbera (Alexander, 1921)

Dicranota (Rhaphidolabis) luteola Alexander, 1938

Dicranota (Rhaphidolabis) minuscula Alexander, 1938

Dicranota (Rhaphidolabis) neoconsors Alexander, 1938

Dicranota (Rhaphidolabis) ompoana Alexander, 1945

Dicranota (Rhaphidolabis) polymera Alexander, 1933

Dicranota (Rhaphidolabis) seoi Podenas, sp. nov.

Dicranota (Rhaphidolabis) squarrosa Savchenko, 1976

Dicranota (Rhaphidolabis) yeongokia Podenas, sp. nov.

##### ﻿Key to Korean *Dicranota*

**Table:** 

1	Fully winged (Figs 1, 2, 9–11, 16, 19–22, 25, 29, 30, 34, 39, 40, 45, 50, 54, 55, 59, 63, 66, 69, 72	**2**
–	Brachypterous (Fig. 26)	**Dicranota (Eudicranota) perdistincta Alexander, 1940** (part)
2	Wing cell *r_2_* with supernumerary cross-vein (Figs 1, 9–11, 16, 19–22, 25, 29, 30)	**3**
–	Wing cell *r_2_* without supernumerary cross-vein (Figs 34, 39, 40, 45, 50, 54, 55, 59, 63, 66, 69, 72)	**9**
3	*Rs* short, 2–3 × as long as *m-cu* (Figs 1, 2, 9–11, 16)	**Dicranota (Dicranota) 4**
–	*Rs* long, ~ 4–5 × as long as *m-cu* (Figs 19–22, 25, 29, 30)	**Dicranota (Eudicranota) 7**
4	Abdomen generally dark brown or grey with yellow spots at most, posterior margin of epandrium nearly straight (Figs 3, 12, 17)	**5**
–	Abdomen yellow or ochraceous, posterior margin of epandrium deeply concave (Fig. 7)	**Dicranota (Dicranota) crassicauda Tjeder, 1972**
5	Male antenna long, reaching distal margin of first abdominal segment if bent backwards, abdomen with distinct yellow spots at postero-lateral corners of tergites. *Rs* ~ 3 × as long as *m-cu*, cell *m_1_* missing or very small (Figs 9–11). Outer gonostylus of male genitalia long and narrow, postero-dorsal lobe of gonocoxite rounded (Figs 12, 13), dorsal apodeme of aedeagus narrow and straight	**Dicranota (Dicranota) guerini Zetterstedt, 1838**
–	Male antenna shorter, not reaching base of halter if bent backwards, abdomen without distinct yellow spots, slightly yellowish at most, usually uniformly brownish grey or brown. *Rs* ~ 2 × as long as *m-cu*, cell *m_1_* present (Figs 1, 2, 16). Outer gonostylus of male genitalia wider, spindle-shaped, postero-dorsal lobe of gonocoxite rounded or elongate, dorsal apodeme of aedeagus narrow with shoulder-like widening or wide, triangle-shaped Figs 5, 18	**6**
6	Male antenna short, not reaching base of wing, if bent backwards; gonocoxite with elongate postero-dorsal lobe (Figs 3, 4), dorsal apodeme of aedeagus wide, triangle-shaped (Figs 5, 18)	**Dicranota (Dicranota) coreana Alexander, 1940, stat. nov.**
–	Male antenna reaching beyond base of wing, if bent backwards; gonocoxite with short rounded postero-dorsal lobe, dorsal apodeme of aedeagus narrow (Figs 17, 18)	**Dicranota (Dicranota) yezoensis Alexander, 1924**
7	Wing with six large spots at frontal margin and small dots scattered along longitudinal veins, especially distinct along vein *CuP* (Figs 29, 30)	**Dicranota (Eudicranota) sibirica sibirica (Alexander, 1925)**
–	Wing with narrow darkening surrounding cross-veins only, larger spots and small dots missing (Figs 21, 22, 25)	**8**
8	Wing cell *m_1_* small, its stem longer than cell itself (Figs 21, 22). Paramere of male genitalia strong, horn-shaped and darkened, meso-dorsal lobe of gonocoxite narrow, subapical (Fig. 23)	**Dicranota (Eudicranota) distincta Podenas, sp. nov.**
–	Wing cell *m_1_* large, longer than its stem (Fig. 25). Paramere of male genitalia terminates in narrow pale rod-shaped elongation, meso-dorsal lobe of gonocoxite large, conical, situated near middle (Fig. 27)	**Dicranota (Eudicranota) perdistincta Alexander, 1940** (part)
9	Wing with closed discal cell, cross-vein *m-m* indistinct, but present; radial sector distinctly branches into *R_2+3_* and *R_4+5_* (Fig. 34). Posterior margin of epandrium with large V-shaped incision (Fig. 36)	**Dicranota (Ludicia) emarginata (Alexander, 1945)**
–	Wing without discal cell, cross-vein *m-m* missing; radial sector branches into *R_2+3+4_* and *R_5_*, or *R_4_* in direct alignment with *Rs* (Figs 39, 40, 45, 50, 54, 55, 59, 63, 66, 69, 72)	**Dicranota (Rhaphidolabis) 10**
10	Darker areas surround cross-veins and tips of longitudinal veins (Figs 39, 40)	**Dicranota (Rhaphidolabis) gibbera (Alexander, 1921)**
–	No darker areas around cross-veins and tips of longitudinal veins (Figs 45, 50, 54, 55, 59, 63, 66, 69, 72)	**11**
11	Wing stigma distinct, brown; dark area extends along cubital vein, indistinct darkening around cord and at distal wing margin. Gonocoxite terminates in large curved spine (Fig. 64)	**Dicranota (Rhaphidolabis) polymera Alexander, 1933**
–	Wing stigma indistinct or missing, wing usually without any darker areas, sometimes indistinct darkening surrounds cubital vein. Apex of gonocoxite without lobes, or lobe simple, round-apexed (Figs 46, 51, 56, 60, 67, 70, 73, 74, 75)	**12**
12	Wing vein *R_2+3+4_* (stem of cell *r_3_*) long, approximately as long as cross-vein *m-cu*	**13**
–	Wing vein *R_2+3+4_* (stem of cell *r_3_*) short, distinctly shorter than cross-vein *m-cu* or totally missing	**14**
13	Dark brown species. Posterior margin of epandrium with large median incision and large single rounded lateral lobe (Fig. 51)	**Dicranota (Rhaphidolabis) minuscula Alexander, 1938**
–	Pale yellow species. Posterior margin of epandrium with small median incision and two or three small tooth-shaped lateral lobes (Figs 73, 74)	**Dicranota (Rhaphidolabis) yeongokia Podenas, sp. nov.**
14	Yellow species. Interbase with bifid apex (Fig. 46)	**Dicranota (Rhaphidolabis) luteola Alexander, 1938**
–	Dark species (brown, dark brown or grey, or at least abdomen and head dark brown). Apex of interbase not bifid (Figs 56, 60, 70)	**15**
15	Pleuron obscure yellow	**Dicranota (Rhaphidolabis) neoconsors Alexander, 1938**
–	Pleuron grey to dark brown	**16**
16	Legs obscure to brownish yellow. Posterior margin of epandrium with distinct median incision (Fig. 70)	**Dicranota (Rhaphidolabis) squarrosa Savchenko, 1976**
–	Legs brown. Posterior margin of epandrium straight or with median lobe (Figs 60, 67)	**17**
17	Antenna 14-segmented. Wing with short vein *R_2+3+4_* (Fig. 66). Posterior margin of epandrium nearly straight with small lateral lobe (Fig. 67)	**Dicranota (Rhaphidolabis) seoi Podenas, sp. nov.**
–	Antenna 13-segmented. Vein *R_2+3+4_* missing (Fig. 59). Posterior margin of epandrium with rectangular median lobe and long curved lateral lobe (Fig. 60)	**Dicranota (Rhaphidolabis) ompoana Alexander, 1945**

##### ﻿Descriptions

### ﻿ Dicranota (Dicranota)

Taxon classificationAnimaliaDipteraPediciidae

Zetterstedt, 1838

89F480D0-8834-5A6A-AE35-97C9ABF11941

Dicranota (Dicranota) : Edwards 1938: 51, 59; Alexander 1950: 18; Ishida 1958: 40; Tjeder 1959: 5; Brindle 1963: 235; Mendl 1972: 150; Savchenko and Krivolutskaya 1976: 45; Savchenko 1983: 39; Savchenko 1986: 188; Savchenko 1989: 17.

#### Type species.

*Dicranota guerini* Zetterstedt, 1838 (by monotypy) (Western and Eastern Palaearctic).

#### Description.

Medium-sized to largest *Dicranota* crane flies with body length 6.3–9.0 mm and wing length 7.0–11.5 mm. Body colouration varies from brownish yellow to dark brown or dark grey.

***Head*.** Antenna 12- or 13-segmented, longer than in most *Dicranota*, reaching at least to approximately middle of presutural scutum, sometimes well beyond base of abdomen, if bent backwards. Male antenna often comparatively longer than that of female. Flagellomeres elongate, verticils short, not exceeding length of respective segments. Apical antennomere usually small, shorter than preceding segment.

***Thorax*.** Presutural scutum with three or four dark longitudinal stripes, medial stripe could have paler narrow line along middle. Wing with comparatively short radial sector, it is only ~ 2.3 × as long as vein *m-cu*. Cell *r_2_* with supernumerary cross-vein. Discal cell usually open due to atrophy of *m-m*, sometimes closed. Cell *m_1_* short to very short, distinctly shorter than its stem, sometimes missing.

***Abdomen*.** Epandrium of male terminalia comparatively simple, posterior margin nearly straight or concave, without additional lobes at the middle, lateral lobe, if present, small and simple. Gonocoxite with blunt, densely setose dorso-apical lobe. Interbase elongate, species specific, often used for species delimitation. Two pairs of gonostyli. Outer gonostylus elongate, fleshy and setose, inner gonostylus pale, elongate, usually covered with small spines and often bearing subbasal lobe. Ovipositor with long cercus and hypogynial valve, tip of cercus raised upwards, dorsal margin of hypogynial valve setose at base, setae comparatively short.

#### Remarks.

The subgenus Dicranota s. str. includes 38 species (subspecies of *D. yezoensis* are treated as separate species here) distributed in the Holarctic and Oriental regions (Oosterbroek 2025). Richest in species are the East Palaearctic and Nearctic faunas, each with 14 species, the Oriental Region has eight, and the West Palaearctic four species. Three species are known from both, West and East Palaearctic.

### ﻿ Dicranota (Dicranota) coreana

Taxon classificationAnimaliaDipteraPediciidae

Alexander, 1940 stat. nov.

0B140524-D2EA-514C-A2C7-4AD27D77DB1C

Figs 1–2, 3–6, 77

Dicranota (Dicranota) yezoensis coreana Alexander, 1940: 45–46.

#### Type material examined.

**North Korea • *Holotype*** ♀ (pinned, antenna and wing slide mounted); Seren Mts.; alt. 853 m; 15 June 1938; A. M. Yankovsky leg.; USNM.

#### Other examined material

**(Fig. 77). North Korea** • 2 ♀ (pinned); Chonsani; alt. 1219 m; 29 April 1940; A. M. Yankovsky leg.; USNM • 2 ♂ (pinned, genitalia in microvials with glycerol on same pins); Chonsani; alt. 1067 m; 27 June 1940; A. M. Yankovsky leg.; USNM • 1 ♂, 1 ♀ (pinned); Chonsani; alt. 1372 m; 29 June 1940; A. M. Yankovsky leg.; USNM • 1 ♂ (pinned); Chonsani; alt. 1219 m; 1 July 1940; A. M. Yankovsky leg.; USNM • 1 ♀ (pinned); Chonsani; alt. 1219 m; 4 July 1940; A. M. Yankovsky leg.; USNM • 1 ♂, 1 ♀ (pinned, ♂ genitalia in microvial with glycerol on same pin); Pontani Paiktusan; alt. 1920 m; 28 July 1940; A. M. Yankovsky leg.; USNM • 1 ♀ (pinned); Pontani Paiktusan; alt. 1524–1829 m; 2 August 1940; A. M. Yankovsky leg.; USNM • 1 ♀ (pinned); Chonsani; alt. 1219 m; 4 August 1940; A. M. Yankovsky leg.; USNM • 1 ♀ (pinned); Pontani Paiktusan; alt. 1920 m; 8 August 1940; A. M. Yankovsky leg.; USNM • 1 ♀ (pinned); Pontani Paiktusan; alt. 1768–1942 m; 9 August 1940; A. M. Yankovsky leg.; USNM • 1 ♂ (pinned, genitalia in microvial with glycerol on same pin); Pontani Paiktusan; alt. 1890 m; 10 August 1940; A. M. Yankovsky leg.; USNM • 1 ♂ (pinned, genitalia in microvial with glycerol on same pin); Pontani Paiktusan; alt. 1920 m; 20 August 1940; A. M. Yankovsky leg.; USNM • 1 ♀ (pinned); Pontani Paiktusan; alt. 1829–1942 m; 25 August 1940; A. M. Yankovsky leg.; USNM • 1 ♀ (pinned); Pontani Paiktusan; alt. 1829–1942 m; 27 August 1940; A. M. Yankovsky leg.; USNM.

#### Redescription.

General body colouration dark brown, male sparsely dusted with grey, female more densely covered with bluish grey pruinosity. Body length of female 7.0–8.7 mm. Wing length of male 7.4–8.2 mm, of female 8.0–9.4 mm.

***Head*.** Brown, light grey along eye margin, darker brown dorso-medially, covered with short yellowish setae dorsally. Eyes widely separated in both sexes, distance between them at base of antennae exceeds length of both basal antennomeres taken together. Antenna 1.1–1.3 mm long in male, reaching to approx. middle of presutural scutum if bent backwards, 1.3 mm in female. Whole antenna uniformly dark brown, scape elongate, nearly twice as long as wide, pedicel subglobular. Flagellum 11-segmented. Basal flagellomere elongate, subcylindrical, nearly twice as long as second. Remaining flagellomeres oval, decreasing in length towards apex of antenna. Flagellum covered with light grey pubescence. Verticils dark brown, not exceeding length of respective segment. Rostrum brown, palpus dark brown, labellum rusty brown.

**Figures 1, 2.**
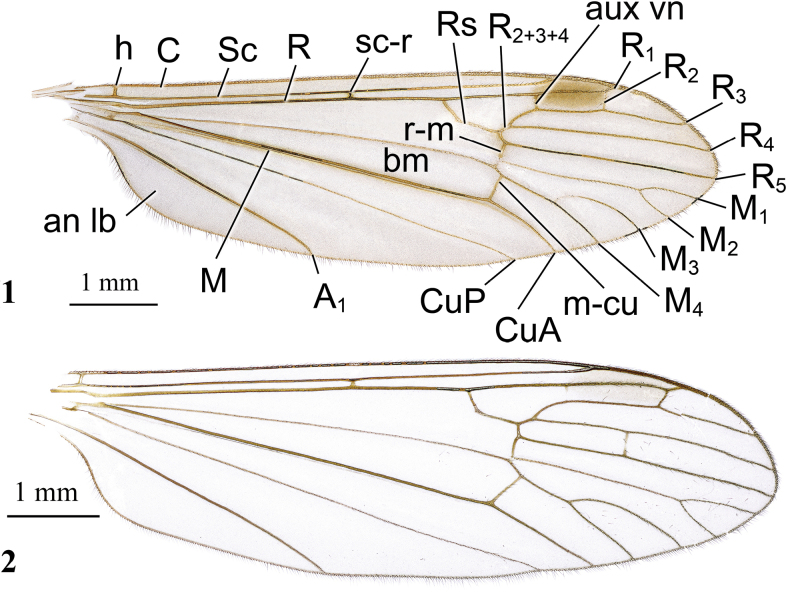
Dicranota (Dicranota) coreana Alexander, 1940, stat. nov. **1** female wing, holotype **2** variation of wing venation, female, right wing (left wing “typical”). Abbreviations: *A_1_* – first branch of anal vein; an lb – anal lobe; aux vn – auxiliary vein (supernumerary cross-vein in cell *r_2_*); *bm* – basal medial cell; *C* – costal vein; *CuA* – anterior branch of cubital vein; *CuP* – posterior branch of cubital vein; *h* – humeral vein; *M* – medial vein, or media; *M_1_* – first branch of media; *M_2_* – second branch of media; *M_3_* – third branch of media; *M_4_* – fourth branch of media; *m-cu* – medial-cubital cross-vein; *R* – radius, or radial vein; *R_1_* – anterior branch of radius; *R_2_* – second branch of radius; *R_2+3+4_* – stem of radial branches *R_2_*, *R_3_* and *R_4_*; *R_3_* – lower branch of second branch of radius; *R_4_* – upper branch of third branch of radius; *R_5_* – lower branch of third branch of radius; *r-m* – radio-medial cross-vein; *Rs* – radial sector; *Sc* – subcostal vein; *sc-r* – subcostal-radial cross-vein.

***Thorax*.** Dark brown, covered with grey pruinosity. Pronotum covered with erect straight pale yellow setae. Presutural scutum densely covered with greyish brown pruinosity, with four distinct dark brown stripes. Median stripes not reaching posterior margin of sclerite, narrowly separated along middle by very narrow light vitta. Lateral stripe short. Stripes without darker margins. Tubercular pits missing, pseudosutural fovea indistinct. Scutal lobe greyish brown with longitudinal dark brown stripe along middle. Area between scutal lobes brownish grey. Scutellum bluish grey. Mediotergite brownish grey, paler grey anteriorly, darker posteriorly. Pleuron uniformly brownish grey, semi-polished brown where pruinosity has been denuded. Prothoracic spiracle surrounded by brownish yellow membrane. Wing (Figs 1, 2) widest before tip of vein *CuP*, translucent, strongly iridescent with greyish tinge. Stigma brown, elongate. Dark spot at base of *R_5_* and cross-vein *r-m* indistinct, no darkening at base of *Rs*. Veins brown, pale at wing base. Venation: *Sc* long, tip nearly reaching middle of stigma, *sc-r* far before base of *Rs*, beyond level of *A_1_* tip. *Rs* short, usually angulated, often with short spur. Free end of *R_1_* very short, distinctly shorter than *R_2_*. Vein *R_2_* transverse, at distal margin of stigma, supernumerary cross-vein in cell *r_1_* at frontal margin of stigma. Distal parts of *R_3_*, *R_4_*, and *R_5_* nearly parallel to each other. Cell *r_3_* with short stem. Cross-vein *r-m* distinct, discal cell open by atrophy of vein *m-m*. Cell *m_1_* distinct, rarely small, present in all studied specimens, length slightly varies individually. Cross-vein *m-cu* slightly beyond branching point of *M*, *CuP* straight, anal vein nearly straight. Anal angle widely rounded. Length of male halter 1.0–1.1 mm, of female 1.0 mm. Halter pale brown, knob slightly darker than stem. Coxae yellow, dusted with grey, covered with sparse yellowish setae. Fore and middle coxae darker brown at base, more darkened frontally. Trochanters obscure yellow. Femora brownish, paler at base, darker towards apex. Tibiae brown, slightly darker towards distal end, tarsomeres dark brown. Male femur I: 4.8 mm long, II: 5.0–5.6 mm, III: 5.2–5.4 mm, tibia I: 4.9 mm, II: 4.5–5.0 mm, III: 4.8–5.4 mm, tarsus I: 6.5 mm, II: 5.5–6.0 mm, III: 5.6 mm. Female femur I: 4.2–4.5 mm long, II: 4.4 mm, III: 5.0 mm, tibia I: 4.2–4.3 mm, II: 4.2 mm, III: 4.8 mm, tarsus I: 4.9–5.3 mm, II: 4.7 mm, III: 5.3 mm. Claw simple, without spines, rusty brown.

**Figures 3–6.**
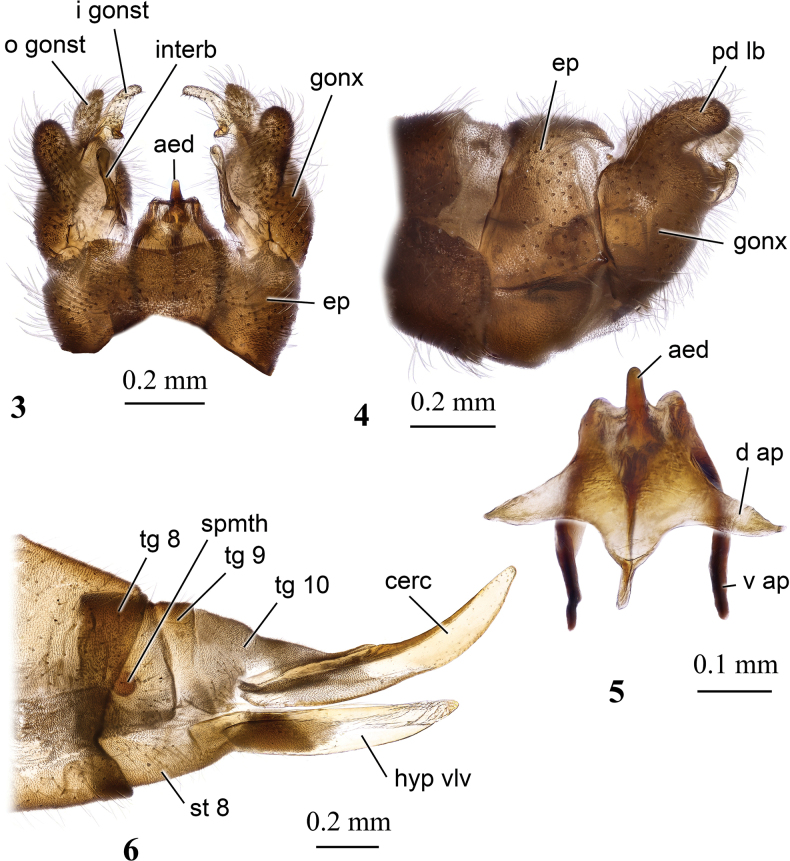
Dicranota (Dicranota) coreana Alexander, 1940, stat. nov. **3** male genitalia, dorsal view **4** male genitalia, lateral view **5** aedeagal complex, dorsal view **6** ovipositor, lateral view. Abbreviations: aed – aedeagus; cerc – cercus; d ap – dorsal apodeme; ep – epandrium; gonx – gonocoxite; hyp vlv – hypogynial valve; i gonst – inner gonostylus; interb – interbase; pod lb – postero-dorsal lobe of gonocoxite; o gonst – outer gonostylus; spmth – spermatheca; st – sternite; tg – tergite; v ap – ventral apodeme.

***Abdomen*.** Male abdomen dark brown, sparsely dusted with grey, posterior margins of segments narrowly pale or greyish, lateral margins of tergites indistinctly rusty brown towards distal end of sclerite. Female abdomen with denser cover of grey or bluish grey pruinosity, posterior margins of sternites very narrowly pale.

***Male terminalia*** (Figs 3–5) rusty brown to dark brown. Posterior margin of epandrium concave, postero-lateral angle rounded. Gonocoxite short and wide with elongate slightly arched round-apexed setose postero-dorsal lobe better discernible in lateral view (Fig. 4). Interbase long and narrow, rod-shaped. Outer gonostylus fleshy and setose, spindle-shaped. Inner gonostylus elongate, pale, mesal margin with small spines and darkened subbasal bump. Aedeagus (Fig. 5) with elongate distal part and with two pairs of lateral apodemes, dorsal apodemes wide, wing-shaped. Posterior segments of female abdomen concolourous with the rest of abdomen, base of ovipositor darker brown.

***Female terminalia*** (Fig. 6). Cercus and hypogynial valve brownish yellow, cercus just slightly arched, blunt-apexed. Hypogynial valve straight, blackish at base, apex pale and acute. Spermatheca small, rounded.

#### Elevation range.

From 850 m to ca 1900 m.

#### Period of activity.

Adults fly from late April through to end of August.

#### Habitat.

Unknown.

#### General distribution.

Species endemic to northern Korea.

#### Remarks.

Male was unknown, it is described for the first time herein. The taxon should be treated as a separate species because *D. yezoensis* Alexander, 1924 also occurs in northern Korea and they have differences in the male genitalia, like the shape of the gonocoxite and details of the aedeagus, especially the shape of the dorsal apodeme of the aedeagus. The male antenna of *D. coreana* is shorter than that of *D. yezoensis*.

### ﻿ Dicranota (Dicranota) crassicauda

Taxon classificationAnimaliaDipteraPediciidae

Tjeder, 1972

786F2F84-D4CA-5C51-A119-879215F3539B

Figs 7, 8, 78

Dicranota (Dicranota) crassicauda Tjeder, 1972: 223–228, figs 1–13.

#### Examined material

**(Fig. 78). North Korea** • 1 ♂ (pinned, genitalia in microvial with glycerol on same pin); Pontani Paiktusan; alt. 1768–1942 m; 9 August 1940; A. M. Yankovsky leg.; USNM.

#### Redescription.

General body colouration brownish yellow with grey thorax and yellowish wings. Male body length ~ 8.0 mm. Male wing length 7.0–10.5 mm, that of female 10.0–11.5 mm.

***Head*.** Dark brown, narrowly whitish along eye margin, wider posteriorly. Vertex with large knob-shaped tubercle. Antenna dark brown, nearly black, ~ 2.5 mm long in male. Scape short, approximately as long as wide, pedicel slightly shorter and narrower than scape. Flagellum 11-segmented in male, 10-segmented in female. Basal flagellomere elongate, exceeding in length both basal antennomeres, subcylindrical. Remaining flagellomeres elongate, decreasing in length towards apex of antenna, segments 2–10 moderately swollen at both ends and slightly narrower at middle in male, segments 2–4 slightly elongate, 5–9 oval or subglobular in female, apical segment conus-shaped, larger in male, small in female. Flagellum covered with short dense pubescence. Verticils very short, approximately as long as sparse trichia covering flagellomeres. Palpus yellowish brown, 4-segmented, basal palpomere small subglobular, second elongate, third palpomere distinctly swollen, apical segment small, rounded.

***Thorax*.** Densely dusted with grey. Cervical sclerites brownish, pronotum dark brown with narrowly grey posterior margin, sparsely covered with short erect yellowish setae. Presutural scutum with four distinct dark brown stripes. Median stripes not reaching posterior margin of sclerite, narrowly separated along middle by very narrow light vitta. Lateral stripe short, reaching suture. Stripes without darker margins. Tubercular pits missing, pseudosutural fovea indistinct. Scutal lobe grey with large dark brown spot at middle. Area between scutal lobes whitish to pale grey. Scutellum because of denuded pruinosity dark brown. Mediotergite whitish to pale grey frontally, brownish grey posteriorly, fronto-lateral corner yellowish. Pleuron uniformly pale grey with yellowish anepisternum. Prothoracic spiracle surrounded by brownish yellow membrane, sparsely dusted with grey. Wing translucent with brownish tinge, yellowish at base, iridescent, widest at or slightly before tip of vein *CuP*. Stigma pale brown, elongate. Dark pattern includes pale brownish spots at base of *Rs* and along cord. Veins brown, yellowish at wing base. Venation: *Sc* long, tip reaching base of stigma, *sc-r* far before base of *Rs*, slightly beyond level of *A_1_* tip. *Rs* medium-long (similar to that of *D. guerini*), slightly arcuate at base. Free end of *R_1_* very short, distinctly shorter than *R_2_*. Vein *R_2_* transverse, at distal margin of stigma, supernumerary cross-vein in cell *r_1_* at frontal margin of stigma. *R_3_*, *R_4_*, and *R_5_* parallel to each other. Cell *r_3_* with short stem. Cross-vein *r-m* distinct, discal cell open by atrophy of vein *m-m*. Cell *m_1_* small. Cross-vein *m-cu* slightly beyond branching point of *M*, veins *CuP* and *A_1_* slightly arched. Anal angle widely rounded. Length of male halter 1.2–1.5 mm. Stem of halter ochraceous, knob brownish. Fore coxa yellowish grey, mid-coxa greyish at base, yellowish distally, posterior coxa brownish yellow. Trochanters obscure to brownish yellow. Femora yellowish brown with paler base, tip without darkening. Tibiae brown, tarsi dark brown with paler base of basitarsus. Male femur II: 5.0 mm, III: 5.6 mm, tibia II: 5.1 mm, III: 5.1 mm, tarsus II: 6.6 mm. Claw simple, without spines, dark brown at base, pale at apex, slightly arched.

***Abdomen*.** Tergites semi-polished, yellow to ochraceous, posterior margin indistinctly and narrowly greyish. Sternites pale brownish yellow. Two basal sternites with denser cover of greyish pruinosity.

**Figures 7, 8.**
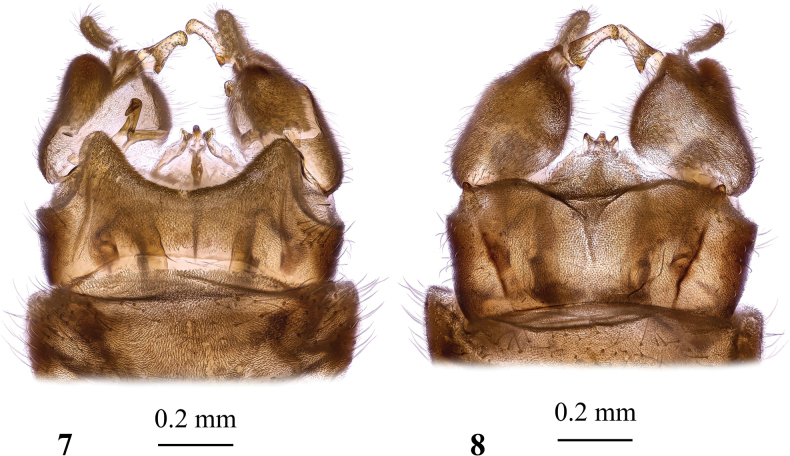
Male genitalia of Dicranota (Dicranota) crassicauda Tjeder, 1972 **7** dorsal view **8** ventral view.

***Male terminalia*** (Figs 7, 8). Terminalia brown. Posterior margin of epandrium deeply and widely concave, postero-lateral angle rounded at tip in KOH cleared genitalia, rather acute in dry specimen. Gonocoxite short and wide with rounded setose postero-dorsal lobe. Interbase long and narrow with hook-shaped apical part. Outer gonostylus fleshy and setose, elongate, finger-shaped. Inner gonostylus elongate, pale, mesal margin with short spine-shaped setae and darkened subbasal angulate lobe. Distal part of aedeagus slightly elongate, directed upwards. Female with two spermathecae, ovipositor with short stout cercus and nearly straight hypogynial valve.

#### Elevation range.

Circa 1800–1900 m in Korea.

#### Period of activity.

Single specimen was captured at the beginning of August in northern Korea.

#### Habitat.

Unknown in Korea. Scandinavian specimens were collected at margins of small cold lakes and streams at high altitudes above tree limit, on moist ground covered with willows and sedges (Tjeder 1972).

#### General distribution.

Species occurs both in Eastern and Western Palaearctic, it is recorded from Armenia, Finland, eastern Kazakhstan, Norway, Sweden, and Tajikistan. Recorded from Korean Peninsula for the first time.

#### Remark.

Female characters are based on Tjeder (1972). The finding of *D. crassicauda* in Korea was unexpected, because the distribution area of this species is much further west; the closest known locality is in eastern Kazakhstan.

### ﻿ Dicranota (Dicranota) guerini

Taxon classificationAnimaliaDipteraPediciidae

Zetterstedt, 1838

E14982E5-80FE-58ED-A1A0-CF82CFF0F5F7

Figs 9–15, 79

Dicranota guerini Zetterstedt, 1838: 851; Lundström 1912: 65; Edwards 1921: 225; Meijere 1921: 26; Pierre 1924: 137; Czižek 1931: 175; Lackschewitz 1940: 111, 113; Fahy 1972: 260–263.
?Dicranota galactoptera Alexander, 1927: 5–6.
Dicranota (Dicranota) guerini : Edwards 1938: 59; Coe 1950: 137; Starý and Rozkošný 1970: 101.

#### Examined material

**(Fig. 79). North Korea** • 1 ♀ (pinned); Chonsani; alt. 1219 m; 29 April 1940; A. M. Yankovsky leg.; USNM • 3 ♂ (pinned); Chonsani; alt. 1524 m; 26 June 1940; A. M. Yankovsky leg.; USNM • 5 ♂ (pinned, genitalia in microvials with glycerol on same pins); Chonsani; alt. 1067 m; 27 June 1940; A. M. Yankovsky leg.; USNM • 5 ♂ (pinned, genitalia in microvials with glycerol on same pins); Chonsani; alt. 1219 m; 27 June 1940; A. M. Yankovsky leg.; USNM • 1 ♂ (pinned); Chonsani; alt. 1067 m; 29 June 1940; A. M. Yankovsky leg.; USNM • 2 ♀ (pinned); Chonsani; alt. 1219 m; 1 July 1940; A. M. Yankovsky leg.; USNM • 2 ♂ (pinned); Chonsani; alt. 1524 m; 4 July 1940; A. M. Yankovsky leg.; USNM • 1 ♀ (pinned); Chonsani; alt. 1219 m; 4 July 1940; A. M. Yankovsky leg.; USNM • 2 ♂, 1 ♀ (pinned); Pontani Paiktusan; alt. 1372 m; 17 July 1940; A. M. Yankovsky leg.; USNM • 3 ♂ (pinned); Pontani Paiktusan; alt. 1676 m; 28 July 1940; A. M. Yankovsky leg.; USNM • 2 ♂ (pinned); Pontani Paiktusan; alt. 1524–1829 m; 28 July 1940; A. M. Yankovsky leg.; USNM • 4 ♂ (pinned, genitalia of 1 ♂ in microvial with glycerol on same pin); Pontani Paiktusan; alt. 1676 m; 31 July 1940; A. M. Yankovsky leg.; USNM • 1 ♂ (pinned); Pontani Paiktusan; alt. 1676–1920 m; 1 August 1940; A. M. Yankovsky leg.; USNM • 1 ♂ (pinned); Chonsani; alt. 1067 m; 5 August 1940; A. M. Yankovsky leg.; USNM • 2 ♂ (pinned); Pontani Paiktusan; alt. 1890 m; 5 August 1940; A. M. Yankovsky leg.; USNM • 1 ♂ (pinned); Pontani Paiktusan; alt. 1890 m; 6 August 1940; A. M. Yankovsky leg.; USNM • 1 ex. (pinned, tip of abdomen broken); Pontani Paiktusan; 6 August 1940; A. M. Yankovsky leg.; USNM • 2 ♂, 1 ex. (pinned, tip of 1 ex. abdomen broken); Pontani Paiktusan; alt. 1768–1942 m; 8 August 1940; A. M. Yankovsky leg.; USNM • 1 ♀ (pinned); Pontani Paiktusan; alt. 1829 m; 8 August 1940; A. M. Yankovsky leg.; USNM • 1 ♂ (pinned); Pontani Paiktusan; alt. 1859 m; 8 August 1940; A. M. Yankovsky leg.; USNM • 1 ♂ (pinned); Pontani Paiktusan; alt. 1859 m; 9 August 1940; A. M. Yankovsky leg.; USNM • 1 ♂ (pinned, genitalia in microvial with glycerol on same pin); Pontani Paiktusan; alt. 1920 m; 10 August 1940; A. M. Yankovsky leg.; USNM • 1 ♂ (pinned); Pontani Paiktusan; alt. 1859 m; 10 August 1940; A. M. Yankovsky leg.; USNM • 3 ♂ (pinned, genitalia of 1 ♂ in microvial with glycerol on same pin); Pontani Paiktusan; alt. 1890 m; 10 August 1940; A. M. Yankovsky leg.; USNM • 1 ♂ (pinned, genitalia in microvial with glycerol on same pin); Pontani Paiktusan; alt. 1942 m; 13 August 1940; A. M. Yankovsky leg.; USNM • 1 ♂ (pinned); Pontani Paiktusan; alt. 1829 m; 17 August 1940; A. M. Yankovsky leg.; USNM • 2 ♀ (pinned); Pontani Paiktusan; alt. 1920 m; 18 August 1940; A. M. Yankovsky leg.; USNM • 1 ♂ (pinned); Pontani Paiktusan; alt. 1920 m; 20 August 1940; A. M. Yankovsky leg.; USNM • 1 ♂ (pinned); Pontani Paiktusan; alt. 1942 m; 23 August 1940; A. M. Yankovsky leg.; USNM • 1 ♂ (pinned, genitalia in microvial with glycerol on same pin); Pontani Paiktusan; alt. 1829–1942 m; 24 August 1940; A. M. Yankovsky leg.; USNM • 1 ♀ (pinned); Pontani Paiktusan; alt. 1829–1942 m; 27 August 1940; A. M. Yankovsky leg.; USNM; **Mongolia** • 1 ♂ (pinned, genitalia in microvial with glycerol on same pin); Bayan-Olgiy Aimag, Nogoonnuur Soum, Zakhin Us Gol, 15 km W Nogoonnuur; 49.57774°N, 90.03497°E; alt. 1764 m; 8–9 July 2010; S. Podenas leg.; MAIS 2010070803 (ANSP) • 1 ♂ (pinned, genitalia in microvial with glycerol on same pin); Uvs Aimag, Turgen Soum Springs S side of Khondlon Gol, 6 km W Turgen; 50.07458°N, 91.60140°E; alt. 1316 m; 14 July 2010; S. Podenas leg.; MAIS 2010071401 (ANSP) • 1 ♂ (pinned, genitalia in microvial with glycerol on same pin); Uvs Aimag, Turgen Soum, Javartain Gol & Turgen Gol, 33 km SW Turgen; 49.89234°N, 91.35239°E; alt. 1849 m; 16–17 July 2010; S. Podenas leg.; MAIS 2010071602 (ANSP).

#### Redescription.

General body colouration greyish brown densely covered with grey pruinosity. Body length of male 7.0–7.5 mm, of female 7.6–9.0 mm. Wing length of male 7.1–8.5 mm, of female 9.3–9.5 mm.

***Head*.** Densely dusted with greyish brown pruinosity, covered with short erect yellowish setae posteriorly, naked frontally. Eyes widely separated in both sexes, distance between them at base of antennae exceeds length of both basal antennomeres taken together. Antenna 2.7–3.3 mm long in male, reaching posterior margin of first abdominal segment if bent backwards, 1.5 mm in female, reaching slightly before middle of presutural scutum if bent backwards. Whole antenna uniformly dark brown, scape elongate, nearly twice as long as wide, pedicel subglobular. Flagellum 11-segmented in male, 10-segmented in female. Male flagellomeres elongate, nearly cylindrical, ~ 4 × as long as wide, decreasing in length towards apex of antenna, densely covered with short, dense and erect light grey pubescence. Basal female flagellomere elongate, remaining segments oval, pubescence very short. Verticils dark brown, short, ≤ 0.5 × as long as width of flagellomere in male, as long as width of flagellomere in female. Rostrum brown dusted with grey, palpus pale brown to brown in male, darker in female. Labellum pale brown.

**Figures 9–15.**
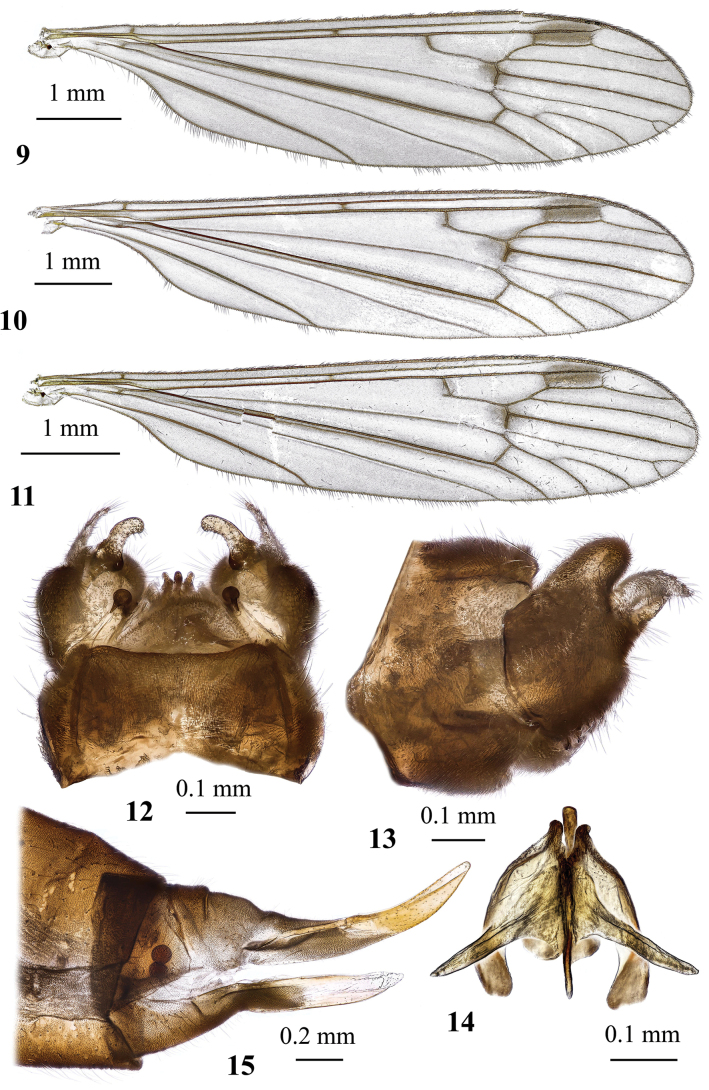
Dicranota (Dicranota) guerini Zetterstedt, 1838 **9** wing **10** wing, variation of venation **11** wing, variation of venation **12** male genitalia, dorsal view **13** male genitalia, lateral view **14** aedeagal complex, dorsal view **15** ovipositor, lateral view.

***Thorax*.** Cervical sclerites yellowish brown. Pronotum brownish grey covered with erect greyish setae. Presutural scutum densely covered with brownish grey pruinosity, with three distinct dark brown stripes. Median stripe wide, separated along middle by very narrow light vitta. Lateral stripe short. Stripes without darker margins. Tubercular pit and pseudosutural fovea indistinct. Scutal lobe dark brown laterally, grey medially. Area between scutal lobes pale grey posteriorly. Scutellum greyish frontally, brownish posteriorly. Mediotergite greyish with narrowly pale brown frontal margin and darker posterior part. Pleuron uniformly brownish grey, darker brown where pruinosity has been denuded. Prothoracic spiracle surrounded by pale brown membrane. Wing (Figs 9–11) long and narrow, widest at tip of vein *CuP*, translucent, strongly iridescent with light brownish tinge. Stigma distinct, elongate, brown to dark brown. Distinct dark spot surrounds base of *R_5_* and cross-vein *r-m*. Indistinct darker area surrounds base of *Rs*. Veins dark brown, pale at wing base. Venation: *Sc* long, reaching level of stigma, *sc-r* far before base of *Rs*, close to the level of tip of anal vein, but position slightly varies individually from before tip of *A_1_* (Fig. 9) to slightly beyond it (Figs 10, 11). *Rs* medium long, more often arched at base (Fig. 9), but it could be angulate and short-spurred (Figs 10, 11), or angulate without spur. Free end of *R_1_* longitudinal, as long or shorter than *R_2_*. Vein *R_2_* transverse, at distal margin of stigma, supernumerary cross-vein in cell *r_1_* at frontal margin of stigma. *R_3_*, *R_4_*, and *R_5_* nearly parallel to each other distally. Cell *r_3_* with short stem. Cross-vein *r-m* distinct, discal cell open by atrophy of *m-m* vein, closed in rare occasions. Cell *m_1_* usually missing or very small, length slightly varies, sometimes missing on one wing, but present on other. Cross-vein *m-cu* slightly beyond branching point of *M*, *CuP* straight, anal vein slightly arched, nearly straight. Anal angle widely rounded. Length of male halter 0.9–1.1 mm, of female 1.1 mm. Stem of halter pale brownish grey with pale base, knob slightly infuscate. Coxae greyish pale brown or brownish yellow, fore and middle coxae darker brown at base, more darkened frontally. Trochanters brownish yellow with slightly darkened distal margin. Femora brownish with pale base, distal margin not darker than remainder of femur. Tibiae and basal tarsomeres brown, distal tarsomeres dark brown. Male femur I: 4.4–5.0 mm long, II: 4.5–4.9 mm, III: 4.8–5.3 mm, tibia I: 4.3–4.8 mm, II: 4.2–4.7 mm, III: 4.9 mm, tarsus I: 6.3–7.3 mm, II: 6.3 mm, III: 6.4–6.6 mm. Female femur III: 4.9 mm long, tibia III: 4.9 mm. Claw simple, without spines.

***Abdomen*.** Tergites of male abdomen dark brown with narrowly pale yellow posterior and lateral margins and widely yellow postero-lateral corners, leaving only narrow dark line between them along the middle of sclerite. Sternites with brown basal half and yellow posterior, narrowly pale grey along posterior margin. Female abdomen with less distinct pattern, tergites greyish brown, darker along middle, sternites yellowish brown. Posterior margins of segments narrowly pale.

***Male terminalia*** (Figs 12–14) brown to dark brown, not distinctly darker than preceding segments. Posterior margin of epandrium slightly concave, postero-lateral angle with small bump. Gonocoxite short and wide with large rounded setose postero-dorsal lobe better discernible in lateral view (Fig. 13). Interbase long and narrow, widened subapically, tip spine-shaped. Outer gonostylus pale, unusually for the genus, long and narrow, fleshy and setose. Inner gonostylus sausage-shaped, slightly arched with bluntly rounded tip, mesal margin setose. Aedeagus (Fig. 14) short with two pairs of lateral apodemes, dorsal apodemes long and narrow, making together V-shaped structure. Posterior segments of female abdomen concolourous with the rest of abdomen.

***Ovipositor*** (Fig. 15). Darkened at base. Cercus just slightly arched, blunt-apexed, distal part yellow. Hypogynial valve straight, blackish at base, distal part pale, dorsal margin with short arched setae at base. Spermatheca small and rounded.

#### Elevation range.

From 1000 m to nearly 2000 m.

#### Period of activity.

From late April through to end of August.

#### Habitat.

Larvae develop in small springs, rivulets, streams, and fast running medium-sized rivers with sand or gravel on the bottom. Usually more abundant in small springs and rivulets. Larvae predacious.

#### General distribution.

Wide-spread throughout the whole Palaearctic.

#### Remark.

This species is recorded from the Korean Peninsula for the first time.

### ﻿ Dicranota (Dicranota) yezoensis

Taxon classificationAnimaliaDipteraPediciidae

Alexander, 1924

CAA400C2-B20D-57F3-BE3B-467F0DF65595

Figs 16–18, 80

Dicranota yezoensis Alexander, 1924: 571.
Dicranota (Dicranota) yezoensis : Savchenko and Krivolutskaya 1976: 47, fig. 17b; Savchenko 1989: 18.

#### Type material examined.

**Japan • *Holotype*** ♂ (pinned, wing and genitalia slide mounted); Hokkaido, Akan; 4 September 1922; T. Esaki leg.; USNM.

#### Other examined material

**(Fig. 80). North Korea** • 1 ♂ (antenna, hind leg, wing and abdomen slide mounted); Kankyo Nando, Puksu Pyaksan; alt. 1219 m; 14 August 1939; A. M. Yankovsky leg.; C. P. Alexander det.; USNM.

#### Redescription.

General body colouration dark brownish grey. Body length of male 6.3 mm, wing length 7.0–8.1 mm.

***Head*.** Brownish grey, narrowly pale grey along eye margin. Male antenna reaching to approx. middle between base of wing and halter if bent backwards. Antennal flagellum 10-segmented, uniformly shiny black, basal flagellomere elongate, succeeding flagellomeres oval, decreasing in length towards apex of antenna. Flagellum covered with white pubescence, apical segment very small.

**Figures 16–18.**
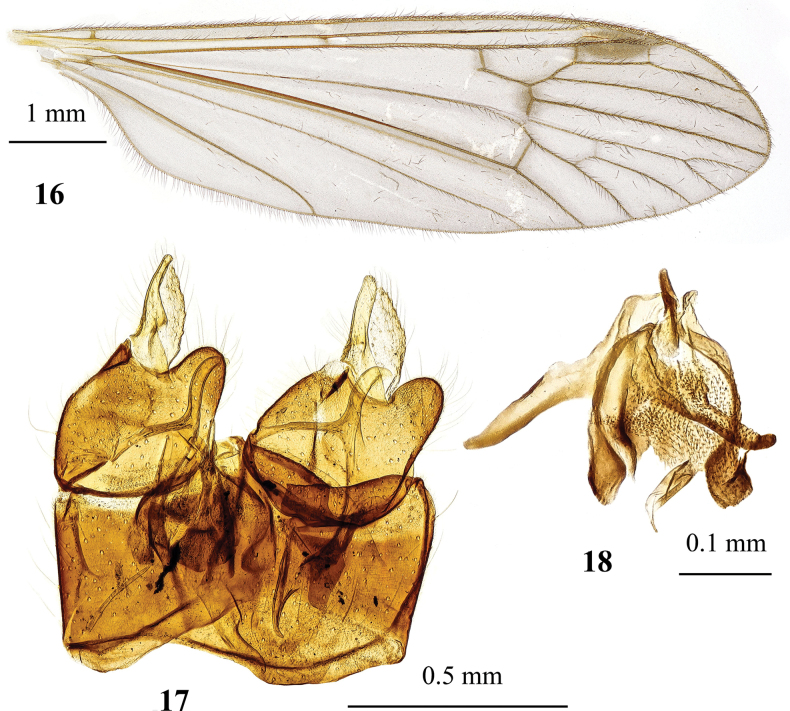
Dicranota (Dicranota) yezoensis Alexander, 1924 **16** male wing, holotype **17** male genitalia, dorsal view, holotype **18** aedeagal complex, dorsal view.

***Thorax*.** Dark brownish grey. Presutural scutum with three darker stripes. Median stripe with narrow light vitta along middle. Scutal lobe grey with pale brown spot. Scutellum and mediotergite light grey. Pleuron uniformly pale grey. Wing (Fig. 16) widest at tip of vein *CuP*, translucent, strongly iridescent, with brownish tinge. Stigma distinct, dark brown, elongate. Dark spot at base of *R_5_* and cross-vein *r-m* distinct, less distinct darkening at base of *Rs*. Veins brown, yellowish at wing base. Venation: *Sc* long, tip reaching wing margin beyond base of stigma, *sc-r* short distance beyond level of *A_1_* tip. *Rs* short, angulated and short-spurred. Free end of *R_1_* nearly as long as *R_2_*. Vein *R_2_* transverse, at distal margin of stigma, supernumerary cross-vein in cell *r_1_* at frontal margin of stigma. *R_3_*, *R_4_*, and *R_5_* nearly parallel to each other distally. Cell *r_3_* with short stem. Cross-vein *r-m* distinct. Discal cell present in holotype, but cross-vein *m-m* very weak. Korean specimen has open discal cell with only part of *m-m* present. Cell *m_1_* large. Cross-vein *m-cu* 1/2–2/3 of its own length beyond branching point of *M*, *CuP* slightly sinuous, nearly straight, anal vein slightly arched. Anal angle widely rounded. Length of male halter 0.9 mm. Halter brown with paler stem. Coxae light grey with yellowish distal part. Trochanters dull yellow with blackened distal margin. Femora brown, paler at base, darker towards apex. Tibiae brown, slightly darker towards distal end, tarsomeres dark brown. Male femur I: 3.8 mm long, II: 3.5 mm, III: 4.3 mm, tibia I: 4.5 mm, II: 4.8 mm, III: 4.8 mm, tarsus I: 5.6 mm, II: 4.9 mm, III: 4.7 mm.

***Abdomen*.** Dark brownish grey, tergites with narrowly pale caudal margin, sternites with yellowish grey caudal margin. Abdomen ventrally with indistinct broken median yellowish stripe.

***Male terminalia*** (Figs 17, 18). Epandrium with caudal margin slightly sinuous, postero-lateral angle without additional structures. Gonocoxite short and wide with rounded setose postero-dorsal lobe. Interbase long and narrow, slightly arched. Outer gonostylus fleshy and setose, spindle-shaped. Inner gonostylus elongate, pale, with low wide lobule at ~ 1/3 of gonostylus length on mesal margin. Aedeagus (Fig. 18) with elongate distal part and with two pairs of lateral apodemes, dorsal apodeme narrow, similar to that of *D. guerini*, but with distinct shoulder.

#### Elevation range.

Korean specimen was taken slightly above 1200 m.

#### Period of activity.

Adults fly from middle of August to beginning of September.

#### Habitat.

Unknown.

#### General distribution.

Species was recorded from Hokkaido Island (Japan), Sakhalin and Kuril Islands (Russia).

#### Remark.

Species was recorded only on islands, it was unknown from the continent. Species recorded from the Korean Peninsula for the first time.

### ﻿ Dicranota (Eudicranota)

Taxon classificationAnimaliaDipteraPediciidae

Alexander, 1934

AAC92B0E-BEF0-5810-B099-C6B3CFDDCE74

Dicranota (Eudicranota) Alexander in Curran 1934: 46; Alexander 1950: 17; Ishida 1958: 40; Savchenko and Krivolutskaya 1976: 35; Savchenko 1983: 39; Savchenko 1989: 18–20.
Dicranota (Dicranotella) Alexander 1950: 18.

#### Type species.

*Dicranota notabilis*Alexander 1929 (original designation) (Nearctic).

#### Redescription.

Small to medium-sized pale yellow or whitish yellow *Dicranota* crane flies with body length 5.0–8.7 mm and wing length 5.0–7.5 mm.

***Head*.** Antenna 12–13-segmented, reaching to approx. or slightly beyond frontal margin of presutural scutum, if bent backwards. Antennal verticils long, at least as long as respective segments, usually longer.

***Thorax*.** Presutural scutum could be uniformly coloured, without dark longitudinal stripes, sometimes just with indistinct medial stripe and sometimes with three indistinct stripes. Wing with at least narrow dark areas surrounding cross-veins and base of *Rs*, radial sector comparatively long, it is 4–5 × as long as vein *m-cu*. Cell *r_2_* with supernumerary cross-vein. Discal cell present, cell *m_1_* rather long, as long or longer as its stem.

***Abdomen*.** Posterior margin of epandrium extended medially, lateral lobe long and narrow, slightly curved. Gonocoxite of male terminalia simple, or with distinct dorso-medial lobe. Interbase usually large, elongate. One or two pairs of gonostyli. Outer gonostylus or tip and outer margin of single gonostylus covered with small black spines. Ovipositor with long cercus and hypogynial valve, tip of cercus just slightly raised upwards, dorsal margin of hypogynial valve covered with long strong setae, tips of which reaching or nearly reaching apex of valve. Spermathecae three, small, drop-shaped.

#### Remarks.

The subgenus Dicranota (Eudicranota) includes 16 species (one species is added in this publication, one species with two subspecies). Subgenus most diverse in the Eastern Palaearctic with nine species (one with two subspecies), four species recorded from the Nearctic, and three from the Oriental regions (Oosterbroek 2025).

### ﻿ Dicranota (Eudicranota) distincta

Taxon classificationAnimaliaDipteraPediciidae

Podenas sp. nov.

510603B8-E44B-5159-9D69-04211BA43700

https://zoobank.org/1B05A03D-5F71-4502-AD2F-AB01066CD49F

**Figures 19, 20.**
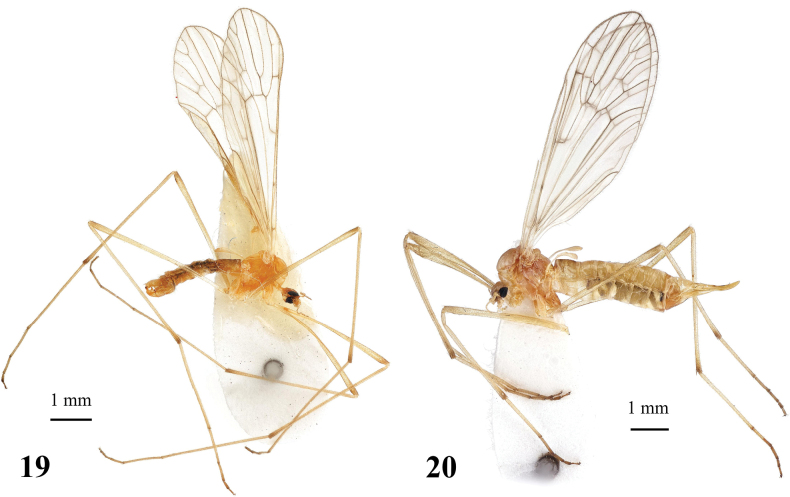
Dicranota (Eudicranota) distincta Podenas, sp. nov. **19** male, holotype **20** female, paratype.

**Figures 21–24.**
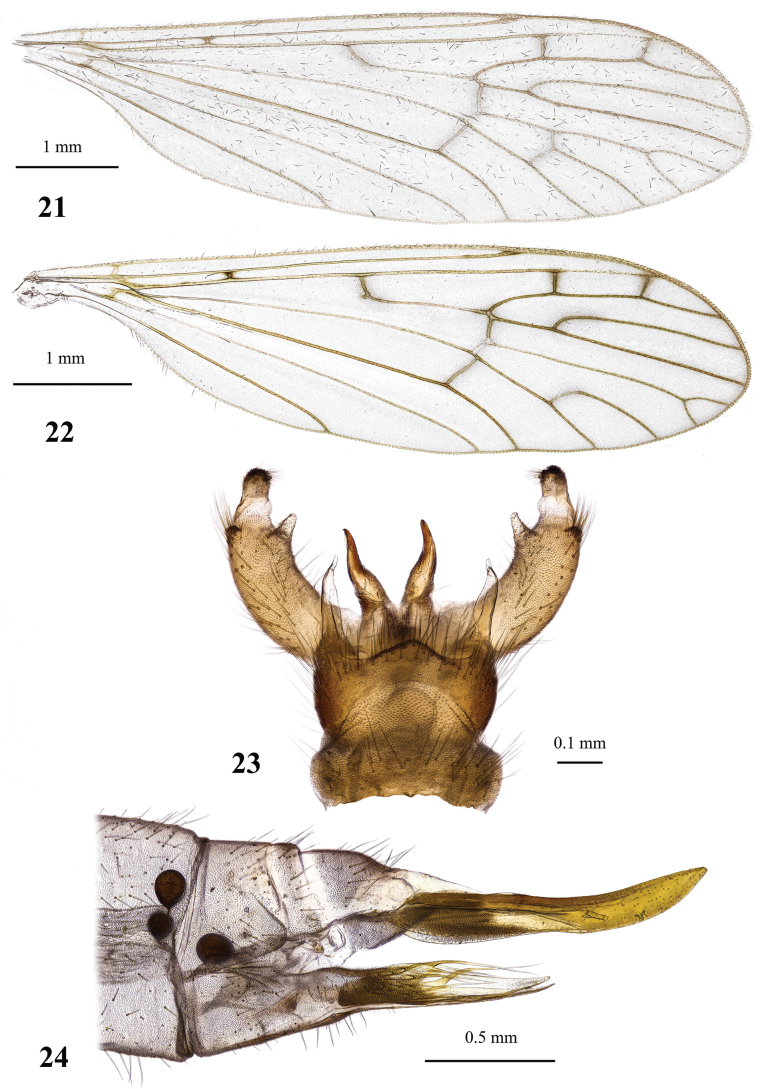
Dicranota (Eudicranota) distincta Podenas, sp. nov., paratypes **21** wing, male **22** wing, variation of venation, female **23** male genitalia, dorsal view **24** ovipositor, lateral view.

Figs 19–20, 21–24, 81

#### Type material

**(Fig. 81). South Korea** • ***Holotype*** ♂ (pinned); Jeollanam-do, Gurye-gun, Toji-myeon, Naeseo-ri, Piagol valley; 35.27177°N, 127.57146°E; alt. 490 m; 24 April 2015 (4); S. Podenas leg.; net; NIBR. ***Paratypes*** • 1 ♀ (in ethanol); Jeollanam-do, Gurye-gun, Toji-myeon, Naeseo-ri, Piagol valley; 35.26586°N, 127.58090°E; alt. 448 m; 27 April 2012 (2); S. Podenas leg.; net; NIBR • 2 ♂ (pinned, genitalia of 1 ♂ in microvial with glycerol on same pin); Jeollanam-do, Gurye-gun, Toji-myeon, Naedong-ri; 35.26137°N, 127.60302°E; alt. 431 m; 29 April 2012 (1); S. Podenas leg.; net; NIBR • 3 ♂, 1 ♀ (in ethanol); Jeollabuk-do, Namwon, Sannae-myeon, Deokdong-ri; 35.33692°N, 127.53230°E; alt. 727 m; 7 May 2013 (5); S. Podenas leg.; net; NIBR • 1 ♀ (in ethanol), 1 ♀ (pinned); Jeollanam-do, Gurye-gun, Toji-myeon, Naeseo-ri, Piagol valley; 35.26580°N, 127.58128°E; alt. 378 m; 10 May 2013; S. Podenas leg.; net; NIBR • 13 ♂, 1 ♀ (in ethanol), 1 ♂ (pinned); Gyeongsangnam-do, Hamyang, Macheon-myeon, Samjeong-ri; 35.35880°N, 127.63672°E; alt. 692 m; 11 May 2013 (2); S. Podenas leg.; net; NIBR • 5 ♂ (in ethanol); Gyeongsangnam-do, Hamyang, Macheon-myeon, Samjeong-ri; 35.34243°N, 127.64102°E; alt. 705 m; 11 May 2013 (4); S. Podenas leg.; net; NIBR • 2 ♂ (pinned), 4 ♂ (in ethanol); Jeollanam-do, Gurye-gun, Toji-myeon, Naeseo-ri, Piagol valley; 35.27177°N, 127.57146°E; alt. 490 m; 24 April 2015 (4); S. Podenas leg.; net; NIBR • 17 ♂, 2 ♀ (pinned); Jeollanam-do, Gurye-gun, Toji-myeon, Naeseo-ri, Piagol valley; 35.27177°N, 127.57146°E; alt. 490 m; 27 April 2015 (2); S. Podenas leg.; net; Genbank No. PQ590791 (215 bp), PQ590792 (398 bp); NIBR • 1 ♀ (in ethanol); Jeollanam-do, Gurye-gun, Toji-myeon, Naeseo-ri, Piagol valley; 35.26590°N, 127.58096°E; alt. 446 m; 28 April 2015 (1); S. Podenas leg.; net; NIBR • 25 ♂ (in ethanol); Jeollanam-do, Gurye-gun, Toji-myeon, Naeseo-ri, Piagol valley; 35.28589°N, 127.55605°E; alt. 773 m; 30 April 2015 (1); S. Podenas leg.; net; NIBR • 1 ♂ (in ethanol); Jeollanam-do, Gurye-gun, Toji-myeon, Naeseo-ri, Piagol valley; 35.27448°N, 127.56378°E; alt. 593 m; 1 May 2015 (1); S. Podenas leg.; net; NIBR • 1 ♂, 2 ♀ (in ethanol, wing of 1 ♀ slide mounted); Jeollanam-do, Gurye-gun, Toji-myeon, Naeseo-ri, Piagol valley; 35.27177°N, 127.57146°E; alt. 490 m; 3 May 2015 (2); S. Podenas leg.; net; NIBR.

#### Other examined material.

**South Korea** • 1 ♀ (pinned); Jeollanam-do, Gurve, Masan-myeon, Hwangjeon-ri; 35.24366°N, 127.48964°E; alt. 101 m; 8 May 2013 (1); S. Podenas leg.; net; NIBR; • 1 ♀ (in ethanol); Gyeongsangnam-do, Sancheong, Sicheon-myeon, Jungsan-ri; 35.30996°N, 127.75163°E; alt. 709 m; 9 May 2013 (1); S. Podenas leg.; net; NIBR.

#### Diagnosis.

Pale yellow species with slightly darkened basal abdominal tergites. Wing translucent, milky with indistinct darker areas surrounding cross-veins, stigma missing. Gonocoxite of male terminalia with narrow subapical cone-shaped lobe on medio-dorsal surface, small subapical setose bump on dorso-lateral surface, small gonostylus and distinct strong darkened horn-shaped paramere.

#### Etymology.

Species is named after that to which it is most closely related, *Dicranota perdistincta*, and because the most reliable character for its discrimination is the distinct horn- or spine-shaped paramere.

#### Description.

General body colouration brownish to pale yellow (Figs 19, 20). Body length of male 5.8 mm, of female ~ 7.3–7.5 mm. Wing length of male 6.1–7.3 mm, of female 6.6–7.1 mm.

***Head*.** Grey dorsally, paler grey along eye margin, yellowish grey posteriorly, obscure yellow ventrally, covered with sparse yellowish setae. Eyes widely separated, distance between them at base of antenna exceeds length of scape in both sexes. Antenna 0.9 mm long in male, reaching slightly beyond frontal margin of prescutum if bent backwards, 1.1 mm in female. Scape yellowish grey, elongate, pedicel oval, yellowish grey, covered with dark brown setae. Flagellum 12-segmented, light grey, slightly darker at distal end, basal flagellomere oval, 2–5 flagellomeres subglobular, remaining segments oval, decreasing in length towards apex, apical flagellomere approximately as long as preceding. Longest verticils slightly exceed length of respective flagellomeres. Rostrum obscure yellow, palpus greyish yellow covered with short dark brown setae. Labellum pale greyish yellow.

***Thorax*.** Pale brownish yellow, covered with sparse greyish pruinosity. Cervical sclerites pale yellow. Pronotum pale, covered with few erect whitish setae. Presutural scutum pale greyish yellow with three indistinct stripes. Stripes not much darker than surrounding area, but they are semi-polished and not covered with pruinosity. Area around stripes covered with bluish grey pruinosity. Tubercular pit missing, pseudosutural fovea indistinct. Prothoracic spiracle surrounded by whitish membrane. Scutal lobe with pale yellow semi-polished area in the middle, margins covered with greyish pruinosity, area between scutal lobes and scutellum whitish. Mediotergite pale. Pleuron pale brownish yellow dorsally, turning totally pale ventrally. Wing (Figs 21, 22) elongate, length/width ratio 3.5, widest slightly before tip of vein *CuP*, milky, iridescent. Stigma missing. Narrow darker brown areas surround *sc-r*, tip of *Sc*, base of *Rs*, *R_2_* and supernumerary cross-vein in cell *r_1_*, cross-veins *r-m* and *m-cu*, distal margin of discal cell. Veins greyish brown to slightly grey and pale at wing base. Venation: *Sc* long, reaching wing margin slightly before level of supernumerary cross-vein in cell *r_1_*, *sc-r* far before the level of *Rs* base or tip of anal vein, approximately at the middle between humeral vein and base of radial sector. *Rs* long, slightly > 4 × as long as cross-vein *m-cu*, usually arcuate at base, but could be also angulate and short spurred. Free end of *R_1_* very short or missing, reaching wing margin together with *R_2_*. Vein *R_2_* nearly transverse, supernumerary cross-vein in cell *r_1_* beyond or at the same level as branching point of *R_4+5_*. *R_3_*, *R_4_*, and *R_5_* nearly parallel to each other. Cell *r_3_* without stem, *R_2+3_* starting slightly before or at same point as *r-m*, cell *r_4_* with short stem, cell itself 3.5–4.6 × as long as its stem. Cross-vein *r-m* distinct, discal cell large, ~ 2.4 × as long as wide, in rare occasions open by atrophy of vein *m-m*. Cell *m_1_* short, its stem ≥ 1.3 × as long as cell itself. Cross-vein *m-cu* at or slightly beyond branching point of *M*. Veins *CuP* and *A_1_* nearly straight just slightly arched before wing margin. Anal angle widely rounded. No brachypterous females were observed. Halter long, its length nearly reaches that of thorax. Length of male halter 1.1–1.2 mm, of female 1.0–1.2 mm. Stem of halter pale with yellowish base, basal half of knob milky, distal part slightly infuscate. Coxae obscure yellow to pale yellow, depending on specimen. Trochanters of fore and middle legs pale greyish yellow, trochanter of hind leg pale or pale yellow. Femora pale greyish yellow with slightly infuscate distal part. Tibiae greyish yellow with widely darkened distal margin. Basitarsi brownish with darker brown distal end and pale base, remaining tarsomeres dark brown. Male femur I: 3.3–3.8 mm long, II: 4.0 mm, III: 4.0–4.4 mm, tibia I: 3.4–3.8 mm, II: 3.2–3.4 mm, III: 3.7–4.1 mm, tarsus I: 4.2–5.2 mm, II: 3.6–3.9 mm, III: 3.8–4.6 mm. Female femur I: 3.3–4.0 mm long, II: 3.8 mm, III: 4.0–4.5 mm, tibia I: 3.0 mm, II: 2.9 mm, III: 2.9–3.8 mm, tarsus I: 3.3 mm, II: 2.8 mm, III: 2.9–3.7 mm. Claw small and simple, without spines.

***Abdomen*.** Obscure yellow. Three basal tergites dark grey medially, margins of darkening vague, lateral margins widely yellow. Remaining tergites and sternites pale yellow, but sclerites are so thin that blackish inner content of guts is visible through and gives dark appearance of the whole abdomen in museum specimens. Abdomen covered with sparse semierect yellowish setae. Male terminalia (Fig. 23) pale yellow, sometimes with weak greyish shade. Posterior margin of epandrium protuberant postero-medially, postero-lateral angle with long blade-shaped lobe, tip of which extended into narrow pale slightly curved caudal lobule. Whole posterior margin of epandrium widely covered with long setae. Gonocoxite elongate, slightly arched, ~ 2.4 × as long as wide, with narrow conical subapical lobe on medio-dorsal surface and small subapical rounded setose bump on dorso-lateral surface. One pair of gonostyli. Gonostylus subglobular with indistinctly protuberant dorsal part (rostrum) covered with long setae. Distal (caudal) part of gonostylus covered with numerous black peg-like spines. Aedeagus short, not visible in dorsal view. Paramere strong, horn-shaped, slightly curved, wider basally, brown to dark brown. Posterior segments of female abdomen generally yellow, concolourous with the rest of abdomen. Tenth tergite pale, cercus greyish yellow with pale yellow distal part, apex acute, slightly raised upwards. Hypogynial valve straight, pale yellow with brownish yellow base. Three spermathecae small, drop-shaped (Fig. 24).

#### Elevation.

From circa 350 m to nearly 800 m.

#### Period of activity.

From late April to middle of May.

#### Habitats.

Larva unknown. Adults fly close to small fast-running springs, small and medium sized mountainous streams running through rocks, covered with mosses or algae. Usually these streams are surrounded by mixed forests or just pine grooves, with sparse grassy vegetation on the ground.

#### Distribution.

Currently known only from South Korea.

#### Remarks.

*Dichranota distincta* Podenas, sp. nov. is most similar and related to *D. perdistincta*. Both species occur in southern Korea. Calculated genetic distance of mt COI between *D. distincta* Podenas, sp. nov. and *D. perdistincta* is > 5%. Presutural scutum of *D. distincta* Podenas, sp. nov. with three indistinct longitudinal stripes, when that of *D. perdistincta* with indistinct medial darkening, lateral stripe missing. Wing of *D. distincta* Podenas, sp. nov. is comparatively narrower with length/width ratio 3.5, when that of *D. perdistincta* is 3.1. Some females of *D. perdistincta* are brachypterous, but we never observed brachypterous females of *D. distincta* Podenas, sp. nov. Cell *m_1_* of *D. distincta* Podenas, sp. nov. is shorter than its stem, distinctly longer, except in brachypterous females, in *D. perdistincta*. Other wing venation characters are variable in both species and cannot be used for species discrimination, for example, *Rs* is usually arcuate in *D. distincta* Podenas, sp. nov. and angulate with short spur in *D. perdistincta*, but some specimens of *D. distincta* Podenas, sp. nov. could be with angulate *Rs* base, and some specimens of *D. perdistincta* arcuate. Despite some differences in body colouration, the most distinct characters for species identification are found in male terminalia. Lobe on meso-dorsal surface of gonocoxite narrow, situated subapically in *D. distincta* Podenas, sp. nov., large and close to the middle in *D. perdistincta*. *D. distincta* Podenas, sp. nov. has small setose subapical bump on dorso-lateral surface, that area is covered with long dense setae, but bump is missing in *D. perdistincta*. Especially distinct is the strong horn-shaped, darkened paramere of *D. distincta* Podenas, sp. nov. while that of *D. perdistincta* is more graceful and terminating in a rod-shaped tip.

### ﻿ Dicranota (Eudicranota) perdistincta

Taxon classificationAnimaliaDipteraPediciidae

Alexander, 1940

BF48623D-0EC6-5879-9C02-2B942F2E35F6

Figs 25–28, 82

Dicranota (Eudicranota) perdistincta Alexander, 1940: 44, figs 4, 29.

#### Type material examined.

**North Korea • *Paratypes***: 1 ♂ (pinned, antenna, hind leg, wing and genitalia slide mounted); Ompo; alt. 152 m; 28 May 1938; A. M. Yankovsky leg.; USNM • 1 ♂ (pinned, antenna, fore leg, wing and genitalia slide mounted); Ompo; alt. 91 m; 29 May 1938; A. M. Yankovsky leg.; USNM • 1 ♂ (pinned); Ompo; alt. 107 m; 8 June 1938; A. M. Yankovsky leg.; USNM 2012845 • ***Allotype*** 1 ♀ (pinned); Ompo; alt. 213 m; 9 June 1938; A. M. Yankovsky leg.; USNM.

#### Other examined material

(Fig. 82). **South Korea** • 2 ♂, 1 ex. (pinned, abdomen broken); #3, 7 miles W of Chungju; [36.97844°N, 127.80099°E]; 27 April 1954; G. W. Byers leg.; U-M, USNM • 3 ♂ (in ethanol); Gangwon-do, Pyeongchang-gun, Daegwallyeong-myeon, Yongsan-ri, Mt. Balwangsan; [37.61458°N, 128.67147°E]; 23 April – 14 May 2008; J. D. Yeo et al. leg.; Malaise trap; NIBR • 144 ♂ (in ethanol), 4 ♂ (pinned); Gangwon-do, Odaesan National Park; 37.81161°N, 128.70116°E; alt. 280 m; 2 May 2012 (2); S. Podenas leg.; among fallen leaves on rock surface; net; Genbank No. PQ590790; NIBR • 22 ♂, 1 ♀ (in ethanol); Jeollabuk-do, Namwon, Unbong-eup, Hwasu-ri; 35.45098°N, 127.57596°E; alt. 509 m; 6 May 2013 (1); S. Podenas leg.; net; NIBR • 13 ♂ (in ethanol); Jeollabuk-do, Namwon, Jucheon-myeon, Gogi-ri; 35.38131°N, 127.48412°E; alt. 450 m; 7 May 2013 (2); S. Podenas leg.; net; NIBR.

#### Redescription.

General body colouration pale yellow. Body length of male 5.7–7.0 mm, of female ~ 5.5 mm. Wing length of male 6.0–7.5 mm, of female 5.0 mm.

***Head*.** Grey dorsally, obscure yellow ventrally, covered with sparse pale setae. Eyes widely separated in both sexes, distance between them at base of antenna exceeds length of both basal antennomeres taken together. Antenna 1.0–1.2 mm long in male, reaching slightly beyond frontal margin of prescutum if bent backwards, pale brown, slightly darker at distal end. Scape brown, pedicel paler brown. Flagellum 10–12-segmented, two or three basal flagellomeres crowded, hardly distinguishable from one another, remaining segments oval. Length of apical flagellomere variable, from smaller to slightly longer than penultimate. Longest verticils ≤ 1.7 × as long as respective flagellomere. Rostrum brownish yellow, palpus pale yellow with slightly infuscate distal palpomere, labellum pale yellow.

**Figures 25–28.**
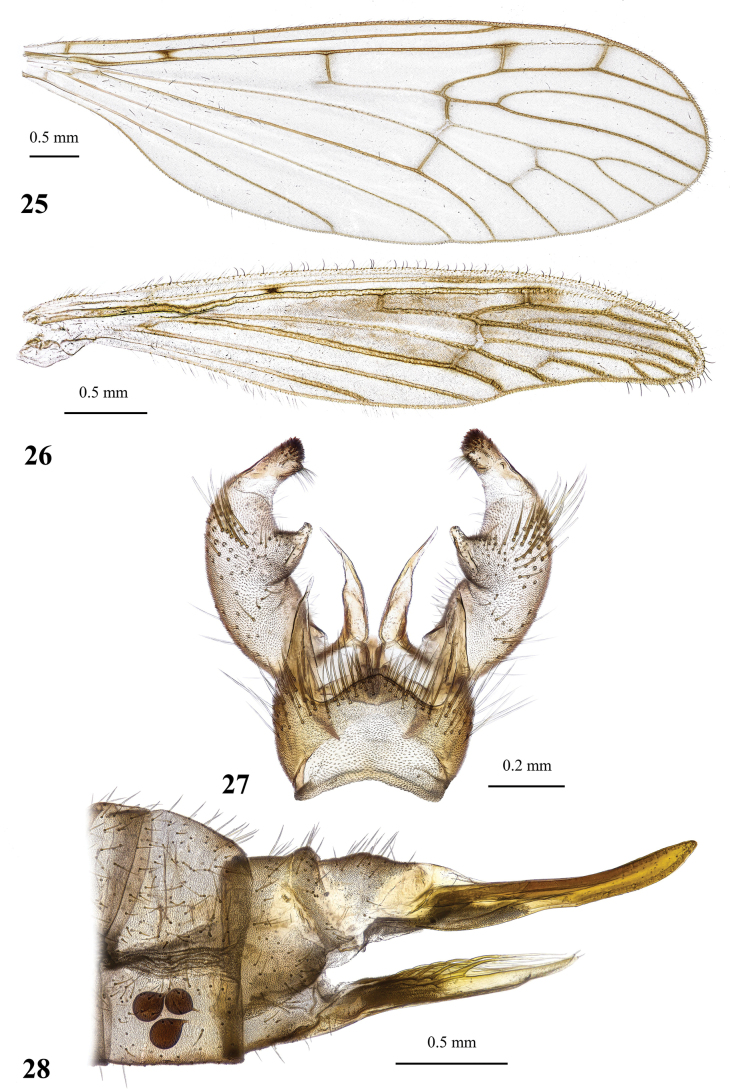
Dicranota (Eudicranota) perdistincta Alexander, 1940 **25** wing, male **26** wing of brachypterous female **27** male genitalia, dorsal view **28** ovipositor, lateral view.

***Thorax*.** Pale to dusky yellow, covered with pale or whitish pruinosity. Pronotum pale brownish yellow covered with setae. Presutural scutum pale yellow widely darker medially, dusted with whitish. Tubercular pit missing, pseudosutural fovea indistinct. Scutal lobe dusky yellow, area between scutal lobes paler. Scutellum pale frontally, obscure yellow posteriorly. Mediotergite pale yellow, brownish yellow caudally. Pleuron uniformly pale yellow, semi-polished where pruinosity has been denuded. Prothoracic spiracle surrounded by whitish membrane. Wing (Fig. 25) comparatively wide, length/width ratio 3.1, widest slightly before tip of vein *CuP*, translucent, strongly iridescent. Stigma indistinct or completely missing. Narrow darker brown areas surround *sc-r*, base of *Rs*, *R_2_* and supernumerary cross-vein in cell *r_1_*, branching point of *Rs* and cross-vein *r-m*, cross-vein *m-cu* surrounded by indistinct darker area. Veins dark brown to pale brown, or pale at wing base. Venation: *Sc* long, reaching level of supernumerary cross-vein in cell *r_1_*, *sc-r* far before the level of *Rs* base or tip of anal vein, distinctly closer to humeral vein than to base of radial sector. *Rs* long, ~ 4 × as long as cross-vein *m-cu*, usually angulate and short spurred, but often arched at base. Free end of *R_1_* very short, nearly missing, much shorter than *R_2_*. Vein *R_2_* nearly transverse, supernumerary cross-vein in cell *r_1_* at the same level as branching point of *R_4+5_*. *R_3_*, *R_4_*, and *R_5_* slightly arched and nearly parallel to each other. Cell *r_3_* without stem, cell *r_4_* with short stem, cell itself ~ 4 × as long as its stem. Cross-vein *r-m* distinct, discal cell large, ~ 2.5 × as long as wide. Cell *m_1_* usually long, ≥ 1.5 × as long as its stem. Cross-vein *m-cu* slightly beyond branching point of *M*. Veins *CuP* and *A_1_* nearly straight just slightly arched before wing margin. Anal angle widely rounded. Usually, there is no difference between male and female wing shape and venation, but in some cases female wing is shorter than that of male when compared with body length, some females are brachypterous with wing strongly reduced and unsuitable for flight (Fig. 26). Such wing is much narrower than normal, length/width ratio nearly reaches 5, venation more or less preserved, but cells darker than in typical form. Halter long, its length exceeds length of thorax. Length of male halter 1.2–1.5 mm, of female 1.1 mm. Stem of halter pale, knob slightly infuscate. Coxae obscure yellow to pale, trochanters pale with narrowly blackened distal rim. Femora pale yellow with pale base and slightly infuscate distal part. Tibiae pale yellow with narrowly dark brown distal margin. Two basal tarsomeres brownish, paler basally, darker distally, remaining tarsomeres brown to dark brown. Male femur I: 3.8–4.1 mm long, II: 3.8–4.2 mm, III: 4.4–4.5 mm, tibia I: 4.1–4.2 mm, II: 3.7–4.3 mm, III: 4.2–4.6 mm, tarsus I: 5.2 mm, II: 4.4–4.7 mm, III: 4.6–4.9 mm. Female femur I: 2.5 mm long, II: 2.2 mm, III: 2.8 mm, tibia I: 2.6 mm, II: 2.5 mm, III: 2.5 mm, tarsus I: 2.7 mm, II: 2.6 mm, III: 3.4 mm. Claw small and simple, without spines.

***Abdomen*.** Dusky yellow to brownish yellow. Tergites with narrowly pale posterior and lateral margins, basal sternite pale yellow, remaining sternites greyish yellow. Abdomen covered with erect pale yellow setae, denser on ventral side. Male terminalia (Fig. 27) concolourous with the rest of abdomen. Posterior margin of epandrium protuberant postero-medially, postero-lateral angle extended into long blade with finger-shaped apex. Whole posterior margin of epandrium covered with long setae. Gonocoxite nearly cylindrical, ~ 2.5 × as long as wide, slightly arched, with large conical outgrowth slightly beyond middle of meso-dorsal surface and densely setose area subapically on dorso-lateral surface. One pair of gonostyli. Indistinct rostral part of gonostylus in nearly dorsal position. Gonostylus truncate, distal part covered with numerous black peg-like spines. Aedeagus short, usually not visible in dorsal view. Paramere long, wider basally, distal part pale, rod-shaped, mesal margin finely setose ~ 1/3–1/2 from base. Posterior segments of female abdomen pale yellow, concolourous with the rest of abdomen. Ovipositor (Fig. 28) yellow. Distal part of cercus just slightly raised upwards, yellow to brownish yellow. Hypogynial valve straight, blackish at base, distal part pale, dorsal margin at basal half with long setae parallel to margin, nearly reaching apex of valve, very tip with short single seta. Spermathecae three, they are small, drop-shaped.

#### Elevation range.

From < 100 m to > 500 m.

#### Period of activity.

From late April through to early July.

#### Habitat.

Larva unknown. Adults fly close to springs, small mountainous streams and rivulets, shaded by mixed forests and shrubs with sparse grassy cover along the margins. Males could be abundant on the ground, crawling among fallen leaves on rocky surfaces in search for females. Some streams with muddy pools alongside.

#### General distribution.

Species is endemic to Korean Peninsula.

#### Remark.

It is the first record of brachypterous female. Species recorded from South Korea for the first time.

### ﻿ Dicranota (Eudicranota) sibiricasibirica

Taxon classificationAnimaliaDipteraPediciidae

(Alexander, 1925)

B84A6809-5AA3-5056-A4A5-D7DB7CDF03B7

Figs 29–33, 83

Rhaphidolabina sibirica Alexander 1925: 10.
Dicranota (Dicranotella) siberica : Alexander 1950: 18 (name only).
Dicranota (Eudicranota) sibirica : Savchenko 1983: 40, fig. 7.
Dicranota (Eudicranota) sibirica sibirica : Savchenko 1989: 20, figs 6.2, 10.1.

#### Type material examined.

**Russia • *Holotype*** (as *Rhaphidolabina siberica*); ♂, wing slide mounted; Siberia, Amagu, Kudia River; July 1923; T. D. A. Cockerell leg.; USNM.

#### Other examined material

**(Fig. 83). North Korea** • 1 ♀ (pinned as Dicranota (Amalopina) sibirica, slide as D. (A.) siberica); (pinned, wing and fore leg slide mounted); Mt. Kongo San; 16 October 1933; G. Machida leg.; C. P. Alexander det.; USNM • 1 ♂ (pinned); Seren Mts.; alt. 762 m; 17 June 1938; A. M. Yankovsky leg.; USNM • 1 ♂ (pinned); Seren Mts.; alt. 1158 m; 10 July 1938; A. M. Yankovsky leg.; USNM • 1 ♂ (pinned); Seren; alt. 853 m; 23 July 1938; A. M. Yankovsky leg.; USNM • 1 ♂ (pinned); Chonsani; alt. 1463 m; 21 June 1940; A. M. Yankovsky leg.; USNM • 1 ♀ (pinned); Chonsani; alt. 1067 m; 29 June 1940; A. M. Yankovsky leg.; USNM • 1 ♀ (pinned); Pontani Paiktusan; alt. 1676 m; 19 July 1940; A. M. Yankovsky leg.; USNM • 1 ♀ (pinned); Pontani Paiktusan; alt. 1372 m; 28 July 1940; A. M. Yankovsky leg.; USNM • 1 ♀ (pinned); Pontani Paiktusan; alt. 1890 m; 5 August 1940; A. M. Yankovsky leg.; USNM • 1 ♀ (pinned); Pontani Paiktusan; alt. 1920 m; 8 August 1940; A. M. Yankovsky leg.; USNM • 2 ♀ (pinned); Pontani Paiktusan; alt. 1942 m; 8 August 1940; A. M. Yankovsky leg.; USNM • 1 ♀ (pinned); Pontani Paiktusan; alt. 1920 m; 10 August 1940; A. M. Yankovsky leg.; USNM • 1 ♀ (pinned); Pontani Paiktusan; alt. 1676 m; 19 August 1940; A. M. Yankovsky leg.; USNM; • 1 ♂ (pinned); Prov. South Phenan, Bong-ha ri; 6–24 July 1982; ForrÓ, Ronkay leg.; HNHM; **South Korea** • 1 ♀ (pinned); #8, Central National Forest, 18 mi. NE Seoul; alt. 122–152 m; 28 May 1954; G. W. Byers leg.; SMEK • 1 ♂ (pinned); #9, Central National Forest, 18 mi. NE Seoul; alt. 122–152 m; 29 May 1954; G. W. Byers leg.; USNM • 1 ♂, 1 ♀ (pinned, ovipositor in microvial with glycerol on same pin); #12, Hwy. #20, 8 mi. SW Kangnung; 37.70000°N, 128.78333°E; alt. 587 m; 8 June 1954; G. W. Byers leg.; SMEK • 1 ♂ (pinned); #37, Hill 1468, 16 mi. NW Chunchon; 38.00000°N, 127.50000°E; alt. 1311 m; 16 September 1954; G. W. Byers leg.; SMEK • 3 ♂ (pinned); #38, Hill 1468, 16 mi. NW Chunchon; 38.00000°N, 127.50000°E; alt. 1311 m; 17 September 1954; G. W. Byers leg.; SMEK • 1 ♂ (in ethanol); Gangwon-do, Pyeongchang-gun, Daegwallyeong-myeon, Yongsan-ri, Mt. Balwangsan; 23 April – 14 May 2008; J. D. Yeo et al. leg.; Malaise trap; NIBR • 2 ♀ (1 ♀ in ethanol, 1 ♀ pinned); Gangwon-do, Pyeongchang-gun, Jinbu-myeon, Dongsan-ri, Odaesan National Park; 37.73920°N, 128.59398°E; alt. 794 m; 22 June 2012 (1); S. Podenas leg.; NIBR • 2 ♂ (in ethanol); Jeollanam-do, Gurye-gun, Toji-myeon, Naeseo-ri, Piagol valley; 35.26580°N, 127.58128°E; alt. 378 m; 10 May 2013; S. Podenas leg.; NIBR • 1 ♂ (in ethanol); Gangwon-do, Inje-gun, Buk-myeon, Hangye-ri, Jayang 3 gyo (bridge), Seoraksan National Park; 38.10415°N, 128.37973°E; alt. 704 m; 7 July 2015 (4); S. Kim, S. Podenas leg; net; NIBR • 1 ♀ (pinned), 1 ♀ (in ethanol); Gyeongsangbuk-do, Gyeongju, Jinhyeon-dong, Tohamsan (Mt.); 35.78755°N, 129.34274°E; alt. 320 m; 27 May 2016 (1); H. M. Baek & S. Podenas leg.; NIBR • 1 ♂ (in ethanol); Gyeongsangbuk-do, Gyeongju, Yangbuk-myeon, Janghang-ri; 35.76236°N, 129.36407°E; alt. 333 m; 28 May 2016 (1); H. Baek, S. Podenas leg.; NIBR • 1 ♂ (pinned); Jeollanam-do, Gurye-gun, Toji-myeon, Naeseo-ri, Piagol valley, 35.26586°N, 127.58090°E, alt. 448 m, 4 June 2016 (2), S. Podenas leg.; NIBR • 1 ♀ (in ethanol); Gyeonggi-do, Pocheon-si, Yeongjung-myeon, Yeongpyeong-ri, MPRC; 38.03644°N, 127.23226°E; alt. 150 m; 24–31 May 2017;T. A. Klein, H.-C. Kim, leg.; New Jersey trap; NIBR • 1 ♀ (pinned); Gyeonggi-do, Gunpo-si, Suri-dong; 37.35022°N, 126.91527°E; alt. 138 m; 27 May 2017 (1); S. Podenas, V. Podeniene leg.; NIBR • 5 ♂, 1 ♀ (pinned, genitalia of 1 ♂ in microvial with glycerol on same pin); Gyeonggi-do, Gunpo-si, Suri-dong; 37.35058°N, 126.91558°E; alt. 138 m; 27 May 2017 (2); S. Podenas leg.; at light; NIBR • 1 ♂ (pinned), 20 ♂, 1 ♀ (in ethanol); Gyeonggi-do, Yangpyeong, Cheongun-myeon, Dowon-ri; 37.54507°N, 127.79483°E; alt. 224 m; 28 May 2017; S. Podenas leg; at light; NIBR • 1 ♂ (in ethanol); Gyeonggi-do, Pocheon-si, Yeongjung-myeon, Yeongpyeong-ri, MPRC; 38.03644°N, 127.23226°E; alt. 150 m; 7 June 2017; T. A. Klein, H.-C. Kim leg.; New Jersey trap; NIBR • 7 ♀ (pinned), 5 ♀ (in ethanol); Gangwon-do, Chuncheon-si, Namsan-myeon, Gongchon-ri; 37.81159°N, 127.64919°E; alt. 131 m; 7 October 2018 (2); S. Podenas leg.; at light; NIBR • 1 ♂, 1 ♀ (in ethanol, male wing slide mounted, genitalia in microvial with glycerol on pin); Gangwon-do, Chuncheon-si, Dongsan-myeon, Kangwon National University Experimental Forest; 37.77909°N, 127.81580°E; alt. 225 m; 9 October 2018; S. Podenas leg.; at light; NIBR • 1 ♀, 1 ex. (in ethanol); Gyeonggi-do, Pocheon-si, Yeongjung-myeon, Yeongpyeong-ri, MPRC; 38.03644°N, 127.23226°E; alt. 150 m; 21 May 2019; T. A. Klein, H. C. Kim leg.; NJ trap; NIBR • 1 ex. (in ethanol); Gyeonggi-do, Paju-si, Jindong-myeon, 1417 Dongpa-ri, Bonifas; 37.92582°N, 126.77410°E; alt. 19 m; 21 May 2019; T. A. Klein, H. C. Kim leg.; NJ trap; NIBR • 1 ex. (in ethanol); Gyeonggi-do, Dongducheon, Tapdong-dong, Casey; 37.87845°N, 127.14566°E; alt. 503 m; 21 May 2019; T. A. Klein, H. C. Kim leg.; NJ trap; NIBR • 1 ex. (in ethanol); Gyeonggi-do, Pocheon-si, Yeongjung-myeon, Yeongpyeong-ri, MPRC; 38.03644°N, 127.23226°E; alt. 150 m; 22 May 2019; T. A. Klein, H. C. Kim leg.; NJ trap; NIBR • 1 ♀ (in ethanol); Gyeonggi-do, Pocheon-si, Yeongjung-myeon, Yeongpyeong-ri, MPRC; 38.03644°N, 127.23226°E; alt. 150 m; 27 May 2019; T. A. Klein, H. C. Kim leg.; NJ trap; NIBR • 1 ♂ (in ethanol); Gyeonggi-do, Pocheon-si, Yeongjung-myeon, Yeongpyeong-ri, MPRC; 38.03644°N, 127.23226°E; alt. 150 m; 4 June 2019; T. A. Klein, H. C. Kim leg.; NJ trap; NIBR • 1 ♀ (in ethanol); Gyeonggi-do, Paju-si, Jinseo-myeon; 37.95433°N, 126.68263°E; alt. 41 m; 29 September 2020; T. A. Klein, H. C. Kim leg.; Green-LED; NNSC-2; NIBR; **Mongolia** • 1 ♂ (wings and genitalia slide mounted); Bulgan Aimag, Khyalgant Soum; 49.62431°N, 104.24514°E; alt. 1015 m; 7–8 July 2005; S. Podenas leg.; SRP#05070702; ANSP; **Russia** • 3 ♀ (pinned); Ussuri Krai, Spassky district, Jakovlevka; 1 October 1926; Filipjev leg.; apiary; 2012845 USNM.

#### Redescription.

General body colouration pale yellow to whitish yellow. Body length of male 5.0–6.9 mm, of female 5.2–8.7 mm. Wing length of male 6.1–7.1 mm, of female 5.4–7.1 mm.

***Head*.** Brown, reddish along posterior margin, densely covered with pale grey pruinosity and sparse pale yellow setae. Antenna short, 0.9 mm long in male, hardly reaching frontal margin of prescutum if bent backwards, 0.7 mm long in female. Scape and pedicel reddish, flagellum 12-segmented, pale yellow with somewhat greyish distal flagellomeres. Basal flagellomeres short, oval, just slightly longer than wider, distal flagellomeres elongate, length of apical segment slightly exceeds penultimate. Longest verticils approximately as long as respective flagellomeres. Rostrum dark brown, densely dusted with grey, palpus pale yellow, labellum pale greyish brown.

**Figures 29–33.**
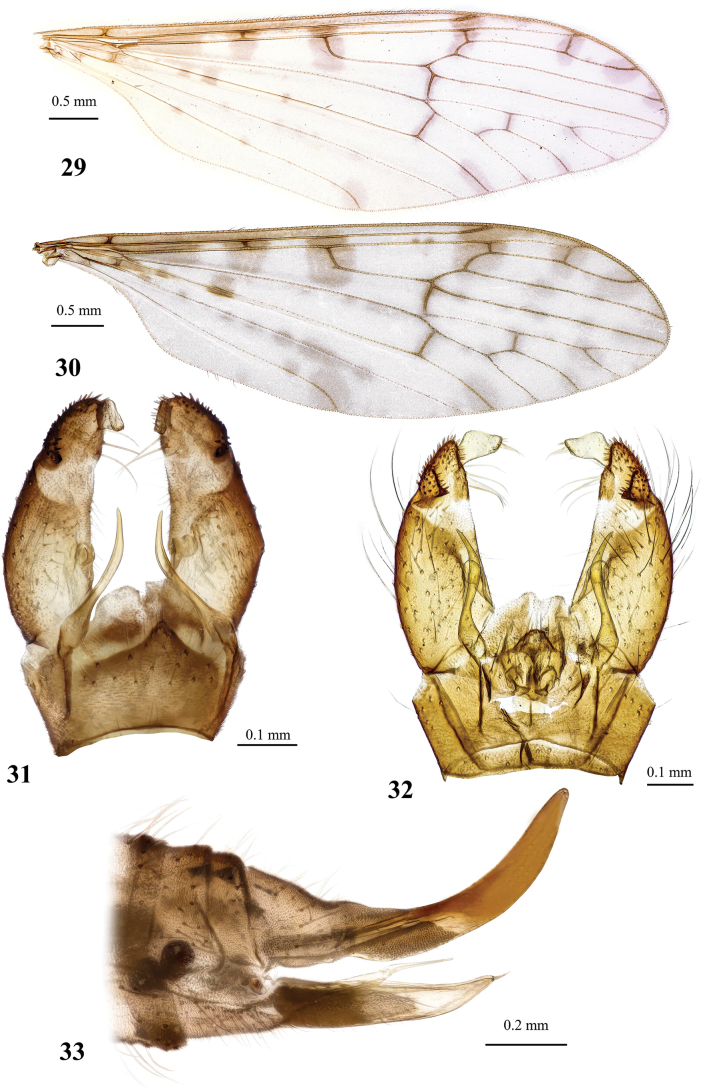
Dicranota (Eudicranota) sibirica sibirica (Alexander, 1925) **29** male wing, holotype **30** female wing **31** male genitalia, dorsal view, in glycerol **32** male genitalia, dorsal view, slide mounted **33** ovipositor, lateral view.

***Thorax*.** Generally pale yellow. Pronotum whitish. Presutural scutum pale yellow without darker stripes. Tubercular pit missing, pseudosutural fovea indistinct. Scutal lobe pale, area between scutal lobes whitish. Scutellum and mediotergite pale. Pleuron yellow to reddish yellow with blurred whitish areas. Membrane surrounding prothoracic spiracle concolourous with rest of pleuron. Wing milky, subhyaline, strongly iridescent, patterned with brown spots, sexually dimorphic. Male wing (Fig. 29) widened posteriorly at tip of anal vein, female wing (Fig. 30) without such widening, widely rounded along posterior margin. Patterning includes completely darkened costal cell from wing base to slightly beyond *Rs* base, subcostal cell also darkened but with clear window before *sc-r*; all cross-veins and branching points surrounded by dark areas, small greyish dots distributed along longitudinal veins, veins *R_4_* and *R_5_* with or without small dots (different from D. (E.) nebulipennis Alexander, 1936 which has at least one large spot in that area). Stigma indistinct. Veins brown or greyish brown, dark brown in darkened areas. Venation: *Sc* long, reaching to or slightly beyond level of supernumerary cross-vein in cell *r_1_*, *sc-r* at ~ 2/3 between humeral vein and base of radial sector. *Rs* long, strongly arched or angulate and short spurred at base. Free end of *R_1_* distinctly shorter than *R_2_*. Vein *R_2_* transverse, supernumerary cross-vein in cell *r_1_* beyond branching point of *R_2+3+4_*. *R_4_* and *R_5_* straight and parallel to each other. Cell *r_3_* with short stem, cross-vein *r-m* distinct, discal cell large, 3.1–3.4 × as long as wide. Cell *m_1_* long, slightly longer than its stem in male, slightly shorter in female. Cross-vein *m-cu* beyond branching point of *M*. Vein *CuP* straight, *A_1_* slightly arched before wing margin. Anal angle widely rounded. Length of male halter 0.9–1.0 mm, of female 1.1 mm. Halter pale greyish, stem pale with whitish base, knob yellowish. Coxae from pale yellow in male to obscure yellow in female, fore coxa darker than posterior, mid-coxa darker at base. Femora whitish with narrowly and indistinctly infuscate apices. Tibiae and basitarsi whitish, second tarsomere slightly darkened distally, remaining tarsomeres brownish. Male femur I: 3.4–4.0 mm long, II: 3.6 mm, III: 3.5–4.8 mm, tibia I: 3.7–4.3 mm, II: 3.7 mm, III: 3.6–4.4 mm, tarsus I: 4.4–4.7 mm, II: 5.0–5.6 mm, III: 4.4–4.6 mm. Female femur I: 3.9 mm long, II: 3.9–4.3 mm, III: 3.4–4.9 mm, tibia I: 4.1 mm, II: 4.0–4.3 mm, III: 3.5–4.4 mm, tarsus I: 5.2 mm, II: 4.7–5.4 mm, III: 3.7–4.7 mm. Claw small and simple, without spines, dark brown.

***Abdomen*.** Tergites weakly bicolorous, pale greyish brown with darker caudal margins, covered with sparse erect greyish setae. Basal sternite pale, second and third sternites pale with darkened posterior margins, remaining sternites greyish brown, paler at base. Sparse setae covering sternites pale. Female abdomen generally paler than that of male. Male terminalia (Figs 31, 32) dark brown. Posterior margin of epandrium extended into triangle-shaped medial lobe, shape of which can vary slightly: narrower in specimens from Korea, wider in specimens from Mongolia. Postero-lateral corner of epandrium with long and narrow, spine-shaped lobe, reaching to ~ 2/3 of gonocoxite. Whole posterior margin of epandrium covered with sparse long setae. Gonocoxite elongate, nearly twice as long as wide, widest at middle. Interbase with narrow stem and ball-shaped tip. Outer gonostylus dark brown and looks like extension of gonocoxite, wide and fleshy, outer and distal margin covered with numerous small blackened spines, mesal margin with three long pale setae. Inner gonostylus elongate, pale, outer margin with low blunt bump at middle, basal part of inner gonostylus encircled by outer gonostylus, tip rounded with few fine setulae. Proctiger divided longitudinally by deep narrow medial incision. Aedeagus short, round-apexed. Paramere long, blade-shaped. Ninth sternite widely concave at middle of posterior margin. Ovipositor (Fig. 33) dark brown with pale distal part of hypogynial valve. Cercus slightly arched, distal part raised upwards, blunt-apexed, hypogynial valve straight, blackish at base, narrowing distally, apex with short strong seta, dorsal margin with three long setae at approx. middle. Spermathecae two, small, subglobular.

#### Elevation range.

From sea level to nearly 2000 m.

#### Period of activity.

From early May through to middle of October.

#### Habitat.

Adults were found among dense grassy vegetation along margins of water bodies and in marshy forest openings in South Primorye (Savchenko 1983), we found them flying along margins of small and medium-sized mountainous streams and rivers densely covered with trees and shrubs, in grassy vegetation surrounding springs and small pools in South Korea. Species is attracted to light.

#### General distribution.

Species was recorded from the eastern Kazakhstan, Honshu Island of Japan, and the Far East of Russia.

#### Remark.

Species recorded from the Korean Peninsula and Mongolia for the first time.

### ﻿ Dicranota (Ludicia)

Taxon classificationAnimaliaDipteraPediciidae

Hutson & Vane-Wright, 1969

510A7F36-1A38-5E33-A41A-50352033EEB8

Pedicia (Ludicia) Hutson & Vane-Wright, 1969: 243.
Rhaphidolabis (Rhaphidolabina) Alexander, 1916: 540; Alexander 1950: 17; Savchenko and Krivolutskaya 1976: 35; Savchenko 1983: 39; Savchenko 1986: 168; Savchenko 1989: 25–26.
Dicranota (Ludicia) Starý 1996: 122.

#### Type species.

*Tricyphona lucidipennis*Edwards 1921 (original designation) (West Palaearctic).

#### Redescription.

Medium-sized dark brown *Dicranota* crane flies.

***Head*.** Antenna 15-segmented, reaching to frontal margin of presutural scutum, if bent backwards. Antennal verticils slightly exceeding length of respective segments.

***Thorax*.** Presutural scutum with four distinct dark longitudinal stripes. Wing without any dark spots except indistinct stigma. *Rs* usually short, sometimes slightly elongate, branches into *R_2+3_* and *R_4+5_*. Discal cell present, but cross-vein *m-m* could be very weak.

***Abdomen*.** Posterior margin of epandrium nearly straight or deeply concave, lateral lobe could be long, narrow and slightly curved or missing. Gonocoxite of male terminalia with dorso-apical lobe. Lobe covered with small black spines. Interbase small. Two pairs of gonostyli. Outer gonostylus covered with small black spines. Ovipositor with long and comparatively wide, slightly arched cercus and straight hypogynial valve reaching to approx. middle of cercus. Dorsal margin of valve covered with long setae at base, tips of them reaching to ~ 2/3 of valve.

The subgenus Dicranota (Ludicia) includes 19 species (Oosterbroek 2025), distributed in the Oriental Region (11 species) and Palaearctic region (8 species), with four species in Eastern and four in Western Palaearctic regions.

### ﻿ Dicranota (Ludicia) emarginata

Taxon classificationAnimaliaDipteraPediciidae

(Alexander, 1945)

A5D968F0-7E4B-5A0A-9AE2-F2981E95E17B

Figs 34–37, 84

Pedicia (Tricyphona) emarginata Alexander, 1945: 243.
Dicranota (Rhaphidolabina) emarginata : Savchenko 1983: 41; Savchenko 1989: 26.
Dicranota (Ludicia) emarginata : Oosterbroek 2025.

#### Type material examined.

**North Korea • *Holotype*** (as Pedicia (Tricyphona) emarginata); ♂ (pinned, antenna, wing, hind leg and genitalia slide mounted); Seren Mountains; alt. 1372 m; 10 July 1938; A. M. Yankovsky leg.; USNM.

#### Other examined material

**(Fig. 84). North Korea** • 3 ♀ (pinned); Ompo; alt. 61 m; 12 May 1938; A. M. Yankovsky leg.; USNM • 5 ♂ (pinned); Ompo; alt. 213 m; 14 May 1938; A. M. Yankovsky leg.; USNM • 1 ♀ (pinned); Ompo; alt. 183 m; 18 May 1938; A. M. Yankovsky leg.; USNM • 1 ♂, 1 ♀ (pinned, male genitalia in microvial with glycerol on same pin); Ompo; alt. 305 m; 19 May 1938; A. M. Yankovsky leg.; USNM • 1 ♂ (pinned, genitalia in microvial with glycerol on same pin); Chonsani; alt. 1219 m; 13 June 1940; A. M. Yankovsky leg.; USNM.

#### Redescription.

General body colouration dark brown with dense cover of silvery grey pruinosity. Body length of male 6.5 mm, of female 5.3–7.2 mm. Wing length of male 7.0–9.5 mm, of female 6.3–6.8 mm.

***Head*.** Brownish grey because of dense pruinosity, sparsely covered with brownish semi-adjacent setae. Anterior vertex wide, eyes comparatively small, widely separated, distance between them ~ 2 × length of scape. Antenna short, 0.9–1.1 mm long in male, reaching to approx. frontal margin of prescutum if bent backwards. Scape and pedicel black, dusted with grey, flagellum of studied non-type specimens and specimens from the Far East of Russia (Savchenko 1983) 13-segmented, but original description mentions that flagellum of holotype male has only 11 segments (Alexander 1945). Re-examination of holotype reveals that it also has 13 segments. Basal flagellomere elongate, > 2 × longer than wide, dark brown with yellowish base, remaining segments dark brown and short, nearly as long as wide (Fig. 35). Apical segment subequal in length to the penultimate. Longest verticils slightly exceed length of respective flagellomeres. Female antenna 0.7–0.9 mm long. Rostrum dark brown, densely dusted with grey, palpus dark brown to black, labellum dark brown.

**Figures 34–37.**
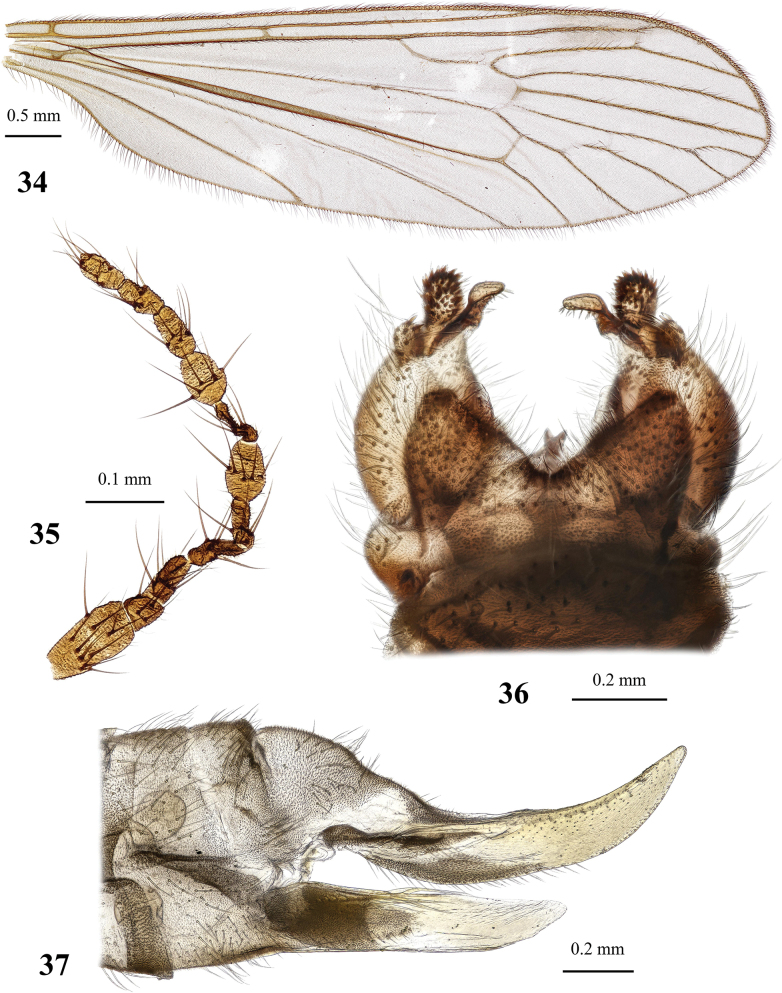
Dicranota (Ludicia) emarginata (Alexander, 1945) **34** wing, holotype **35** antennal flagellum, holotype **36** male genitalia, dorsal view **37** ovipositor, lateral view.

***Thorax*.** Generally dark brown with dense cover of greyish pruinosity. Pronotum brown with dense cover of brownish grey pruinosity and dense yellowish erect setae postero-dorsally. Presutural scutum brownish grey with four dark brown longitudinal stripes. Medial stripes separated by narrow grey vitta for nearly entire length, reaching each other only frontally, stripes not reaching suture posteriorly. Lateral stripe short, starting far beyond pseudosutural fovea frontally, but extending to suture caudally. Tubercular pit missing, pseudosutural fovea indistinct. Scutal lobe dark brown dusted with grey, lateral and mesal margins narrowly grey, area between scutal lobes grey. Scutellum uniformly brownish grey, mediotergite paler frontally. Pleuron dark brown, somewhat paler frontally, densely covered with silvery grey. Membrane surrounding prothoracic spiracle yellowish brown, sparsely dusted with grey. Wing (Fig. 34) subhyaline, brownish with yellowish basal area, iridescent, without darker pattern except elongate darker brown stigma. Veins brown to greyish brown, paler at wing base. Venation: *Sc* long, reaching far beyond branching point of *Rs*, at approx. same level as *m-cu*. Cross-vein *sc-r* nearly at the middle between humeral vein and base of radial sector. *Rs* long, arched at base. Free end of *R_1_* slightly exceeds *R_2_* in length. Vein *R_2_* transverse, distal portion of *R_3_*, *R_4_*, and *R_5_* straight and parallel to each other. Cell *r_3_* sessile. Cell *r_4_* with short but distinct stem *R_4+5_* which is twice as long as cross-vein *r-m*. Discal cell long, nearly 4 × as long as wide, but cross-vein *m-m* very weak in holotype, it is also very weak or completely atrophied in other studied specimens, thus discal cell could be missing. Cell *m_1_* short, stem 1.7 × as long as cell itself. Cross-vein *m-cu* far beyond branching point of *M*, close to the middle of vein *M_3+4_*. Vein *CuP* straight, *A_1_* slightly sinuous. Anal angle widely rounded. Length of male halter 1.2 mm, of female 1.2 mm. Stem of halter obscure yellow, knob pale yellow basally, darkened distally. Fore coxa dark brown with dense cover of grey pruinosity, slightly yellowish along distal margin, middle and posterior coxae dusky yellow with dark base. Femur from pale yellow at base, to obscure yellow at middle, to dark brown at tip. Tibia and tarsal segments dark brown, covered with short yellowish setae. Male femur I: 4.8–5.9 mm long, II: 5.3–5.6 mm, III: 5.0–5.8 mm, tibia I: 5.2–6.4 mm, II: 5.0–5.9 mm, III: 5.2–6.4 mm, tarsus I: 8.1 mm, II: 6.1–7.3 mm, III: 7.1 mm. Female femur I: 3.2–3.5 mm long, II: 3.5 mm, III: 3.8 mm, tibia I: 3.3–3.6 mm, II: 3.5 mm, III: 3.8–4.0 mm, tarsus I: 4.1–5.1 mm, II: 4.0 mm, III: 4.3–4.5 mm. Claw simple, without spines, dark brown.

***Abdomen*.** Basal tergites of male abdomen yellowish brown with dark brown caudal and lateral margins, distal tergites darker brown, caudal margin narrowly light grey. Tergites covered with sparse erect golden setae. Sternites yellowish brown at base of abdomen, turning darker brown towards tip of abdomen. Male terminalia (Fig. 36) dark brown. Posterior margin of epandrium deeply and widely emarginate, lateral lobe wide, setose with rounded posterior margin which is densely covered with fine setulae. Lateral margin of epandrium with narrow lobule, tip of which extends slightly beyond bottom of emargination but far not reaches caudal margin of lateral lobe. Gonocoxite short and wide, length just slightly exceeds width, with two unequal lobes near apex. Caudal lobe dark brown subglobular covered with short black spines, subapical lobe smaller, also blunt-apexed, covered with fine setae. Interbase reduced. Gonostylus pale, triangle-shaped, mesal margin covered with sparse erect setae, tip obtuse, rounded. Aedeagus short, bifid at apex. Paramere long, rod-shaped. Female abdomen uniformly brown with narrowly pale grey caudal margins of tergites and sternites. Caudal segments of female abdomen (9^th^ and 10^th^ tergites, 8^th^ sternite) dark brown. Cercus (Fig. 37) yellow with raised distal part, apex blunt, rounded. Hypogynial valve straight, yellow with brown base and pale rounded tip, dorsal margin with few setae at middle far not reaching apex. Spermathecae three.

#### Elevation range.

From sea level to nearly 1400 m.

#### Period of activity.

From mid-May through to middle of July.

#### Habitat.

Species was collected near small stream running through the hill covered with broad leaved forest in the Far East of Russia (Savchenko 1983).

#### General distribution.

Species was recorded from northern Korea and the Far East of Russia close to the border with North Korea.

#### Remark.

The female was previously unknown. The species is absent in South Korea.

### ﻿ Dicranota (Rhaphidolabis)

Taxon classificationAnimaliaDipteraPediciidae

Osten Sacken, 1869

7A22AE3D-FB62-57CF-8332-8330BE6BAA87

Rhaphidolabis Osten Sacken, 1869: 284; Edwards 1938: 51, 61; Alexander 1950: 17; Ishida 1958: 40; Tjeder 1959: 5; Brindle 1963: 235: Mendl 1972: 150: Savchenko and Krivolutskaya 1976: 35; Savchenko 1983: 39; Savchenko 1986: 170; Savchenko 1989: 27.
Claduroides Brunetti, 1911: 284.

#### Type species.

*Rhaphidolabis tenuipes* Osten Sacken, 1869 (original designation) (Nearctic).

#### Redescription.

Small to medium-sized *Dicranota* crane flies with body length 4.3–9.0 mm and wing length 5.0–9.5 mm. Colour varies from pale yellow to brownish yellow, to pale brown, dark brown, or dark grey.

***Head*.** Antenna 13–17-segmented, reaching to approx. or slightly beyond frontal margin of presutural scutum, if bent backwards. Length of apical antennomere varies depending on species, from shorter to nearly twice as long as preceding segment. Verticils usually short, from half the length to as long as the respective segment, longest reaching ≤ 1.5 × as long as respective segment.

***Thorax*.** Longitudinal stripes of presutural scutum vary depending on species, some species with uniformly coloured scutum and any stripes lacking, some species with medial darkening, and other with three distinct longitudinal stripes. Wing could have small dark spots, surrounding cross-veins or vein branching points, but usually without any darker areas besides stigma, stigma is often missing too. Venation: *Rs* usually short, sometimes longer; cell *r_3_* with short stem, or stem is missing, radial sector branches into *R_2+3+4_* and *R_5_*, or *R_4_* in direct alignment with *Rs*; discal cell open due to atrophy of *m-m*; cell *m_1_* always present. Due to individual variation in wing venation, most species cannot be reliably identified just based on wing characters. Wing squama often with few setae.

***Abdomen*.** Posterior margin of epandrium species specific, often with medial and lateral lobes, sometimes concave at middle. Gonocoxite varies depending on species, sometimes simple, often with subapical dorsal lobe covered with small spines, interbase long, sometimes complicated. Two, sometimes one pair of gonostyli, outer gonostylus fleshy, covered with abundant small black spines, inner gonostylus usually paler, elongate, bearing just few small pale spines. If gonostylus single, then it has complicated structure, usually bearing outer and inner lobes. Aedeagus simple, paramere elongate, usually curved at apex. Ovipositor with long cercus and hypogynial valve. Two or three spermathecae.

The subgenus Dicranota (Rhaphidolabis) includes 112 species (Oosterbroek 2025) (two species are added in this publication). It is most diverse in the Oriental region, 45 species, then Eastern Palaearctic, 37 species (with one species also occurring in the Western Palaearctic, three species shared with Oriental fauna), Nearctic, 31 species. Two species are recorded from Neotropics.

### ﻿ Dicranota (Rhaphidolabis) gibbera

Taxon classificationAnimaliaDipteraPediciidae

(Alexander, 1921)

3BD80680-7730-50AB-8909-8145FCA9B0F8

Figs 38–43, 85

Rhaphidolabina gibbera Alexander, 1921: 121; Alexander 1924: 569.
Dicranota (Amalopina) gibbera karafutonis Alexander, 1930: 520.
Dicranota (Amalopina) gibbera : Savchenko and Krivolutskaya 1976: 35.
Dicranota (Rhaphidolabis) gibbera : Savchenko 1989: 28; Nakamura 2002: 169; Pilipenko and Sidorenko 2006: 264; Kato 2023: 3; Kato and Yamauchi 2023: 4.

#### Type material examined.

**Japan • *Allotype*** (as *Rhaphidolabina gibbera*); ♀ (antenna and wing slide mounted); Gifu; 2 October 1920; K. Takeuchi leg.; USNM; **Russia** (as Japan) ***Paratypes*** (as *Rhaphidolabina gibbera karafutonis*); • 2 ♂ (pinned, wing and genitalia slide mounted); Saghalien, Maoka; 28 July 1922; T. Esaki leg.; USNM.

#### Other examined material

**(Fig. 85). Japan** • 1 ♂ (as *Amalopina gibbera*) (antenna, middle leg, wing and genitalia slide mounted); Kiushiu, Yakushima, Kosuzidani; alt. 457 m; 30 April 1929; S. Issiki leg.; Ch. P. Alexander det.; USNM • 1 ♀ (as Dicranota (Amalopina) gibbera) (wing slide mounted); Kurama, Kyoto; 23 October 1932; Tokunaga leg.; USNM • 1 ♀ (as Dicranota (Rhaphidolabis) gibbera) (wing and leg slide mounted); Shikoku, Mountain Isizuti-Igo; 10 June 1950; Issiki-Ito leg.; Ch. P. Alexander det.; USNM; **Taiwan** (as Formosa) • 2 ♀ (as Dicranota (Amalopina) gibbera) (wing slide mounted); Hassensan; alt. 1372–1829 m; 30 August 1929; S. Issiki leg.; Ch. P. Alexander det.; USNM; **South Korea** • 1 ♀ (pinned); Central National Forest 18 miles NE of Seoul; 26 July 1954; G. W. Byers leg.; USNM • 1 ♀ (in ethanol); Gyeonggi-do, Gapyeong-gun, Buk-myeon, Hwaak-ri; 37.98402°N, 127.52676°E; alt. 579 m; 20 August 2014 (2); S. Podenas & S. Kim leg.; NIBR • 1 ♀ (pinned), 1 ♀ (in ethanol); Jeollanam-do, Gurye-gun, Toji-myeon, Naeseo-ri, Piagol valley; 35.27177°N, 127.57146°E; alt. 490 m; 27 June 2015 (1); S. Podenas leg.; NIBR • 1 ♂ (pinned); Jeollanam-do, Gurye-gun, Toji-myeon, Naeseo-ri, Piagol valley; 35.26590°N, 127.58096°E; alt. 446 m; 27 June 2015 (2); S. Podenas leg.; at light; NIBR • 1 ♂ (pinned), 2 ♀ (in ethanol); Jeollanam-do, Gurye-gun, Toji-myeon, Naeseo-ri, Piagol valley; 35.27448°N, 127.56378°E; alt. 593 m; 1 July 2015 (1); S. Podenas leg.; NIBR • 1 ♀ (in ethanol); Jeollanam-do, Gurye-gun, Toji-myeon, Naeseo-ri, Piagol valley; 35.26586°N, 127.58090°E; alt. 448 m; 2 July 2015 (1); S. Podenas leg.; NIBR • 1 ex. (in ethanol); Jeollanam-do, Gurye-gun, Toji-myeon, Naeseo-ri, Piagol valley; 35.27177°N, 127.57146°E; alt. 490 m; 2 July 2015 (3); S. Podenas leg.; NIBR • 2 ♂, 1 ♀ (in ethanol); Jeollanam-do, Gurye-gun, Toji-myeon, Naeseo-ri, Piagol valley; 35.27177°N, 127.57146°E; alt. 490 m; 3 June 2016 (2); S. Podenas leg.; NIBR • 6 ♂, 1 ♀ (in ethanol); Jeollanam-do, Gurye-gun, Toji-myeon, Naeseo-ri, Piagol valley; 35.26586°N, 127.58090°E; alt. 448 m; 3 June 2016 (4); S. Podenas leg.; at light; NIBR • 1 ♂ (in ethanol); Jeollanam-do, Gurye-gun, Toji-myeon, Naeseo-ri, Piagol valley; 35.27123°N, 127.57133°E; alt. 534 m; 4 June 2016 (1); V. Podeniene leg.; NIBR • 1 ♂ (in ethanol); Jeollanam-do, Gurye-gun, Toji-myeon, Naeseo-ri, Piagol valley; 35.27177°N, 127.57146°E; alt. 490 m; 4 June 2016 (4); S. Podenas leg.; NIBR • 1 ♀ (pinned); Jeollanam-do, Gurye-gun, Toji-myeon, Naeseo-ri, Piagol valley; 35.27123°N, 127.57133°E; alt. 534 m; 4 June 2016 (5); S. Podenas leg.; at light; NIBR • 1 ♂ (pinned, abdomen in microvial with glycerol on same pin); Gyeonggi-do, Yangju-si, Jangheung-myeon, Hoguk-ro; 37.71058°N, 126.98719°E; alt. 157 m; 5 July 2019; S. Podenas leg.; NIBR • 4 ♂, 1 ♀ (in ethanol); Jeju-do, Seogwipo-si, Hawon-dong; 33.34919°N, 126.49536°E; alt. 1230 m; 4 August 2021; J. Kim, C. Lim, D. Lee leg.; net; KUEM • 2 ♂, 1 ♀ (in ethanol); Jeju-do, Seogwipo-si, Hawon-dong; 33.33516°N, 126.47013°E; alt. 990 m; 9 September 2021; J. Kim, D. Lee leg.; net; KUEM • 2 ♂, 4 ♀ (in ethanol); Jeju-do, Seogwipo-si, Namwon-eup, Sillye-ri; 33.33728°N, 126.62075°E; alt. 450 m; 4 August – 9 September 2021; J. Kim, D. Lee leg.; Malaise trap; KUEM • 1 ex. (abdomen broken) (pinned); Jeollabuk-do, Jucheon-myeon, Jinan-gun, Daebul-ri, Site 2; 35.97650°N, 127.40115°E; 22 June 2022; San 195-1; net and light trap; NIBR.

#### Redescription.

General body colouration: head dark brown, thorax yellow, abdomen brown. Body length of male 5.3–6.0 mm, of female 4.3–6.5 mm. Wing length of male 5.0–6.9 mm, of female 5.0–6.3 mm.

**Figures 38–43.**
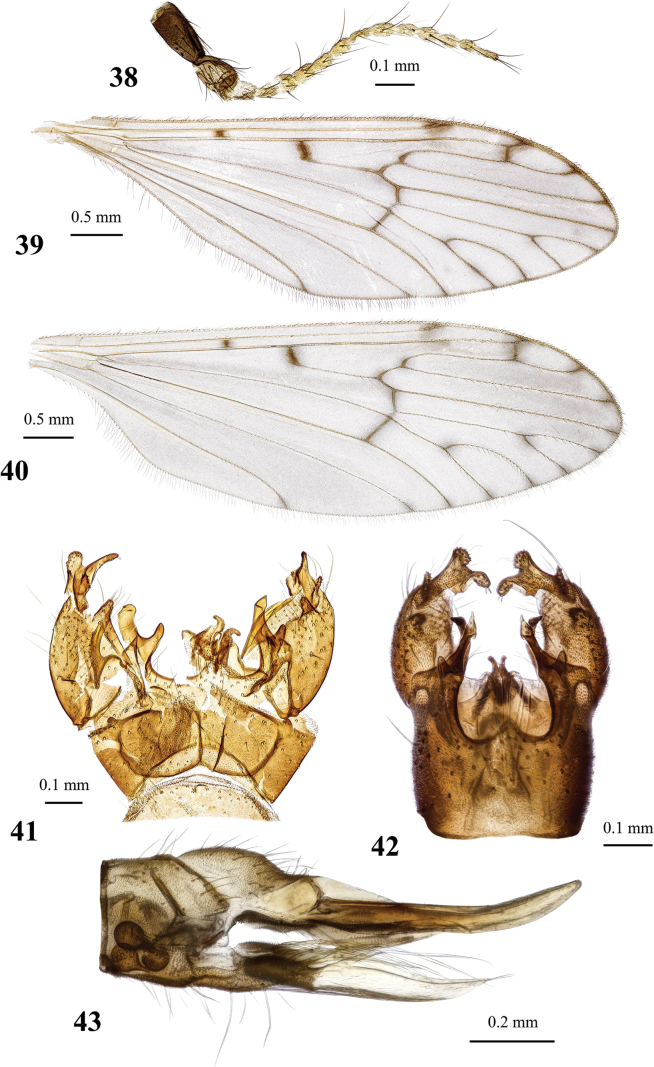
Dicranota (Rhaphidolabis) gibbera (Alexander, 1921) **38** female antenna, allotype **39** male wing, paratype **40** female wing **41** male genitalia, dorsal view, paratype, slide mounted **42** male genitalia, dorsal view, glycerol **43** ovipositor, lateral view.

***Head*.** Dark brown to black covered with sparse grey pruinosity, denser frontally and scattered yellowish setae. Anterior vertex wide, eyes widely separated, distance between them frontally slightly exceeds length of scape. Antenna (Fig. 38) short, 0.7–0.9 mm long in male, 0.7–0.9 mm in female, reaching to approx. frontal margin of prescutum in male if bent backwards. Scape elongate, nearly cylindrical, 1.7 × as long as pedicel, dark brown to black, sparsely dusted with grey and bearing few blackish setae dorsally. Pedicel oval, brown, sparsely dusted with grey. Flagellum 13-segmented, pale yellow with slightly infuscate basal segment. Flagellomeres oval except elongate apical segment, which slightly exceeds penultimate in length. Longest verticils 1.5 × as long as respective flagellomeres. Rostrum dark brown, sparsely dusted with grey, palpus dark brown to black, labellum brown.

***Thorax*.** Generally yellow. Cervical sclerites black. Pronotum black frontally and dorsally, pale yellowish grey laterally, covered with few erect blackish setae dorsally. Presutural scutum pale greyish yellow dorsally, pale laterally with longitudinal brownish medial stripe that does not reach suture posteriorly, some specimens with slightly darker narrow fronto-lateral margin. Stripe wider and more distinct frontally, turns narrower and less distinct caudally. Tubercular pits missing, pseudosutural fovea indistinct. Scutal lobe pale yellow with slightly darker brownish longitudinal line. Area between scutal lobes pale, whitish. Scutellum and mediotergite pale yellow to whitish. Pleuron pale yellow with whitish area extending downwards at base of wing. Membrane surrounding prothoracic spiracle whitish. Wing subhyaline, milky with yellowish costal and basal areas, iridescent. Dark pattern includes narrow but distinct dark spots surrounding all cross-veins except at wing base, branching points and tips of all veins along wing margin, indistinct darker cloud in distal radial field. Stigma with darker margins and clear central area. Veins pale greyish yellow, darkened at branching points and at wing margin. Male wing (Fig. 39) with angulate posterior margin at tip of anal vein, thus anal cell long and narrow, female wing (Fig. 40) with evenly rounded posterior margin, anal cell wider than in male. Venation: *Sc* long, reaching far beyond branching point of *R_2+3+4_*. Cross-vein *sc-r* nearly at the middle between humeral vein and base of radial sector in male, closer to base of *Rs* in female. *Rs* long, angulate at base. Free end of *R_1_* varies from ¼ to as long as *R_2_*. Vein *R_2_* transverse. Distal portion of *R_3_* slightly arched, *R_4_* and *R_5_* straight and parallel to each other. Cell *r_3_* with short stem. Discal cell missing due to reduction of *m-m*. Cell *m_1_* medium-long, its stem ~ 1.5 × as long as cell itself. Cross-vein *m-cu* at or very slightly beyond branching point of *M*. Vein *CuP* very slightly arched, *A_1_* arched before wing margin. Anal angle widely rounded in female, long and narrow in male. Length of male halter 0.9 mm, of female 0.7 mm. Stem of halter grey with yellow base, knob with pale base, yellow central area and dark grey distal margin. Coxae pale yellow, just fore coxa slightly darkened frontally. Trochanters pale with narrowly blackened ventral margin. Femur of fore and middle legs entirely black, that of posterior leg pale yellow with narrowly darkened tip. All tibiae and basitarsi snow white with indistinctly darker tips. Second tarsomere whitish with darker distal part, third tarsomere whitish at basal half, greyish at distal, two remaining tarsomeres grey. Male femur I: 3.1 mm long, II: 3.7 mm, III: 3.2–3.8 mm, tibia I: 2.9 mm, II: 3.2–3.6 mm, III: 2.8–3.4 mm, tarsus I: 3.0 mm, II: 3.1–3.4 mm, III: 2.7–3.4 mm. Female femur I: 3.1 mm long, II: 3.1–3.7 mm, III: 3.5–3.9 mm, tibia I: 2.9 mm, II: 2.9–3.4 mm, III: 3.0–3.6 mm, tarsus I: 3.2 mm, II: 2.6–2.9 mm, III: 3.1–3.2 mm. Claw simple, without spines, greyish.

***Abdomen*.** Abdominal segments semi-polished. Basal tergite greyish, second and third tergites pale yellow with darkened distal margin, remaining tergites dark grey. Basal sternite pale yellow with darker distal margin, succeeding four sternites pale yellowish grey, pregenital sternites dark grey. Male terminalia (Figs 41, 42) dark grey with yellowish epandrium. Posterior margin of epandrium with small round-apexed bump at middle covered with long setae, postero-lateral angle extended into long and wide complicated lobe, bearing two branches: dorsal branch simple, extended, finger shaped in dorsal view, spine-shaped in lateral; ventral lobe distinctly longer, terminating in wider head, armed with two spines directed opposite to each other. Gonocoxite elongate with finger-shaped lobule dorso-apically. Interbase slightly arched spine-shaped, extending slightly beyond lobes of epandrium. One pair of complicated gonostyli. Gonostylus with narrow finger-shaped (in dorsal view) dorso-basal lobe that is wide and flat in lateral view, distal margin of which covered with small blackish spinules; ventral part resembles small head with long rostrum, head covered with small blackish spinules too, rostral appendage terminates in two strong setae (rostral appendage extends dorso-mesally thus looks rather short in dorsal view Fig. 42). Distal part of proctiger divided longitudinally by shallow medial incision. Aedeagus short and narrow. Paramere long and narrow, distinctly arched. Ovipositor (Fig. 43) pale brown. Cercus brown, distal part slightly raised upwards, pale, blunt apexed. Hypogynial valve nearly straight, darker at base, apical part pale, dorsal margin basally with long setae reaching apex of valve, very tip with short single seta. Three small drop-shaped spermathecae.

#### Elevation range.

From circa 150 m to 1400 m (probably up to 1800 m in Taiwan).

#### Period of activity.

From late April through to late October.

#### Habitat.

Adults fly along margins of small and medium-sized mountainous rivers and streams running through forested areas, above moss covered rocks and debris accumulated along margins on the slopes, wet areas with water seeping through rock surface, boggy areas surrounded by mixed forest. Males swarm under tree canopies before sunset. Both sexes are attracted to light.

#### General distribution.

Japan, Far East of Russia, South Korea, Taiwan.

#### Remark.

Species recorded from the Korean Peninsula for the first time.

### ﻿ Dicranota (Rhaphidolabis) luteola

Taxon classificationAnimaliaDipteraPediciidae

Alexander, 1938

ADDAF999-BA64-5354-A805-56BA6C03D2E9

Figs 44–48, 86

Dicranota (Rhaphidolabis) luteola Alexander, 1938b: 152; Savchenko and Krivolutskaya 1976: 38–39 (misidentification); Savchenko 1983: 42–43; Savchenko 1989: 28; Pilipenko and Sidorenko 2006: 140; Pilipenko 2009: 335.

#### Type material examined.

**North Korea • *Holotype*** ♀ (antenna and wing slide mounted); Ompo; alt. 46 m; 7 June 1937; A. M. Yankovsky leg.; USNM; ***Paratype*** • 1 ♀ (wing slide mounted); Ompo; 7 June 1938 (probably, should be 1937, because original description says that it is paratopotypical); A. M. Yankovsky leg.; USNM.

#### Other examined material

**(Fig. 86). North Korea** • 1 ♀ (pinned); Ompo; alt. 183 m; 22 May 1938; A.M. Yankovsky leg.; USNM • 1 ♀ (pinned); Ompo; alt. 46 m; 25 May 1938; A.M. Yankovsky leg.; USNM • 1 ♂, 4 ♀ (pinned, male genitalia in microvial with glycerol on same pin); Ompo; alt. 91–152 m; 29 May 1938; A.M. Yankovsky leg.; USNM • 2 ♀, 1 ex. (pinned); Ompo; alt. 107 m; 8 June 1938; A.M. Yankovsky leg.; USNM • 1 ♂ (pinned, genitalia in microvial with glycerol on same pin); Seren; alt. 914 m; 14 June 1938; A.M. Yankovsky leg.; USNM • 2 ♂, 2 ex. with broken abdomens (pinned, 1 male genitalia in microvial with glycerol on same pin); Seren; alt. 762 m; 18 June 1938; A.M. Yankovsky leg.; USNM • 4 ♂, 1 ♀ (pinned, 1 ♂ genitalia in microvial with glycerol on same pin); Seren; alt. 1280 m; 18 June 1938; A.M. Yankovsky leg.; USNM • 5 ♂ (pinned, genitalia in microvials with glycerol on same pins); Seren; alt. 914 m; 22 June 1938; A.M. Yankovsky leg.; USNM • 1 ♀ (pinned); Seren; alt. 1219 m; 22 June 1938; A.M. Yankovsky leg.; USNM • 1 ♀, 1 ex. with broken abdomen (pinned); Seren; alt. 549 m; 26 June 1938; A.M. Yankovsky leg.; USNM • 2 ♂, 1 ex. with broken abdomen (pinned, 1 ♂ genitalia in microvial with glycerol on same pin); Seren; alt. 762 m; 26 June 1938; A.M. Yankovsky leg.; USNM • 3 ♀ (pinned); Seren; alt. 1067 m; 29–30 June 1938; A.M. Yankovsky leg.; USNM • 3 ♂, 2 ♀ (pinned); Seren; alt. 853 m; 2–3 July 1938; A.M. Yankovsky leg.; USNM • 2 ♀ (pinned); Seren; alt. 914 m; 5–6 July 1938; A.M. Yankovsky leg.; USNM • 3 ♀ (pinned); Seren; alt. 1219 m; 18–19 July 1938; A.M. Yankovsky leg.; USNM • 2 ♂, 2 ♀ (pinned, male genitalia in microvials with glycerol on same pins); Seren; alt. 1372 m; 18–19 July 1938; A.M. Yankovsky leg.; USNM • 1 ♂ (antenna, fore, middle and hind legs, wing and abdomen slide mounted); Kankyo Nando, Puksu Pyaksan; alt. 1768 m; 13 June 1939; A. M. Yankovsky leg.; Ch. P. Alexander det.; USNM • 1 ♂ (antenna, leg, wing and genitalia slide mounted); Kankyo Nando, Puksu Pyaksan; alt. 1524 m; 24 July 1939; A. M. Yankovsky leg.; Ch. P. Alexander det.; USNM • 1 ♂ (pinned, genitalia in microvial with glycerol on same pin); Chonsani; alt. 1219 m; 8 June 1940; A.M. Yankovsky leg.; USNM; **South Korea** • 1 ♂, 1 ♀ (in ethanol); Gangwon-do, Pyeongchang-gun, Jinbu-myeon, Dongsan-ri, Odaesan National Park; 37.73920°N, 128.59398°E; alt. 794 m; 22 June 2012 (1); S. Podenas leg.; NIBR • 2 ♂, 3 ♀ (in ethanol); Gyeongsangbuk-do, Yeongju-si, Punggi-eup, Sucheol-ri; 36.91772°N, 128.45811°E; alt. 700 m; 4 October 2019; C. Lim, C. V. Duong leg.; net; KUEM • 1 ♂, 1 ♀ (in ethanol); Gyeongsangbuk-do, Hamyang-gun, Macheon-myeon, Samjeong-ri; 35.34214°N, 127.64049°E; alt. 740 m; 30 September 2021; J. Kim, C. Lim, D. Lee leg.; net; KUEM.

#### Redescription.

General body colouration yellow to brownish yellow. Body length of male ~ 4.5 mm, of female 5.2–6.0 mm. Wing length of male 5.8–6.0 mm, of female 5.3–7.0 mm.

**Figures 44–48.**
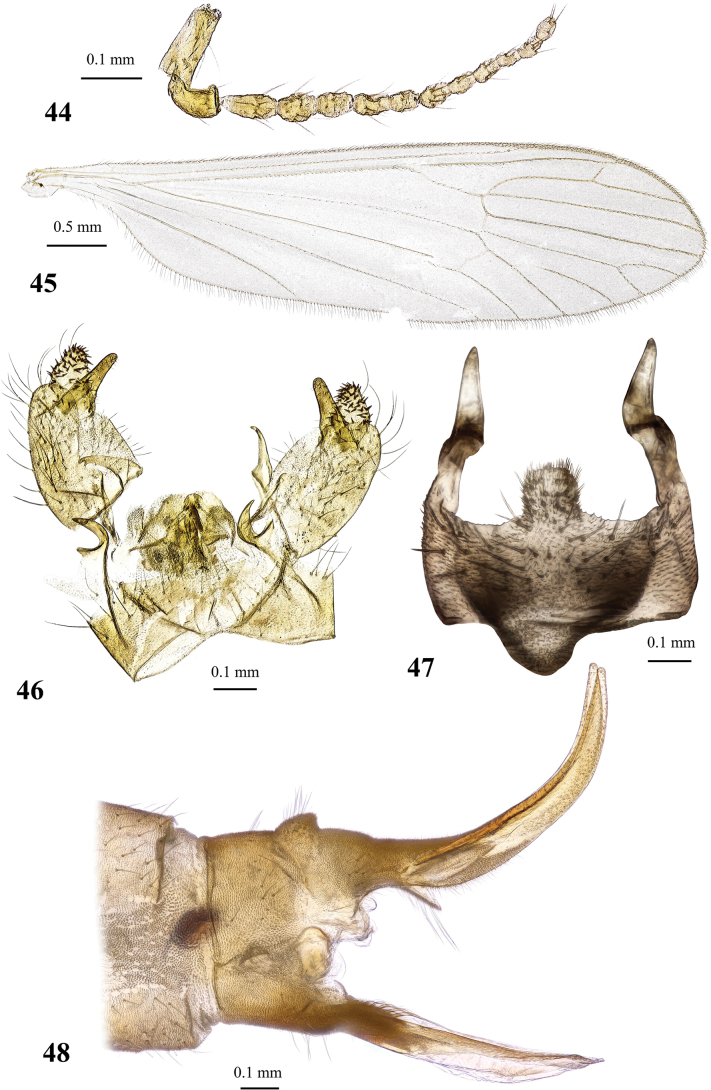
Dicranota (Rhaphidolabis) luteola Alexander, 1938 **44** female antenna, holotype **45** male wing **46** male genitalia, dorsal view **47** epandrium, dorsal view **48** ovipositor, lateral view.

***Head*.** Brownish grey to light grey because of dense grey pruinosity. Antenna (Fig. 44) short and thick, hardly reaching frontal margin of prescutum if bent backwards in male, 0.6–0.8 mm long in female. Scape yellow to yellowish brown. Pedicel yellow in male, darker in female. Flagellum 11-segmented, dark brown or black. Flagellomeres compact, oval or barrel-shaped, covered with pale pubescence. Apical flagellomere large, at least as long as preceding segment. Longest verticils slightly shorter than respective flagellomeres. Rostrum yellow to brownish yellow, two basal palpomeres yellow, two terminal palpomeres black.

***Thorax*.** Generally yellow, dorsum yellowish brown dusted with grey. Presutural scutum pale brown without longitudinal stripes, sometimes with dark medial darkening. Scutellum, mediotergite and pleuron yellow. Wing (Fig. 45) long and narrow both in male and female, nearly 4 × as long as wide, subhyaline, milky, slightly iridescent, without darker pattern, except cells beyond cord which are a trifle darker. Stigma missing. Veins pale brownish grey, a little darker beyond cord. Venation: *Sc* long, reaching far beyond branching point of *R_2+3+4_*. Cross-vein *sc-r* could be closer to base of *Rs* than to humeral vein, in the middle between base of *Rs* and humeral vein in holotype, it could be very weak or totally missing in some specimens. *Rs* ~2 × as long as *m-cu*, nearly straight, just slightly arched at base. Free end of *R_1_* very short, distinctly shorter than *R_2_*. Vein *R_2_* oblique. Distal portions of *R_3_*, *R_4_*, and *R_5_* straight and parallel to each other. Cell *r_3_* with very short stem, distinctly shorter than *r-m*, but length of stem varies individually. Discal cell missing due to reduction of *m-m*, but Savchenko (1983) mentions, that some specimens, both males and females have fully developed or reduced vein *m-m*. Cell *m_1_* short, its stem 2.6 × as long as cell itself. Cross-vein *m-cu* distinctly beyond branching point of *M*. Vein *CuP* nearly straight, *A_1_* slightly arched. Anal angle widely rounded. Halter pale yellow with more intensely yellow knob. Length of male halter 0.8 mm, of female 0.9 mm. Coxae and trochanters yellow to pale yellow. Femora pale brown. Tibiae and basitarsi whitish to pale yellow with narrowly darkened tips. Second and third tarsomeres whitish yellow, terminal tarsomeres darkened. Male femur I: 3.8 mm long, II: 3.9 mm, III: 4.3 mm, tibia I: 4.6 mm, II: 3.8–3.9 mm, III: 3.8 mm, tarsus I: 5.5 mm, II: 4.3–4.4 mm, III: 3.8 mm. Female femur I: 3.7 mm long, II: 3.2–4.2 mm, III: 3.6–4.1 mm, tibia I: 3.4 mm, II: 3.1–3.8 mm, III: 3.4–4.1 mm, tarsus II: 3.0–4.5 mm, III: 3.7–4.4 mm. Claw simple, without spines, pale yellow.

***Abdomen*.** Yellow to brownish yellow in male, somewhat darker in female. Tergites a little more infuscated than sternites, especially medially. Entire sixth segment dark brown in male, only posterior margin darkened in female. Seventh segment dark brown both in male and female. Male terminalia (Fig. 46) yellow. Posterior margin of epandrium nearly straight with distinct nearly parallel-sided medial plate and sickle-shaped lateral lobe (Fig. 47). Medial lobe nearly quadrangular, approximately as long as wide. Gonocoxite slightly longer than wider, simple, without additional lobes. Interbase long, wider at base, slightly arched, bifid at tip. Outer gonostylus short and wide, round-apexed, covered with dense blackish spinulae. Inner gonostylus long and narrow, finger-shaped. Aedeagus short and wide. Ninth sternite with very deep and wide membranous concavity postero-medially. Ovipositor (Fig. 48) brownish yellow. Cercus polished brown basally, brownish yellow distally.

#### Elevation range.

From sea level to nearly 1800 m.

#### Period of activity.

Adults fly the whole of both June and July.

#### Habitats.

Small mountainous streams, partly disappearing underground, densely covered with broad leaved trees, shrubs, herbaceous vegetation in Korea. Southern slopes of rocky canyons of small streams surrounded by broad leaved forests in the Kedrovaya Padj sanctuary in the Far East of Russia (Savchenko 1983).

#### General distribution.

North Korea and Far East of Russia.

#### Remarks.

The species is recorded from South Korea for the first time. *Dicranota luteola* is similar to *D. complicata* Savchenko, 1979 from Sakhalin. *Dicranota complicata* was mistakenly identified as *D. luteola* by Savchenko and Krivolutskaya (1976), later described as new species. Judging from the illustration in Savchenko (1983: fig. 10) males from the Far East of Russia (Kedrovaya Padj sanctuary) and males from Korea identified as *D. luteola* by Ch. P. Alexander belong to different species. Especially, distinct differences exist in the structure of epandrium: the medial lobe of Korean males is much shorter than that of Savchenko’s specimens, the lateral lobe of Korean males is distinctly larger and stronger. The interbase of Korean males has a subapical tooth resembling that of *D. complicata*, but the interbase of Savchenko’s males has no subapical tooth. The original description of *D. luteola* was based on two females. Probably, at least three related species exist in the Eastern Palaearctic, and Savchenko’s males from the Far East of Russia belong to new undescribed species.

### ﻿ Dicranota (Rhaphidolabis) minuscula

Taxon classificationAnimaliaDipteraPediciidae

Alexander, 1938

70315E3B-B5C1-5F16-AF0C-4817F7907895

Figs 49–52, 87

Dicranota (Rhaphidolabis) flavibasis minuscula Alexander, 1938b: 151; Savchenko and Krivolutskaya 1976: 42–43; Savchenko 1983: 44–45; Savchenko 1989: 28.
Dicranota (Rhaphidolabis) minuscula : Oosterbroek 2025.

#### Type material examined.

**North Korea • *Holotype*** (as Dicranota (Rhaphidolabis) flavibasis minuscula) ♂ (antenna, hind leg, wing and genitalia slide mounted); Ompo; alt. 244 m; 22 September 1937; A. M. Yankovsky leg.; USNM.

#### Other examined material

**(Fig. 87). North Korea** • 1 ♀ (pinned); Ompo; alt. 76 m; 7 May 1938; A. M. Yankovsky leg.; USNM • 1 ♀ (pinned); Chonsani; alt. 1067 m; 29 June 1940; A. M. Yankovsky leg.; USNM • 1 ♀ (pinned); Chonsani; alt. 1219 m; 29 June 1940; A. M. Yankovsky leg.; USNM • 1 ♀ (pinned); Chonsani; alt. 1219 m; 4 July 1940; A. M. Yankovsky leg.; USNM • 1 ♀ (pinned); Chonsani; alt. 1372 m; 7 July 1940; A. M. Yankovsky leg.; USNM • 2 ♀ (pinned, ovipositor of 1 ♀ in microvial with glycerol on same pin); Pontani Paiktusan; alt. 1829 m; 2 August 1940; A. M. Yankovsky leg.; USNM • 1 ♀ (pinned); Pontani Paiktusan; alt. 1676–1890 m; 4 August 1940; A. M. Yankovsky leg.; USNM • 2 ♀ (pinned); Pontani Paiktusan; alt. 1768–1942 m; 8 August 1940; A. M. Yankovsky leg.; USNM • 1 ♀ (pinned); Pontani Paiktusan; alt. 1920 m; 8 August 1940; A. M. Yankovsky leg.; USNM • 1 ♀ (pinned); Pontani Paiktusan; alt. 1768 m; 13 August 1940; A. M. Yankovsky leg.; USNM • 3 ♀ (pinned); Pontani Paiktusan; alt. 1920 m; 20 August 1940; A. M. Yankovsky leg.; USNM • 2 ♀ (pinned); Pontani Paiktusan; alt. (1829?)–1942 m; 23 August 1940; A. M. Yankovsky leg.; USNM • 1 ♀ (pinned); Pontani Paiktusan; alt. 1829–1942 m; 25 August 1940; A. M. Yankovsky leg.; USNM; **South Korea** • 1 ♂, 1 ♀ (in ethanol); Jeollanam-do, Gurye-gun, Toji-myeon, Naeseo-ri, Piagol valley; 35.26586°N, 127.58090°E; alt. 448 m; 27 April 2012 (2); S. Podenas leg.; NIBR • 3 ♂, 1 ♀ (in ethanol); Gyeongsangnam-do province, Samjeong village; 35.30246°N, 127.63439°E; alt. 640 m; 28 April 2012; S. Podenas leg.; NIBR • 3 ♂ (pinned, genitalia in microvials with glycerol on same pins); Jeollabuk-do, Namwon, Sannae-myeon, Deokdong-ri; 35.33629°N, 127.53230°E; alt. 727 m; 7 May 2013 (5); S. Podenas leg.; NIBR • 1 ♀ (pinned); Jeollanam-do, Gurye-gun, Masan-myeon, Hwangjeon-ri; 35.24366°N, 127.48964°E; alt. 101 m; 8 May 2013 (1); S. Podenas leg.; NIBR • 1 ♀ (pinned); Gyeongsangnam-do, Hadong-gun, Hwagae-myeon, Beomwang-ri; 35.27655°N, 127.61796°E; alt. 364 m; 8 May 2013 (3); S. Podenas leg.; NIBR • 1 ♂ (pinned, genitalia in microvial with glycerol on same pin); Gyeongsangnam-do, Sancheong, Sicheon-myeon, Jungsan-ri; 35.30996°N, 127.75163°E; alt. 709 m; 9 May 2013 (1); S. Podenas leg.; NIBR • 1 ♀ (pinned); Jeollanam-do, Gurye-gun, Toji-myeon, Naeseo-ri, Piagol valley; 35.26580°N, 127.58128°E; alt. 378 m; 10 May 2013; S. Podenas leg.; NIBR • 1 ♀ (pinned), 1 ♂, 1 ♀ (in ethanol); Gyeongsangnam-do, Hamyang, Macheon-myeon, Samjeong-ri; 35.34243°N, 127.64102°E; alt. 705 m; 11 May 2013 (4); S. Podenas leg.; NIBR • 1 ♂, 1 ♀ (pinned, male genitalia in microvial with glycerol on same pin); Jeollanam-do, Gurye-gun, Toji-myeon, Naeseo-ri, Piagol valley; 35.27177°N, 127.57146°E; alt. 490 m; 24 April 2015 (4); S. Podenas leg.; NIBR • 1 ♂ (in ethanol); Jeollanam-do, Gurye-gun, Toji-myeon, Naeseo-ri, Piagol valley; 35.26590°N, 127.58096°E; alt. 446 m; 25 April 2015 (2); S. Podenas leg.; NIBR • 1 ♂, 1 ♀ (pinned, male genitalia in microvial with glycerol on same pin); Jeollanam-do, Gurye-gun, Toji-myeon, Naeseo-ri, Piagol valley; 35.26590°N, 127.58096°E; alt. 446 m; 26 April 2015 (1); S. Podenas leg.; NIBR • 1 ♀ (pinned); Jeollanam-do, Gurye-gun, Toji-myeon, Naeseo-ri, Piagol valley; 35.25825°N, 127.58208°E; alt. 310 m; 26 April 2015 (2); S. Podenas leg.; NIBR • 2 ♀ (pinned); Jeollanam-do, Gurye-gun, Toji-myeon, Naeseo-ri, Piagol valley; 35.26590°N, 127.58096°E; alt. 446 m; 27 April 2015 (1); S. Podenas leg.; NIBR • 1 ♂, 6 ♀ (pinned, male genitalia in microvial with glycerol on same pin); Jeollanam-do, Gurye-gun, Toji-myeon, Naeseo-ri, Piagol valley; 35.27177°N, 127.57146°E; alt. 490 m; 27 April 2015 (2); S. Podenas leg.; NIBR • 3 ♀ (in ethanol); Jeollanam-do, Gurye-gun, Toji-myeon, Naeseo-ri, Piagol valley; 35.28589°N, 127.55605°E; alt. 773 m; 30 April 2015 (1); S. Podenas leg.; NIBR • 3 ♀ (in ethanol); Jeollanam-do, Gurye-gun, Toji-myeon, Naeseo-ri, Piagol valley; 35.27448°N, 127.56378°E; alt. 593 m; 1 May 2015 (1); S. Podenas leg.; NIBR • 1 ♂, 1 ♀ (in ethanol); Gangwon-do, Gapyeong-gun, Buk-myeon, Jeokmok-ri; 37.97627°N, 127.44160°E; 27 September 2015; Y. Bae leg.; KUEM • 1 ♀ (pinned); Gangwon-do, Chuncheon-si, Namsan-myeon, Gongchon-ri; 37.81159°N, 127.64919°E; alt. 131 m; 7 October 2018 (2); S. Podenas leg.; NIBR • 16 ♀ (in ethanol); Gangwon-do, Chuncheon-si, Dongsan-myeon, Kangwon National University Experimental Forest; 37.77909°N, 127.81580°E; alt. 225 m; 9 October 2018; S. Podenas leg.; at light and with net; NIBR • 3 ♀ (pinned); Doiryung Valley, Bukhansan National Park; 37.69037°N, 126.98972°E; alt. 242 m; 16 October 2018 (2); H.-Y. Seo, S. Podenas leg.; NIBR • 1 ♀ (pinned, wings slide mounted); Gyeonggi-do, Yangju-si, Jangheung-myeon, Uldae-ri; 37.74258°, 127.00329°E; alt. 162 m; 19 October 2018; A. Petrasiunas leg.; stream margin; NRC.

#### Redescription.

General body colouration greyish dark brown with denser grey pruinosity covering thorax. Body length of male ~ 6.5 mm, of female 6.2–8.8 mm. Wing length of male 7.2 mm, of female 6.3–8.3 mm.

**Figures 49–52.**
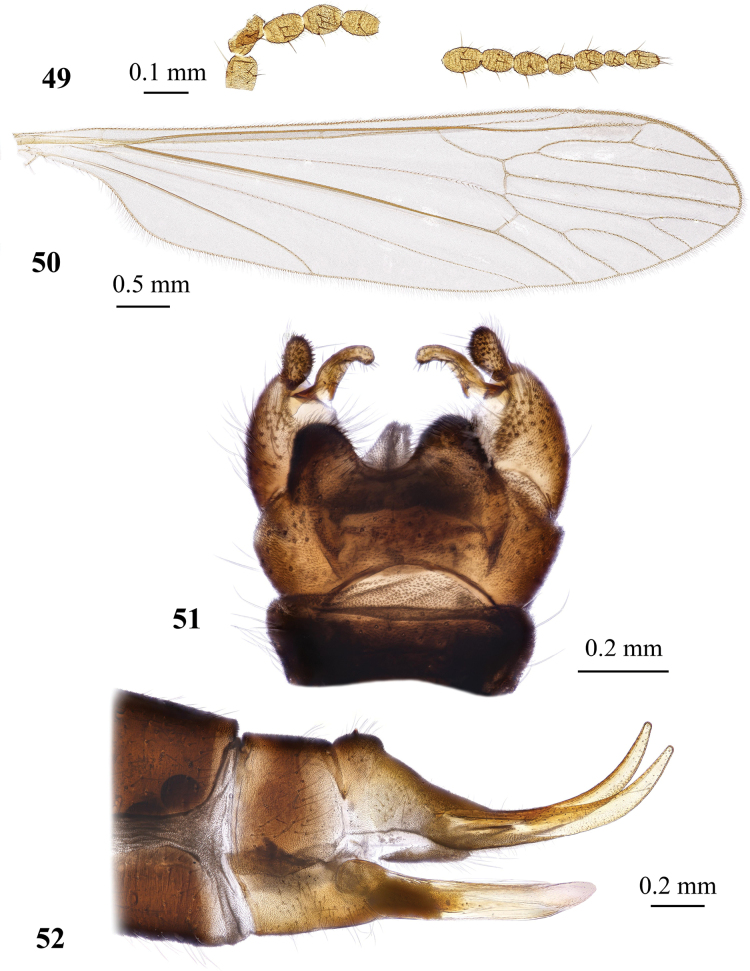
Dicranota (Rhaphidolabis) minuscula Alexander, 1938 **49** antennal flagellum, holotype **50** wing, holotype **51** male genitalia, dorsal view **52** ovipositor, lateral view.

***Head*.** Pale brownish grey because of dense pruinosity, ground colour dark brown. Ground colour more distinct in old specimens. Indistinct darker stripe or narrow line extends medially. Area along eye margin light grey. Sparse, brownish, semi-erect setae cover dorsal area. Eyes widely separated, distance between them at base of antennae approximately equals length of both basal antennomeres taken together. Vertex with distinct rounded tubercle. Antenna dark brown, short, reaching to approx. frontal margin of prescutum, if bent backwards, 0.6 mm long in male, 0.8 mm in female. Scape elongate, nearly cylindrical, ~ 2 × as long as pear-shaped pedicel. Flagellum 12-segmented, basal flagellomere short, barrel-shaped, approximately as long as wide, remaining flagellomeres oval, covered with short light pubescence (Fig. 49). Apical flagellomere large, 1.7 × as long as preceding segment. Longest verticils ~0.5 × as long as respective flagellomeres. Rostrum dark brown, covered with dense light grey pruinosity. Palpus short, dark brown, covered with sparse dark erect setae. Labellum brown.

***Thorax*.** Generally light grey because of dense pruinosity that covers dark brown surface, dorsum brownish grey. Presutural scutum pale brownish grey with three distinct dark brown stripes. Medial stripe not reaching suture caudally, with interrupted pale longitudinal vitta along middle, more distinct posteriorly. Lateral stripe reaches pseudosutural fovea frontally and suture caudally. Scutal lobe lead grey with dark brown central area reaching lateral scutal stripe frontally. Area between scutal lobes pale grey with dark brown frontal margin. Scutellum pale grey with few erect yellowish setae. Mediotergite bluish grey. Pleuron pale grey, bluish dorsally, brownish ventrally. Wing (Fig. 50) long and narrow both in male and female, slightly > 4 × as long as wide, subhyaline, milky, with yellowish base and costal area, iridescent, without darker pattern, except indistinct pale brown stigma. Veins brown, yellowish at wing base. Venation: *Sc* long, reaching far beyond branching point of *R_2+3+4_*, nearly to the proximal margin of stigma. Cross-vein *sc-r* closer to base of *Rs* than to humeral vein. *Rs* 2.6 × as long as *m-cu*, arched. Free end of *R_1_* short, distinctly shorter than *R_2_*. Vein *R_2_* oblique. Distal portions of *R_3_*, *R_4_*, and *R_5_* straight and parallel to each other. Stem of cell *r_3_* long, longer than *m-cu*. Discal cell missing due to reduction of *m-m*. Cell *m_1_* short, its stem 2 × as long as cell itself. Cross-vein *m-cu* nearly its own length beyond branching point of *M*. Vein *CuP* slightly sinuous, *A_1_* slightly arched at distal part. Anal angle large, widely rounded. Halter brown with darker brown knob. Length of male halter 0.8 mm, of female 0.9–1.0 mm. Coxae grey, just fore coxa brownish postero-ventrally. Trochanters yellowish brown. Femora pale brown, slightly more infuscate towards distal end. Tibiae and basal tarsomeres pale brown, distal tarsomeres brown. Male femur I: 4.8 mm long, II: 5.6 mm, III: 5.3 mm, tibia I: 5.6 mm, II: 5.3 mm, III: 5.3 mm, tarsus I: 7.0 mm, II: 5.8 mm, III: 8.1 mm. Female femur I: 4.4–5.1 mm long, II: 4.6–5.4 mm, III: 4.3–5.5 mm, tibia I: 4.2–5.0 mm, II: 4.1–4.8 mm, III: 4.0–5.2 mm, tarsus I: 4.9–6.0 mm, II: 4.5–5.4 mm, III: 3.9–5.2 mm. Claw pale brown, without spines or teeth, basal half widened, distal narrow.

***Abdomen*.** Tergites rusty brown, sternites somewhat paler, both covered with sparse yellowish setae. Pregenital segments dark brown. Membrane between tergites and sternites obscure yellow. Male terminalia (Fig. 51) yellow. Posterior margin of epandrium with deep and wide emargination, lateral lobe rounded, densely covered with long setae. Gonocoxite short and stout, simple, without additional lobes. Interbase wide at base, distal portion narrow and strongly arched. Outer gonostylus elongate, nearly parallel-sided, plate-shaped, distal portion covered with dense blackish spinulae, apex rounded. Inner gonostylus nearly as long as outer gonostylus, but narrower, mesal surface with few small spines apically. Aedeagus straight and narrow, extending beyond caudal end of paramere. Ovipositor (Fig. 52) brownish yellow with pale tips of cercus and hypogynial valve. Cercus arched with tip raised upwards, hypogynial valve straight.

#### Elevation range.

From circa 100 m to nearly 2000 m.

#### Period of activity.

Adults fly from late April through to late October. Probably two generations a year.

#### Habitat.

Wet sparse alder grooves in South Kuriles (Savchenko and Krivolutskaya 1976), *Chosenia* and *Alnus* grooves on margins of deep pits permanently filled with water in river valleys in south Primorye (Savchenko 1983). Semi-natural margins of streams densely covered with shrubs near small villages; margins of small fast-running rocky streams surrounded by mixed forests, with moss densely covering rock surfaces and some sandy bottom areas in South Korea.

#### General distribution.

Korean Peninsula and Far East of Russia.

#### Remark.

This species is recorded from South Korea for the first time.

### ﻿ Dicranota (Rhaphidolabis) neoconsors

Taxon classificationAnimaliaDipteraPediciidae

Alexander, 1938

DE51D7F2-BAEA-54B4-8045-AD4A63735AFE

Figs 53–57, 88

Dicranota (Rhaphidolabis) neoconsors Alexander, 1938b: 153; Savchenko 1983: 43–44; Savchenko 1989: 29.

#### Type material examined.

**North Korea • *Holotype*** ♂ (pinned, antenna, fore leg, wing and genitalia slide mounted); Seren Mountains; alt. 1890 m; 10 October 1937; A. M. Yankovsky leg.; USNM.

#### Other examined material

**(Fig. 88). North Korea** • 1 ex. (pinned, abdomen broken); Chonsani Paiktusan; alt. 1128 m; 26 July 1937; A. M. Yankovsky leg.; USNM • 1 ♀ (pinned); alt. 1829–1920 m; 29 July 1939; A. M. Yankovsky leg.; USNM • 1 ♀ (pinned); Chonsani; alt. 1219 m; 29 April 1940; A. M. Yankovsky leg.; USNM • 1 ♀ (pinned); Chonsani; alt. 1067 m; 26 June 1940; A. M. Yankovsky leg.; USNM • 1 ♀ (pinned); Pontani Paiktusan; alt. 1219 m; 27 June 1940; A. M. Yankovsky leg.; USNM • 2 ♀ (pinned); Pontani Paiktusan; alt. 1676 m; 28 June 1940; A. M. Yankovsky leg.; USNM • 2 ♂ (pinned, genitalia in microvials with glycerol on same pins); Pontani Paiktusan; alt. 1676 m; 28 July 1940; A. M. Yankovsky leg.; USNM • 1 ♀ (pinned); Pontani Paiktusan; alt. 1859 m; 28 July 1940; A. M. Yankovsky leg.; USNM • 2 ♂, 1 ♀ (pinned, genitalia of males in microvials with glycerol on same pins); Pontani Paiktusan; alt. 1219 m; 29 July 1940; A. M. Yankovsky leg.; USNM • 1 ♀ (pinned); Pontani Paiktusan; alt. 1676 m; 31 July 1940; A. M. Yankovsky leg.; USNM • 2 ♀ (pinned); Pontani Paiktusan; alt. 1829 m; 2 August 1940; A. M. Yankovsky leg.; USNM • 1 ♀ (pinned); Pontani Paiktusan; alt. 1942 m; 2 August 1940; A. M. Yankovsky leg.; USNM • 1 ♀ (pinned, ovipositor in microvial with glycerol on same pin); Pontani Paiktusan; alt. 1676–1890 m; 4 August 1940; A. M. Yankovsky leg.; USNM • 1 ♂, 3 ♀ (pinned, male genitalia in microvial with glycerol on same pin); Pontani Paiktusan; alt. 1676 m; 5 August 1940; A. M. Yankovsky leg.; USNM • 1 ♀ (pinned); Pontani Paiktusan; alt. 1859 m; 8 August 1940; A. M. Yankovsky leg.; USNM • 2 ♀ (pinned); Pontani Paiktusan; alt. 1920 m; 8 August 1940; A. M. Yankovsky leg.; USNM • 1 ♀ (pinned); Pontani Paiktusan; alt. 1829 m; 9 August 1940; A. M. Yankovsky leg.; USNM • 1 ♀ (pinned); Pontani Paiktusan; alt. 1859 m; 9 August 1940; A. M. Yankovsky leg.; USNM • 1 ♀ (pinned); Pontani Paiktusan; alt. 1920 m; 10 August 1940; A. M. Yankovsky leg.; USNM • 2 ♀ (pinned); Pontani Paiktusan; alt. 1768 m; 13 August 1940; A. M. Yankovsky leg.; USNM • 1 ♀ (pinned); Pontani Paiktusan; alt. 1920 m; 20 August 1940; A. M. Yankovsky leg.; USNM • 1 ♀ (pinned); Pontani Paiktusan; alt. 1829–1942 m; 25 August 1940; A. M. Yankovsky leg.; USNM; **South Korea** • 1 ♀ (pinned); #37, Hill 1468, 16 mi. NW Chunchon; 38.00000°N, 127.50000°E; alt. 1311 m; 16 September 1954; G. W. Byers leg.; SMEK • 1 ♂, 1 ♀ (pinned, male genitalia in microvial with glycerol on same pin); #38, Hill 1468, 16 mi. NW Chunchon; 38.00000°N, 127.50000°E; alt. 1311 m; 17 September 1954; G. W. Byers leg.; SMEK • 1 ♀ (pinned); Jeollabuk-do, Muju-gun, Seolcheon-myeon, Jangdeok-ri, Gucheondong; 10 June 1972; C.-H. Kim leg.; KUEM • 1 ♀ (in ethanol); Gangwon-do, Goseong-gun, Ganseong-eup, Jinbu-ri; 38.26678°N, 128.35706°E; alt. 497 m; 8 July 2015 (1); S. Kim, S. Podenas leg.; NIBR • 1 ♀ (in ethanol); Gangwon-do, Gapyeong-gun, Buk-myeon, Jeokmok-ri; 37.97627°N, 127.441601°E; alt. 794 m; August – 27 September 2015; KUEM • 1 ♀ (in ethanol); Gyeongsangbuk-do, Gyeongju-si, Jinhyeong-dong, Tohamsan (Mt.); 35.78706°N, 129.34211°E – 35.78947°N, 129.34700°E; 27 May 2016; S. Podenas, H.-M. Baek; NIBR • 1 ♂ (pinned, wing and genitalia slide mounted); Gyeonggi-do, Gapyeong-gun, Gapyeong-eup, Kalbong Natural Recreation Forest; 37.83651°N, 127.46537°E, alt. 178 m; 7 October 2018; S. Podenas leg.; NIBR • 2 ♀ (in ethanol); Gangwon-do, Chuncheon-si, Dongsan-myeon, Kangwon National University Experimental Forest; 37.77909°N, 127.81580°E; alt. 225 m; 9 October 2018; S. Podenas leg.; at light; NIBR.

#### Redescription.

General body colouration: dark brown head, obscure yellow thorax with greyish dorsum, dark brown abdomen. Body length of male ~ 4.5 mm, of female 4.8–7.9 mm. Wing length of male 5.2 mm, of female 5.6–8.6 mm.

**Figures 53–57.**
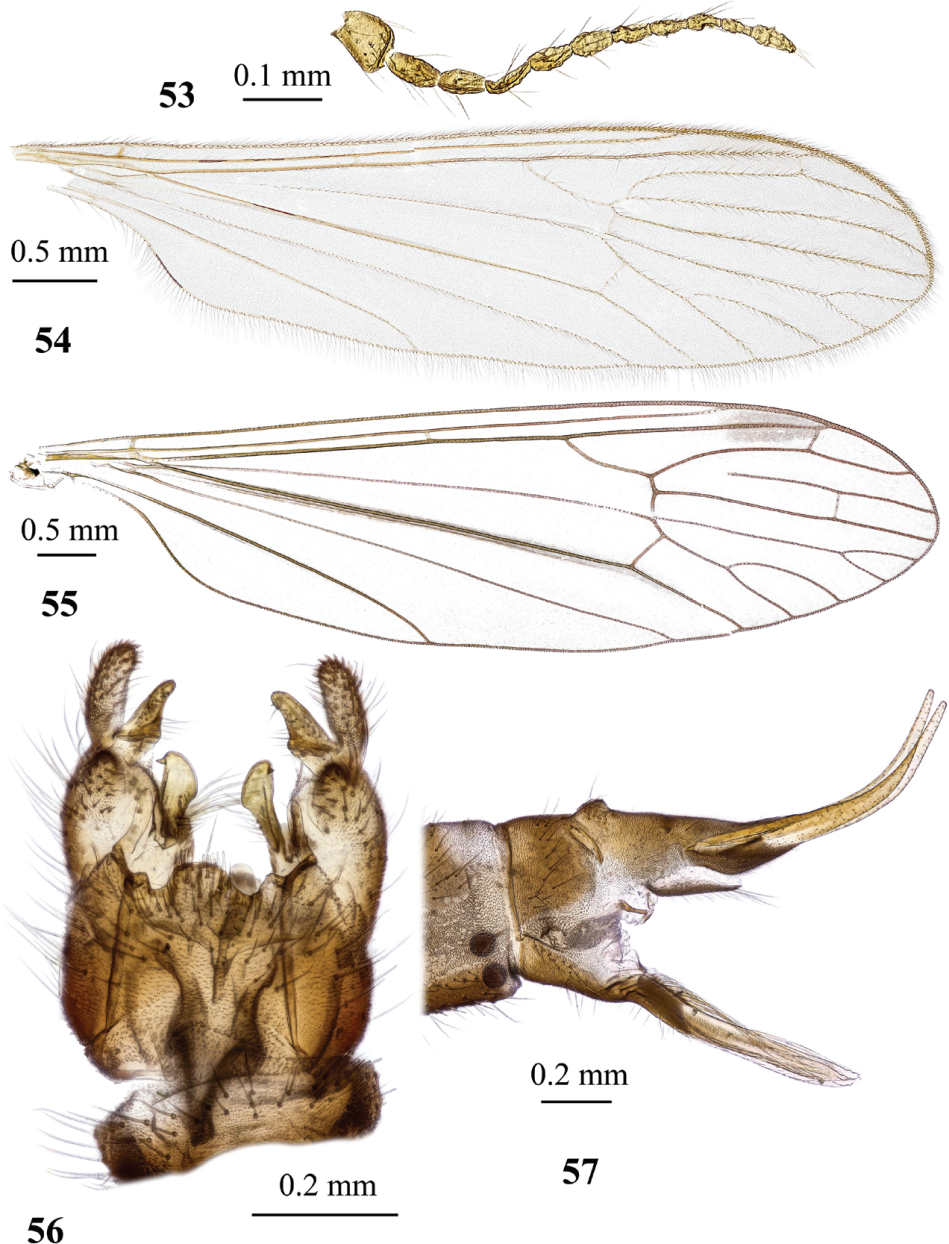
Dicranota (Rhaphidolabis) neoconsors Alexander, 1938 **53** antenna, holotype **54** wing, holotype **55** wing, variation of venation **56** male genitalia, dorsal view **57** ovipositor, lateral view.

***Head*.** Dark brown, paler posteriorly, narrowly light grey along eye margin, covered with dense grey pruinosity and sparse yellowish setae. Vertex with low, slightly darker, rounded tubercle. Eyes widely separated, distance between them at base of antennae slightly exceeds length of scape. Antenna (Fig. 53) short, 0.6 mm long in male, 0.7 mm in female, reaching slightly beyond frontal margin of prescutum, if bent backwards. Scape yellowish brown to brown in male, covered with grey pruinosity, nearly cylindrical, ~2 × as long as succeeding segment. Pedicel cup-shaped, yellowish brown to dark brown. Flagellum dark brown, flagellomeres elongate, basal flagellomere oval, remaining segments wider at base, tapering towards apex. Holotype flagellum 10-segmented, but many specimens of both sexes have 11-segmented flagellum. Apical flagellomere large, 1.6 × as long as preceding segment. Longest verticils nearly reaching length of respective flagellomeres. Rostrum testaceous yellow to pale greyish brown. Basal palpomeres pale, terminal segments dark brown. Labellum pale brown.

***Thorax*.** Generally greyish yellow to brownish, densely covered with dense brownish grey pruinosity. Presutural scutum greyish yellow with yellow lateral margin, with three distinct dark brown stripes. Medial stripe darker than lateral, dark brown, wider frontally, not reaching suture caudally. Lateral stripe paler and more diffuse, extending from pseudosutural fovea frontally to suture caudally. Scutal lobe with diffuse dark area in the middle, lateral margin greyish yellow, area between scutal lobes obscure yellow. Scutellum weakly darkened. Mediotergite weakly darkened with more infuscate ventral margin. Pleuron obscure yellow with darkened ventral area of katepisternum, less visible in older museum specimens. Wing (Figs 54, 55) long and narrow, nearly 4 × as long as wide, subhyaline, milky, with yellowish base, iridescent, without darker pattern, except indistinct pale brown stigma. Veins pale greyish brown, yellowish at wing base. Venation: *Sc* long, reaching far beyond branching point of *R_2+3+4_*, nearly to the proximal margin of stigma. Cross-vein *sc-r* closer to base of *Rs* than to humeral vein. *Rs* 2.3 × as long as *m-cu*, nearly straight. Free end of *R_1_* as long as *R_2_*. Vein *R_2_* transverse. Distal portions of *R_3_*, *R_4_*, and *R_5_* arched, tips of *R_4_* and *R_5_* converging. Stem of cell *r_3_* very short, shorter than *r-m*. Discal cell missing due to reduction of vein *m-m*. Cell *m_1_* short, its stem nearly 3 × as long as cell itself. Cross-vein *m-cu* approximately its own length beyond branching point of *M*. Vein *CuP* straight, *A_1_* slightly arched at distal part. Anal angle large, widely rounded. Halter pale with slightly darker brownish knob. Length of male halter 0.8 mm, of female 1.1 mm. Coxae brownish yellow. Trochanters yellow. Femora and tibiae pale brown, distal tarsomeres darker brown. Male femur I: 4.8 mm long, II: 5.6 mm, III: 5.3 mm, tibia I: 5.6 mm, II: 5.3 mm, III: 5.3 mm, tarsus I: 7.0 mm, II: 5.8 mm, III: 8.1 mm. Female femur I: 3.5 mm long, II: 4.0 mm, III: 3.7–5.6 mm, tibia I: 3.5 mm, II: 3.3 mm, III: 3.4–5.1 mm, tarsus I: 5.0 mm, II: 4.5 mm, III: 4.6–5.3 mm. Claw brownish yellow, without spines or teeth.

***Abdomen*.** Brown to dark brown, covered with greyish pruinosity and sparse erect yellowish setae, basal sternites yellow in male, greyish brown in female. Pregenital segments concolourous with the rest of abdomen. Male terminalia (Fig. 56) yellowish brown. Posterior margin of epandrium with low and wide slightly serrate median lobe covered with dense short but strong setae, lateral lobe long and narrow, finger shaped. Gonocoxite slightly longer than wider, with tuft of long dense setae mesally at base and few small spines at apex, without additional lobes. Interbase large, elongate blade extending beyond apex of gonocoxite, distal half widened, terminating in an acute spine, basal part narrower and finely serrate. Outer gonostylus elongate, fleshy and setose, oval with concave mesal surface. Inner gonostylus nearly as long as outer gonostylus, distal part subglobular, without setae. Aedeagus short. Ovipositor (Fig. 57) yellow. Base of cercus narrowly dark brown, distal part pale yellow, raised upwards. Hypogynial valve nearly straight, yellow to pale yellow.

#### Elevation range.

From 200 m to nearly 2000 m.

#### Period of activity.

Adults fly from the end of April through to the middle of October.

#### Habitats.

Southern slopes of rocky canyons of small streams surrounded by broad leaved forests in the Kedrovaya Padj sanctuary in the Far East of Russia (Savchenko 1983).

#### General distribution.

North and South Koreas, Far East of Russia.

#### Remark.

This species is recorded from South Korea for the first time.

### ﻿ Dicranota (Rhaphidolabis) ompoana

Taxon classificationAnimaliaDipteraPediciidae

Alexander, 1945

326BA982-B5E1-5DD1-8275-889B66148B3B

Figs 58–61, 89

Dicranota (Rhaphidolabis) ompoana Alexander, 1945: 246.

#### Type material examined.

**North Korea • *Holotype*** ♂ (both antennae, fore leg, wing and abdomen slide mounted); Ompo; alt. 213 m; 8 May 1938; A. M. Yankovsky leg.; USNM.

#### Other examined material

**(Fig. 89). North Korea** • 3 ♀ (pinned); Ompo; alt. 76 m; 7 May 1938; A. M. Yankovsky leg.; USNM • 3 ♀ (pinned); Ompo; alt. 91 m; 8 May 1938; A. M. Yankovsky leg.; USNM • 2 ♀ (pinned); Ompo; alt. 91 m; 9 May 1938; A. M. Yankovsky leg.; USNM • 4 ♀ (pinned); Ompo; alt. 61 m; 12 May 1938; A. M. Yankovsky leg.; USNM • 1 ♀ (pinned); Ompo; alt. 61 m; 28 May 1938; A. M. Yankovsky leg.; USNM • 1 ♀ (pinned); Ompo; alt. 122 m; 3 June 1938; A. M. Yankovsky leg.; USNM • 2 ♀ (pinned); Chonsani; alt. 1524 m; 4 July 1940; A. M. Yankovsky leg.; USNM • 3 ♀ (pinned); Chonsani; alt. 1372 m; 6 July 1940; A. M. Yankovsky leg.; USNM • 1 ♂ (pinned, broken genitalia in microvial with glycerol on same pin); Pontani Paiktusan; alt. 1905 m; 26 July 1940; A. M. Yankovsky leg.; USNM • 2 ♀ (pinned); Pontani Paiktusan; alt. 1219 m; 29 July 1940; A. M. Yankovsky leg.; USNM • 2 ♂ (pinned, genitalia in microvials with glycerol on same pins); Pontani Paiktusan; alt. 1676 m; 31 July 1940; A. M. Yankovsky leg.; USNM • 1 ♀ (pinned); Pontani Paiktusan; alt. 1676–1890 m; 4 August 1940; A. M. Yankovsky leg.; USNM • 1 ♂ (pinned, genitalia in microvial with glycerol on same pin); Pontani Paiktusan; alt. 1676 m; 5 August 1940; A. M. Yankovsky leg.; USNM • 1 ♂ (pinned, genitalia in microvial with glycerol on same pin); Pontani Paiktusan; alt. 1890 m; 6 August 1940; A. M. Yankovsky leg.; USNM • 1 ♀ (pinned, ovipositor in microvial with glycerol on same pin); Pontani Paiktusan; alt. 1920 m; 8 August 1940; A. M. Yankovsky leg.; USNM • 1 ♀ (pinned); Pontani Paiktusan; alt. 1859 m; 9 August 1940; A. M. Yankovsky leg.; USNM • 1 ♀ (pinned); Pontani Paiktusan; alt. 1859 m; 10 August 1940; A. M. Yankovsky leg.; USNM • 1 ♀ (pinned); Pontani Paiktusan; alt. 1768 m; 13 August 1940; A. M. Yankovsky leg.; USNM • 2 ♀ (pinned); Pontani Paiktusan; alt. 1829 m; 17 August 1940; A. M. Yankovsky leg.; USNM • 1 ♀ (pinned); Pontani Paiktusan; alt. 1942 m; 23 August 1940; A. M. Yankovsky leg.; USNM • 1 ♀ (pinned); Pontani Paiktusan; alt. 1829–1942 m; 25 August 1940; A. M. Yankovsky leg.; USNM; **South Korea** • 1 ♀ (pinned); Kwangju [Gwangju]; 27 April 1946; S. Kramer leg.; USNM; • 1 ♂ (pinned, genitalia in microvial with glycerol on same pin); Jeollabuk-do, Namwon, Unbong-eup, Hwasu-ri; 35.45345°N, 127.57759°E; alt. 509 m; 6 May 2013 (1); S. Podenas, H. Byun leg.; NIBR • 1 ♀ (pinned); Jeollabuk-do, Namwon, Jucheon-myeon, Gogi-ri; 35.38131°N, 127.48412°E; alt. 450 m; 7 May 2013 (2); S. Podenas, H. Byun leg.; NIBR • 1 ♂ (pinned, genitalia in microvial with glycerol on same pin); Gyeongsangnam-do, Sancheong, Sicheon-myeon, Jungsan-ri; 35.30996°N, 127.75163°E; alt. 709 m; 9 May 2013 (1); S. Podenas leg.; NIBR • 1 ♀ (in ethanol); Gangwon-do, Chuncheon-si, Dongsan-myeon, Kangwon National University Experimental Forest; 37.77909°N, 127.81580°E; alt. 225 m; 9 October 2018; S. Podenas leg.; at light; NIBR • 1 ♂ (in ethanol); Gyeonggi-do, Yongpyeong-gun, Cheongun-myeon, Dowon-ri, Dowon Valley; 37.54514°N, 127.79449°E; alt. 213 m; 10 October 2018 (1); S. Podenas leg.; NIBR.

#### Redescription.

General body colouration grey. Body length of male ~ 6.0 mm, of female 5.1–7.6 mm. Wing length of male 5.3–7.0 mm, of female 6.4–9.2 mm.

**Figures 58–61.**
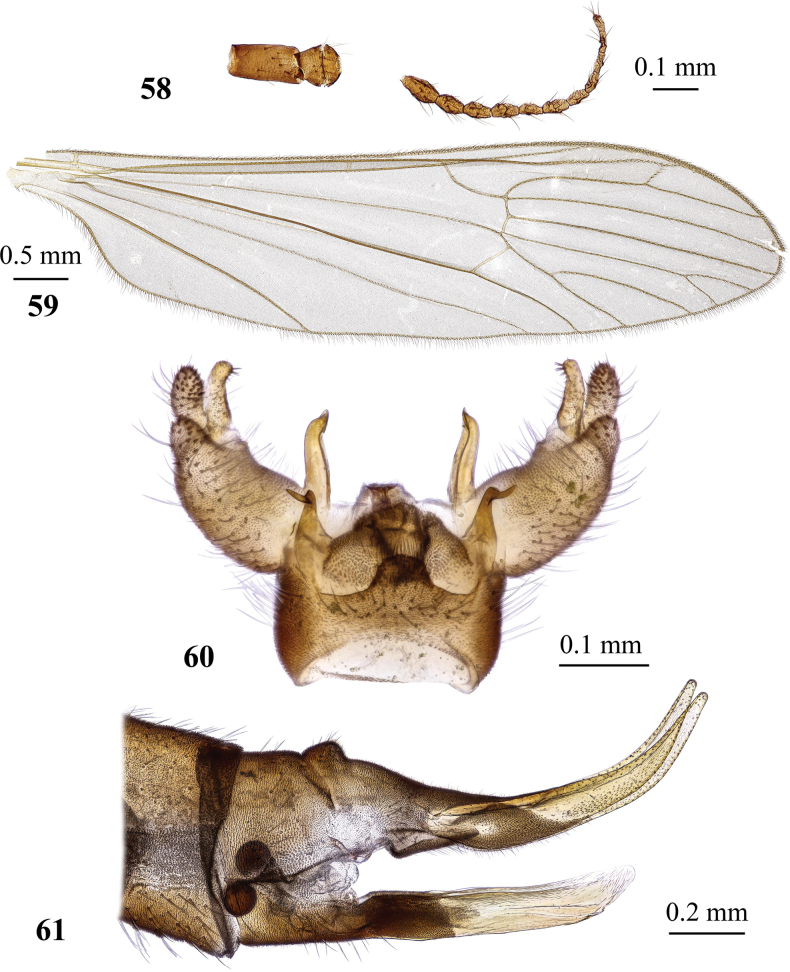
Dicranota (Rhaphidolabis) ompoana Alexander, 1945 **58** antenna, holotype **59** wing, holotype **60** male genitalia, dorsal view **61** ovipositor, lateral view.

***Head*.** Black, densely covered with grey pruinosity and sparse semi-erect yellowish setae. Eyes widely separated, distance between them at base of antennae slightly exceeds length of scape. Vertex with distinct tubercle, covered with yellowish grey pruinosity, top blackish. Antenna (Fig. 58) short, 0.5–0.7 mm long in male, 0.6 mm in female. Scape elongate, subcylindrical, ~ 1.5 × as long as pedicel, dark brown, covered with sparse greyish pruinosity and bearing few erect setae on dorsal surface. Pedicel wider distally, blackish, covered with few short dark setae. Flagellum black, 11-segmented, flagellomeres elongate, oval, basal flagellomere 1.5 × as long as succeeding segment. Apical flagellomere relatively large, 0.9 × as long as preceding segment. Longest verticils 0.7 × as long as respective flagellomeres. Rostrum dark brown to black. Palpus black. Labellum large, obscure yellow with narrowly darkened margins.

***Thorax*.** Pronotum grey laterally, greyish brown dorsally. Presutural scutum obscure brownish to bluish grey with three distinct dark brown stripes. Medial stripe without paler line along middle, or with very indistinct line posteriorly, darker frontally, less distinct caudally, not reaching suture. Lateral stripe short, nearly reaches pseudosutural fovea frontally and suture caudally, more distinct frontally, fading caudally. Scutal lobe with diffuse darker area extending lateral scutal stripe. Area between scutal lobes pale yellowish, greyish brown, colour varies depending on angle of light. Scutellum and mediotergite pale grey. Pleuron grey with darkened ventral area of katepisternum and meron. Dorsopleural membrane obscure yellow. Wing (Fig. 59) long and narrow, 4 × as long as wide, subhyaline, milky, with yellowish base, iridescent, without darker pattern, except indistinct pale brown stigma. Veins pale greyish brown, yellowish at wing base. Venation: *Sc* long, reaching beyond level of cell *m_3_* base, to approx. proximal margin of stigma. Cross-vein *sc-r* closer to base of *Rs* than to humeral vein. *Rs* 1.6 × as long as *m-cu*, arched. Free end of *R_1_* very short, much shorter than *R_2_*. Vein *R_2_* oblique. Wing cell *r_2_* without supernumerary cross-vein, but one female from Ompo has this cross-vein on the left wing and missing on the right wing. Distal portions of *R_3_*, *R_4_*, and *R_5_* straight, parallel to each other. Cell *r_3_* without stem. Discal cell missing due to reduction of vein *m-m*. Cell *m_1_* short, its stem 3 × as long as cell itself. Cross-vein *m-cu* short distance beyond branching point of *M*. Vein *CuP* slightly sinuous, *A_1_* slightly arched. Anal angle large, widely rounded. Halter yellow with slightly darkened knob. Length of male halter 1.0 mm, of female 0.9–1.0 mm. Fore and middle coxae pale, densely covered with grey pruinosity, posterior coxae yellow. Trochanters yellow. Femora and tibiae pale brown to brown with slightly darkened apices, basal tarsomeres pale brown, distal tarsomeres darker brown. Male femur I: 3.9–4.8 mm long, II: 5.1 mm, III: 4.3–5.0 mm, tibia I: 3.7–4.7 mm, II: 4.6 mm, III: 4.1–4.8 mm, tarsus I: 4.9–6.8 mm, II: 5.5 mm, III: 4.7–5.3 mm. Female femur I: 3.5 mm long, II: 4.1–5.5 mm, III: 3.7–5.6 mm, tibia I: 3.5 mm, II: 3.6–4.9 mm, III: 3.4–5.1 mm, tarsus I: 5.0 mm, II: 3.7–4.9 mm, III: 4.6–5.3 mm. Claw dark brown, distinctly wider at base than distally.

***Abdomen*.** Semi-polished, yellowish to greyish brown, covered with pale semi adjacent setae. Posterior margins of segments pale, wider on tergites. Basal sternite pale grey frontally. Pregenital segment darker brown. Male terminalia (Fig. 60) dark brown. Posterior margin of epandrium with low and wide rectangular median lobe covered with dense short setae, lateral lobe long and narrow, strongly curved. Gonocoxite short and wide, ~1.5 × as long as wide, with apex slightly produced and covered with scarce strong short spines. Interbase yellow, large, nearly as long as gonocoxite itself, tip slightly curved, distal margin finely serrate. Outer gonostylus elongate, oval, fleshy and setose, distal part covered with short black strong spines. Inner gonostylus exceeds in length outer gonostylus, arched, fleshy and setose, distal half of mesal margin with few short spines. Aedeagus short, paramere slightly curved, extending well beyond tip of aedeagus. Ovipositor (Fig. 61) yellow with dark brown ninth and tenth tergites and eight sternite. Basal half of cercus brownish, distal part paler. Hypogynial valve nearly straight, long and parallel-sided with pale rounded tip. Setae along dorsal margin of valve, common for most *Rhaphidolabis*, strongly reduced.

#### Elevation range.

From < 100 m to nearly 2000 m.

#### Period of activity.

Adults fly from late April through middle of October.

#### Habitats.

Margins of small fast running streams with rocky bottom, densely covered with shrubs. Adults are sometimes found hiding under bridges.

#### General distribution.

North and South Koreas.

#### Remark.

Species recorded from South Korea for the first time.

### ﻿ Dicranota (Rhaphidolabis) polymera

Taxon classificationAnimaliaDipteraPediciidae

Alexander, 1933

D4CD0FF4-EE6A-5006-AFEB-A17423D9BE93

Figs 62–65, 90

Dicranota (Rhaphidolabis) polymera Alexander, 1933: 537–538, figs 18, 39; Alexander 1954: 292.

#### Type material examined.

**Japan • *Holotype*** ♂ (antenna, fore leg, wing and genitalia slide mounted); Kyushu, Mt. Wakasugi; 15–16 November 1930; T. Esaki et al. leg.; USNM; ***Allotype*** • 1 ♀ (pinned, antenna, wing and two legs slide mounted); Shikoku, Sugitate; 30 March 1952; F. Takechi leg.; USNM 2012845.

#### Other examined material

**(Fig. 90). South Korea** • 1 ♂ (pinned); #8, Central National Forest, 18 miles NE Seoul; 28 May 1954; G. W. Byers leg.; USNM • 1 ♂ (pinned); #12, Hwy. #20, 8 mi. SW Kangnung; 37.70000°N, 128.78333°E; alt. 587 m; 8 June 1954; G. W. Byers leg.; USNM • 1 ♂ (pinned, genitalia in microvial with glycerol on same pin); #28, Central National Forest, 18 miles NE Seoul; alt. 107–152 m; 22 August 1954; G. W. Byers leg.; SMEK • 1 ♂ (pinned, genitalia in microvial with glycerol on same pin); #39, Central National Forest, 18 miles NE Seoul; alt. 107–152 m; 26 September 1954; G. W. Byers leg.; SMEK • 1 ♂ (pinned); #39, Central National Forest, 18 miles NE Seoul; alt. 107–152 m; 26 September 1954; G. W. Byers leg.; USNM • 2 ♂ (pinned, genitalia of 1 ♂ in microvial with glycerol on same pin); Jeollanam-do, Gurye-gun, Masan-myeon, Hwangjeon-ri; 35.24366°N, 127.48964°E; alt. 101 m; 8 May 2013 (1); S. Podenas leg.; NIBR • 1 ♀ (pinned); Jeollanam-do, Gurye-gun, Toji-myeon, Naeseo-ri, Jirisan National Park, Piagol valley; 35.26580°N, 127.58128°E; alt. 378 m; 10 May 2013; S. Podenas leg.; NIBR • 1 ♂ (in ethanol); Jeollanam-do, Gurye-gun, Toji-myeon, Naeseo-ri, Jirisan National Park, Piagol valley; 35.26580°N, 127.58128°E; alt. 378 m; 12 May 2013 (2); V. Podeniene leg.; NIBR • 2 ♂ (in ethanol); Gyeonggi-do, Gapyeong-gun, Buk-myeon, Hwaak-ri; 37.98402°N, 127.52676°E; alt. 579 m; 20 August 2014 (2); S. Podenas leg.; NIBR • 1 ♂ (pinned); Jeollanam-do, Gurye-gun, Toji-myeon, Naeseo-ri, Jirisan National Park, Piagol valley; 35.27177°N, 127.57146°E; alt. 490 m; 24 April 2015 (4); S. Podenas leg.; NIBR • 1 ♂ (pinned), 14 ♂, 4 ♀ (in ethanol); Jeollanam-do, Gurye-gun, Toji-myeon, Naeseo-ri, Jirisan National Park, Piagol valley; 35.28589°N, 127.55605°E; alt. 773 m; 30 April 2015 (1); S. Podenas leg.; NIBR • 1 ♂ (pinned, genitalia in microvial with glycerol on same pin); Jeollanam-do, Gurye-gun, Toji-myeon, Naeseo-ri, Jirisan National Park, Piagol valley; 35.27177°N, 127.57146°E; alt. 490 m; 2 May 2015 (1); S. Podenas leg.; NIBR • 1 ♂ (pinned); Jeollanam-do, Gurye-gun, Toji-myeon, Naeseo-ri, Jirisan National Park, Piagol valley; 35.27177°N, 127.57146°E; alt. 490 m; 28 June 2015 (1); S. Podenas leg.; NIBR • 1 ♂ (pinned); Gangwon-do, Pyeonchang-gun, Jinbu-myeon, Dongsan-ri, Odaesan National Park; 37.73767°N, 128.59166°E; alt. 730 m; 6 July 2015 (1); S. Podenas leg.; NIBR • 1 ♀ (pinned); Gyeonggi-do, Yangju-si, Jangheung-myeon, Uldae-ri; 37.74258°N, 127.00329°E; alt. 162 m; 19 October 2018; A. Petrasiunas leg.; stream margin; NRC • 1 ♂ (in ethanol); Jeollanam-do, Gurye-gun, Toji-myeon, Naeseo-ri, Jirisan National Park, Piagol valley; 35.27333°N, 127.56924°E; alt. 546 m; 25 June 2019 (1); S. Podenas leg.; NIBR • 2 ♂ (in ethanol); Jeollanam-do, Gurye-gun, Gwangui-myeon, Nogodan-ro; 35.29250°N, 127.49548°E; alt. 696 m; 28 June 2019 (1); S. Podenas leg.; NIBR; **Japan** • 1 ♀ (pinned, antenna, wing, fore and hind legs slide mounted); Honshu, Kurokawa-Echigo [Niigata prefecture, Tainai City, Kanomata River in Kurokawa]; 18 July 1954; K. Baba leg.; USNM 2012845 • 1 ♂ (pinned, wing, fore leg and genitalia slide mounted); Honshu, Kurokawa-Echigo; 15 October 1954; K. Baba leg.; USNM 2012845 • 1 ♀ (wing and hind leg slide mounted); Shikoku, Kuroson, Tosa; alt. 300–400 m; 29 April 1956; T. Yano leg.; USNM.

#### Redescription.

General body colouration dark brown to dark grey (old museum specimens could be paler). Body length of male ~ 6.0, of female 6.8–9.0 mm. Wing length of male 7.0–7.9 mm, of female 7.7–9.5 mm.

**Figures 62–65.**
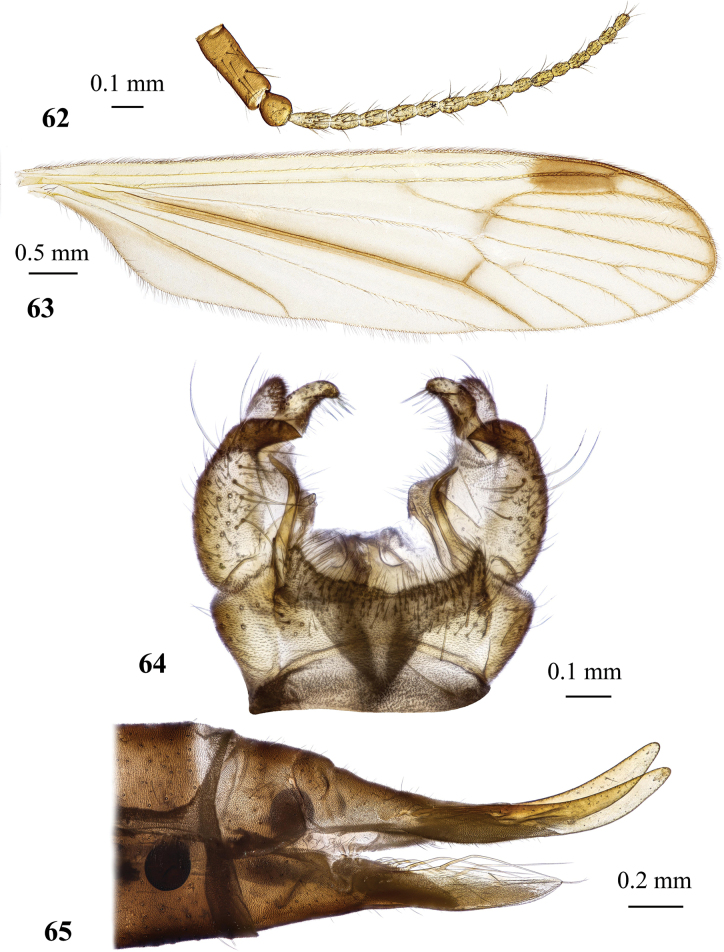
Dicranota (Rhaphidolabis) polymera Alexander, 1933 **62** antenna, holotype **63** wing **64** male genitalia, dorsal view **65** ovipositor, lateral view.

***Head*.** Dark grey, narrowly paler along eye margin, somewhat bluish caudally, silvery frontally, covered with semi-erect dark brown setae. Eyes widely separated, distance between them at base of antennae slightly exceeds length of scape. Tubercle on vertex low, with narrow darker line along middle. Antenna (Fig. 62) medium-long, reaching beyond frontal margin of prescutum, if bent backwards, 1.5 mm long both in male and female. Scape long, nearly cylindrical, 2.5 × as long as pedicel, dark brown, covered with sparse greyish pruinosity and bearing few erect setae on dorsal surface. Pedicel subglobular, brown, bearing few short dark setae. Flagellum black, 15-segmented. Basal flagellomere elongate, widening distally, 1.5 × as long as second segment, succeeding flagellomeres elongate, barrel-shaped. Apical flagellomere relatively large, approximately as long as preceding segment. Longest verticils nearly as long as respective flagellomeres. Rostrum black. Palpus dark brown with blackish setae. Labellum large, brown, sparsely dusted with grey.

***Thorax*.** Pronotum dark brown dorsally and grey laterally, covered with erect brown setae medially. Presutural scutum dark bluish grey with three dark brown stripes. Medial stripe distinct, nearly reaching suture caudally, separated longitudinally by paler line along middle in Japanese specimens, such line is missing in Korean specimens. Lateral stripe less distinct, short, nearly reaches pseudosutural fovea frontally, extends to suture caudally. Scutal lobe grey with diffuse darker area in the middle, reaching caudal end of lateral scutal stripe. Area between scutal lobes brownish. Scutellum greyish brown with yellowish lateral margin, covered with erect setae. Mediotergite brown, greyish frontally, yellowish caudally. Pleuron bluish grey, semi-polished, with slightly darker ventral area of katepisternum. Dorsopleural membrane pale grey. Wing (Fig. 63) long and narrow, 3.9 × as long as wide, distinctly brownish, paler at base, iridescent. Stigma distinct, elongate, brown. Indistinct darker area surrounds origin of *Rs*, cord, cubital and anal veins, narrowly darkened also distal wing margin and base of anal angle. Veins brown, yellowish at wing base. Venation: *Sc* long, reaching proximal margin of stigma. Cross-vein *sc-r* distinctly closer to base of *Rs* than to humeral vein, sometimes missing, like in Fig. 63. *Rs* longer than in most *Rhaphidolabis*, 3 × as long as *m-cu*, slightly arched. Free end of *R_1_* very short, shorter than *R_2_*. Vein *R_2_* transverse. Distal portions of *R_3_*, *R_4_*, *R_5_* and *M_1_* straight, parallel to each other. Cell *r_3_* with short stem. Discal cell missing due to reduction of vein *m-m*. Cell *m_1_* rather long, its stem only 1.6 × as long as cell itself. Cross-vein *m-cu* less than its own length beyond branching point of *M*. Vein *CuP* straight, *A_1_* slightly arched before wing margin. Anal angle large, widely rounded. Stem of halter pale with yellowish base. Knob dark brown with pale base. Length of male and female halter 1.2 mm. Coxae obscure yellow, covered with erect yellow setae, denser on fore coxa. Trochanters yellow with narrowly blackened distal margin. Femora brown with yellowish bases and slightly darkened apices, tibiae yellowish brown with slightly darkened apices, basal tarsomeres brown, distal tarsomeres dark brown. Male femur I: 5.4 mm long, II: 5.5 mm, III: 5.9 mm, tibia I: 5.4 mm, II: 5.2 mm, III: 5.7 mm, tarsus I: 5.7 mm, II: 5.2 mm, III: 5.4 mm. Female femur I: 4.3–4.5 mm long, II: 5.2 mm, III: 5.2 mm, tibia I: 4.4–4.7 mm, II: 4.4 mm, III: 5.2 mm, tarsus I: 4.2–5.0 mm, II: 4.8 mm, III: 5.3 mm. Claw brown, just slightly arched.

***Abdomen*.** Semi-polished, dark greyish brown, covered with sparse pale erect setae. Posterior margins of segments pale grey. Male terminalia (Fig. 64) yellowish brown. Posterior margin of epandrium widely concave and slightly serrate, densely setose; postero-lateral angle with long curved, rod-shaped lobe that narrows towards distal end. This lobe mistakenly was marked as interbase in original description (Alexander 1933), true interbase distinctly shorter straight blade with wider base. Gonocoxite short and wide, length just slightly exceeds width, mesal margin swollen at base, apex produced into large slightly curved spine-shaped lobe. Outer gonostylus pale, elongate, round-apexed, fleshy and setose, distal and mesal areas covered with short blackish spinules. Inner gonostylus exceeds in length outer gonostylus, arched, fleshy and setose, mesal surface with longer setae. Aedeagus short with bifid apex, paramere elongate. Ovipositor (Fig. 65) yellow with yellow tenth tergite. Base of cercus and hypogynial valve slightly infuscated. Cercus slightly arched, blunt apexed. Hypogynial valve straight, narrowing towards apex, dorsal margin with set of long strong setae.

#### Elevation range.

From 100 m to nearly 800 m.

#### Period of activity.

Adults fly from late April through to late October in South Korea; they are active from late March to mid-November in Japan.

#### Habitats.

Adults fly close to springs, small mountainous streams and rivulets, shaded by mixed forests and shrubs with sparse grassy vegetation along the margins. Males sometimes seen fly around tree trunks even further from the water, probably in search of females. This species was not attracted to light even in places where it was collected with nets during the day.

#### General distribution.

South Korea, Kyushu and Shikoku islands of Japan.

#### Remark.

This species is recorded from the Korean Peninsula for the first time.

### ﻿ Dicranota (Rhaphidolabis) seoi

Taxon classificationAnimaliaDipteraPediciidae

Podenas sp. nov.

3D143425-86D9-5A47-8D01-BB43890E5053

https://zoobank.org/F2B18870-CEA7-49F9-871F-D4F616005023

Figs 66–68, 91

#### Type material

**(Fig. 91). South Korea** • ***Holotype*** ♂ (in ethanol); Gangwon-do, Chuncheon-si, Namsan-myeon, Gongchon-ri; 37.81159°N, 127.64919°E; alt. 131 m; 7 October 2018; S. Podenas leg.; net; NIBR. ***Paratypes*** • 1 ♂, 1 ♀ (in ethanol); Jeollanam-do, Gurye-gun, Toji-myeon, Naeseo-ri, Jirisan National Park, Piagol valley; 35.26590°N, 127.58096°E; alt. 446 m; 24 April 2015; S. Podenas leg.; at light; NIBR.

#### Diagnosis.

Medium-sized dark brown species. Wing semi-translucent, milky without darker areas except brownish stigma. Antenna 14-segmented with large apical segment. Epandrium of male genitalia with straight caudal margin bordered with small lateral lobe. Gonocoxite with rounded dorso-apical lobe. Both gonostyli elongate. Gonostyli and gonocoxal lobe covered with short pale spines. Interbase large, flat, with strong spine dorso-caudally. Aedeagus short with blunt apex. Ovipositor yellow with arched cercus and straight hypogynial valve.

#### Etymology.

The species is named after the Korean entomologist Dr Hong-Yul Seo, who not only studied Korean aphids, but did a lot for other groups of insects. He is a very warm person, our friend and colleague, and with whom we collected together on that cold October day when the holotype of this species was found.

#### Description.

General body colouration dark brown. Male body length 10.0 mm, wing length 9.2 mm.

***Head*.** Dark greyish brown, narrowly paler along eye margin, covered with sparse semi-erect dark setae dorsally, longer frontally, shorter caudally. Eyes widely separated, distance between them at base of antenna nearly equals length of scape. Male antenna 1.5 mm long, reaching to approx. frontal margin of prescutum if bent backwards, female antenna not reaching frontal margin of prescutum if bent backwards. Scape dark brown, subcylindrical, bearing few erect setae dorsally. Pedicel somewhat paler, slightly widening towards distal end. Flagellum 12-segmented in both sexes, brown, basal flagellomere slightly elongate, similar to pedicel, succeeding flagellomeres short, subglobular, distal flagellomeres slightly elongate, apical flagellomere large, distinctly longer than preceding segment, especially in female. Verticils short, not reaching length of respective flagellomeres. Rostrum and palpus brown, labellum pale.

**Figures 66–68.**
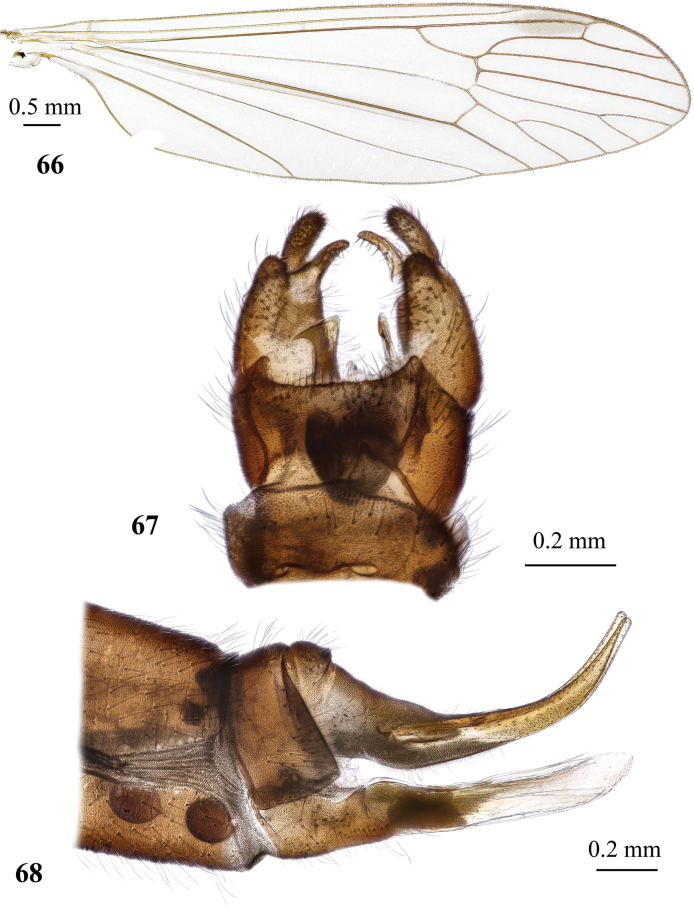
Dicranota (Rhaphidolabis) seoi Podenas, sp. nov., paratypes **66** wing **67** male genitalia, dorsal view **68** ovipositor, lateral view.

***Thorax*.** Uniformly dark brown. Cervical sclerites paler. Pronotum with yellowish lateral margin, bearing few erect whitish setae dorsally. Presutural scutum dark brown with three longitudinal stripes. Medial stripe very wide, not reaching suture caudally, area between caudal margin of stripe and suture yellowish. Stripes separated by grey area, covered with erect pale setae. Pseudosutural fovea indistinct. Prothoracic spiracle surrounded by yellowish membrane. Scutal lobe dark brown, area between lobes yellowish. Scutellum yellowish brown. Mediotergite dark brown. Pleuron uniformly dark brown, just ventral margin of katepisternum narrowly blackish. Wing (Fig. 66) elongate, length/width ratio 3.8, widest slightly before tip of vein *CuP*, semi-translucent, milky, yellowish at base, without any darker spots besides brownish stigma. Veins greyish to brownish, yellowish at wing base. Venation: *Sc* long, reaching wing margin slightly beyond frontal margin of stigma, far beyond of *R_2+3+4_* branching point, *sc-r* closer to origin of radial sector than to humeral vein. *Rs* short, 2.3 × as long as cross-vein *m-cu*, slightly arched. Free end of *R_1_* short, approximately as long as *R_2_*. Vein *R_2_* nearly transverse. *R_3_*, *R_4_*, and *R_5_* nearly straight and parallel to each other. Cell *r_3_* with distinct stem, which is as long as cross-vein *r-m*. Discal cell missing due to atrophy of vein *m-m*. Cell *m_1_* rather long, its stem 1.8 × as long as cell itself. Cross-vein *m-cu* half of its own length beyond branching point of *M*. Vein *CuP* nearly straight, *A_1_* slightly arched before wing margin. Anal angle wide and rounded. Halter long, uniformly pale, knob not darker than stem in male, slightly infuscate in female. Length of male halter 1.2 mm. Fore coxa dorsally brown, ventrally obscure yellow, brown area approximately equals yellow. Middle coxa brown with yellowish distal part, posterior coxa entirely brown. Trochanters obscure yellow with narrowly blackened distal margin. Femora brown with widely pale base and darker distal part. Tibiae brown with indistinctly darker distal part. Tarsomeres brown, except dark brown last segment. Male femur I: 3.3–3.8 mm long, II: 4.0 mm, III: 4.0–4.4 mm, tibia I: 3.4–3.8 mm, II: 3.2–3.4 mm, III: 3.7–4.1 mm, tarsus I: 4.2–5.2 mm, II: 3.6–3.9 mm, III: 3.8–4.6 mm. Female femur I: 3.3–4.0 mm long, II: 3.8 mm, III: 4.0–4.5 mm, tibia I: 3.0 mm, II: 2.9 mm, III: 2.9–3.8 mm, tarsus I: 3.3 mm, II: 2.8 mm, III: 2.9–3.7 mm. Claw small, brownish, slightly arched, with subbasal spine.

***Abdomen*.** Brown, slightly dusted with grey, basal sternite yellowish. Both tergites and sternites covered with very sparse short pale setae. Male terminalia (Fig. 67) brown with yellowish gonocoxites. Posterior margin of epandrium straight with small serration, covered with short pale setae, lateral margin with small simple, triangle-shaped lobe. Gonocoxite elongate, ~2 × as long as wide, with rounded lobe dorso-apically. Mesal margin of lobe covered with sparse short, spine-shaped setae. Two pairs of gonostyli. Outer gonostylus elongate, brown, fleshy and setose, round-apexed. Mesal and caudal margins covered with pale short spines. Inner gonostylus long and narrow, wider at base, slightly arched, pale brown, nearly as long as outer gonostylus. Mesal margin with few long pale spines. Interbase large, wide plate-shaped, dorso-caudal angle extended into large strong spine, ventral margin finely serrate. Aedeagus short, blunt-apexed. Paramere elongate, slightly curved. Ovipositor (Fig. 68) brownish yellow with pale distal part of hypogynial valve. Cercus uniformly brownish yellow, just tip paler. Distal part distinctly narrower, raised upwards, apex blunt. Hypogynial valve straight, brownish yellow at base. Dorsal margin with few short subbasal setae, reaching to ~ 1/4 of valve. Two spermathecae small, subglobular.

#### Habitat.

Margins of fast running mountainous streams with rocky bottom. Slopes covered with mixed forest and sparse herbaceous vegetation. Preimaginal stages unknown. Both males and females attracted to light.

#### Elevation.

From 100 m to 450 m.

#### Period of activity.

Two generations per year, one flying early in spring at the end of April, the second late in the season at the beginning of October.

#### Distribution.

Currently known only from South Korea, but probably has a wider distribution throughout the country, because both localities are far from each other.

#### Remarks.

Generally Dicranota (R.) seoi Podenas, sp. nov. resembles D. (R.) minuscula: both are comparatively large dark species. The wings of both species are similar, without a dark pattern, just the stigma of D. (R.) seoi Podenas, sp. nov. is slightly darker, the stem of cell *m_3_* is slightly longer in D. (R.) minuscula, and the cross-vein *R_2_* slightly more oblique in D. (R.) minuscula. These characters are rather stable, but the best characters for species discrimination are found in the male terminalia. The easiest way to tell both species apart is through the caudal margin of the epandrium, that is straight with a small lateral lobe in D. (R.) seoi Podenas, sp. nov. and deeply concave with a large rounded lateral lobe in D. (R.) minuscula. The gonocoxite of D. (R.) seoi Podenas, sp. nov. bears a distinct dorso-apical lobe, while that of D. (R.) minuscula is simple, without such a lobe. Both gonostyli of D. (R.) seoi Podenas, sp. nov. approximately equal in length, outer gonostylus distinctly shorter than inner gonostylus in D. (R.) minuscula. Females of both species can be separated based on the structure of the cercus, the distal part of which is narrower in D. (R.) seoi Podenas, sp. nov. than in D. (R.) minuscula. A good external character separating D. (R.) seoi Podenas, sp. nov. from other Korean *Rhaphidolabis* and especially from the similar D. (R.) ompoana is the unique 14-segmented antenna. Dicranota (R.) ompoana, like most other Korean *Rhaphidolabis* have 13-segmented antenna.

We expect D. (R.) seoi Podenas, sp. nov. to be more abundant in Korea, but because adults fly only early in the spring and late in the fall, when most crane flies and other insects are inactive, few entomologists collect in the field; thus they are not well represented in entomological collections.

### ﻿ Dicranota (Rhaphidolabis) squarrosa

Taxon classificationAnimaliaDipteraPediciidae

Savchenko, 1976

AEB9B234-C009-598E-9686-2ED1522B4728

Figs 69–71, 92

Dicranota (Rhaphidolabis) squarrosa : Savchenko and Krivolutskaya 1976: 38, 43, 44, fig. 14 (as D. (R.) squarosa);
Dicranota (Rhaphidolabis) squarosa : Savchenko 1989: 29.

#### Examined material

**(Fig. 92). North Korea** • 1 ♂ (pinned, genitalia in microvial with glycerol on same pin); Pontani Paiktusan; alt. 1676 m; 28 July 1940; A. M. Yankovsky leg.; USNM • 1 ♂ (pinned, genitalia in microvial with glycerol on same pin); Pontani Paiktusan; alt. 1859 m; 28 July 1940; A. M. Yankovsky leg.; USNM • 1 ♀ (pinned); Pontani Paiktusan; alt. 1524 m; 29 July 1940; A. M. Yankovsky leg.; USNM • 1 ♂ (pinned, genitalia in microvial with glycerol on same pin); Pontani Paiktusan; alt. 1676 m; 31 July 1940; A. M. Yankovsky leg.; USNM • 3 ♂ (pinned, genitalia in microvials with glycerol on same pins), Pontani Paiktusan, alt. 1676–1890 m, 4 August 1940, A. M. Yankovsky leg.; USNM.

#### Redescription.

General body colouration yellowish brown. Body length of male ~ 5.5 mm, of female ~ 6.0 mm. Wing length of male 5.5–6.0 mm, of female ~ 6.5 mm.

***Head*.** Grey because of dense yellowish grey pruinosity, ground colour dark brown, covered with semi-erect setae, dark brown at base turning yellowish towards tips. Eyes widely separated, distance between them at base of antennae slightly exceeds length of scape. Tubercle on vertex low, indistinctly darkened along middle. Antenna medium-long, reaching frontal margin of prescutum at most, if bent backwards, 0.5 mm long in male, 0.7 mm in female. Scape long, nearly cylindrical, dark brownish grey because of dense grey pruinosity, bearing few short dark setae dorsally. Pedicel subglobular, concolourous with scape. Flagellum dark brown, 11-segmented. Basal flagellomere elongate, succeeding segments barrel-shaped. Apical flagellomere smaller than preceding segment. Longest verticils nearly as long as respective flagellomeres. Flagellum covered with short pale pubescence. Rostrum brown. Palpus dark brown. Labellum large, pale brown.

**Figures 69–71.**
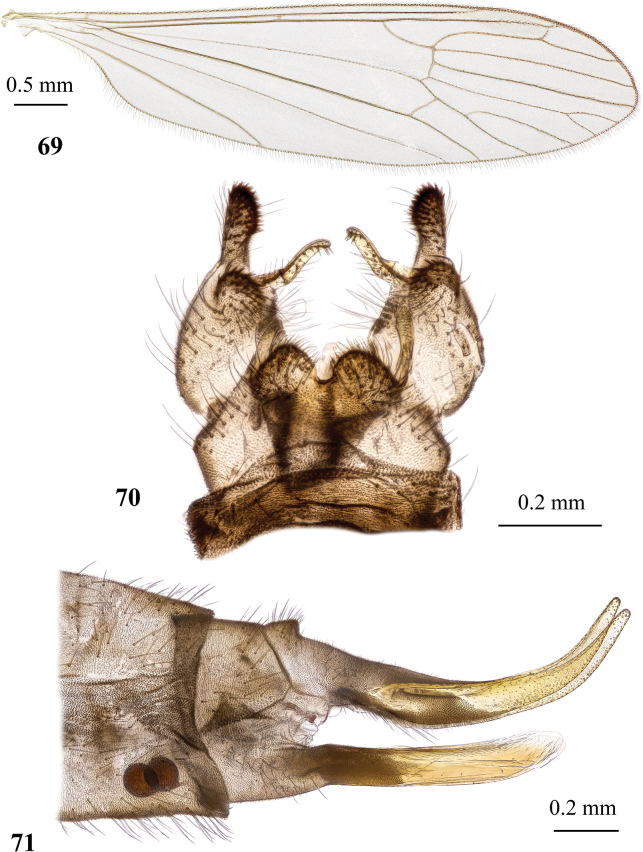
Dicranota (Rhaphidolabis) squarrosa Savchenko, 1976 **69** wing **70** male genitalia, dorsal view **71** ovipositor, lateral view.

***Thorax*.** Pronotum brown, dusted with yellowish grey, covered with erect pale setae medially. Presutural scutum brownish grey with three dark brown longitudinal stripes. Medial stripe distinct, dark brown with indistinct pale medial line, more distinct caudally, missing frontally. Lateral stripe less distinct, short, nearly reaches pseudosutural fovea frontally, extends to scutal lobe caudally. Area between medial and lateral stripe more rusty brown. Scutal lobe dark grey, nearly concolourous with lateral scutal stripe, only lateral and medial margins paler grey. Scutellum uniformly grey. Mediotergite yellowish brown with sparse cover of grey pruinosity. Pleuron uniformly brownish grey because of dense cover of grey pruinosity. Dorsopleural membrane yellowish grey. Wing (Fig. 69) long and narrow, 4 × as long as wide, translucent with slight greyish tinge, paler at base, iridescent. No dark areas besides indistinct stigma. Veins greyish, yellowish at wing base. Venation: *Sc* long, reaching proximal margin of stigma. Cross-vein *sc-r* closer to origin of *Rs* than to humeral vein. *Rs* short, 1.8 × as long as *m-cu*, slightly arched. Free end of *R_1_* very short, distinctly shorter than *R_2_*. Vein *R_2_* slightly oblique. Distal portions of *R_3_*, *R_4_*, and *R_5_* straight, parallel to each other. Cell *r_3_* without stem. Discal cell missing due to reduction of vein *m-m*. Cell *m_1_* short, its stem nearly 3 × as long as cell itself. Cross-vein *m-cu* less than its own length beyond branching point of *M*. Vein *CuP* nearly straight, *A_1_* slightly arched at distal end. Anal angle large, widely rounded. Stem of halter pale to greyish with yellowish base. Knob darker brownish grey. Length of male halter 0.5 mm. Coxae yellowish grey, covered with sparse erect yellowish setae, denser on fore coxa. Trochanters, especially fore and middle more intensely greyish. Femora obscure yellow with paler bases, tibiae and basal tarsomeres brownish yellow, distal tarsomeres brown to dark brown. Male femur I: 3.6–3.8 mm long, II: 3.2 mm, tibia I: 3.2–3.7 mm, II: 3.3 mm, tarsus I: 4.8–5.3 mm, II: 4.9 mm. Claw small, dark brown, simple, without spines.

***Abdomen*.** Brown in male, yellowish in female, covered with sparse greyish pruinosity and short sparse pale setae. Posterior margins of segments narrowly pale greyish yellow in female, pale grey in male. Male terminalia (Fig. 70) obscure yellow. Posterior margin of epandrium with U-shaped concavity at middle, lateral lobe rounded, densely setose. Gonocoxite short and wide, length just slightly exceeds width, apex dorsally produced into large rounded lobe covered with small blackish spinules. Interbase extended into slightly arched blade. Outer gonostylus elongate, blunt-apexed, fleshy, covered with short blackish spinules. Inner gonostylus pale and narrow, slightly curved at middle, mesal surface bearing few spine-shaped setae distally, which are especially distinct at apex. Aedeagus short with slightly widened apex, paramere elongate. Ovipositor (Fig. 71) yellow. Base of cercus and hypogynial valve slightly infuscate, brownish, tips pale. Cercus arched, apex blunt and raised upwards. Hypogynial valve nearly straight, parallel-sided, blunt-apexed. Dorsal margin with few short setae at ~ 1/3 of length.

#### Elevation range.

From 1500 m to nearly 1900 m.

#### Period of activity.

Adults fly briefly, from late July only to the beginning of August in Korea, but they are active in mid-September in southern Sakhalin (Savchenko and Krivolutskaya 1976).

#### Habitats.

Adults hide among high grass along stream margins in wet mixed forests (Savchenko and Krivolutskaya 1976).

#### General distribution.

North Korea, Sakhalin Island (Russia).

#### Remark.

The species is recorded from the Korean Peninsula for the first time.

### ﻿ Dicranota (Rhaphidolabis) yeongokia

Taxon classificationAnimaliaDipteraPediciidae

Podenas sp. nov.

4718333F-740A-5A66-9805-952302CF19E3

https://zoobank.org/4790DDD3-FF3E-414E-8EE8-2D91EEECC2E1

Figs 72–76, 93

#### Type material examined

**(Fig. 93). South Korea** • ***Holotype*** ♂ (in ethanol); Gangwon-do, Gangneung, Yeongok-myeon, Samsan-ri, Odaesan National Park; 37.81161°N, 128.70116°E; alt. 280 m; 2 May 2012 (2); S. Podenas leg.; net; NIBR. ***Paratypes*** • 27 ♂, 5 ♀ (in ethanol, wing of 1 ♂ slide mounted, genitalia of 1 ♂ in microvial with glycerol); Gangwon-do, Gangneung, Yeongok-myeon, Samsan-ri, Odaesan National Park; 37.81161°N, 128.70116°E; alt. 280 m; 2 May 2012 (2); S. Podenas leg.; net; NIBR.

#### Diagnosis.

Pale yellow species with contrastingly black eyes. Wing semi-translucent, milky without darker areas and without stigma. Male gonostylus with large mesal lobe, posterior margin of epandrium with U-shaped invagination, margins of which usually asymmetrical, interbase large with elongate postero-dorsal and postero-ventral angles, the latter with long curved spine. Female cercus just slightly arched with distinct black base of ventral margin.

#### Etymology.

The species is named after the locality where it was collected, Yeongok-myeon.

#### Description.

General body colouration pale yellow. Body length of male 5.0–5.7 mm, of female 7.3 mm. Wing length of male 5.0–6.2 mm, of female 5.9–6.4 mm.

***Head*.** Greyish yellow, paler posteriorly, covered with sparse short pale setae. Eyes widely separated, distance between them at base of antenna nearly equals length of both basal antennomeres. Antenna 0.9–1.0 mm long in male, reaching slightly beyond frontal margin of prescutum if bent backwards, 1.1 mm in female. Scape pale, subcylindrical, ~ 2 × as long as second antennomere, bearing few setae dorsally. Pedicel pear-shaped, slightly darker than scape. Flagellum 13–14-segmented, pale brown, basal flagellomeres oval, distal flagellomeres slightly elongate, apical flagellomere comparatively large, but shorter than preceding segment. Longest verticils nearly as long as respective flagellomeres. Rostrum and palpus yellow. Labellum pale.

**Figures 72–76.**
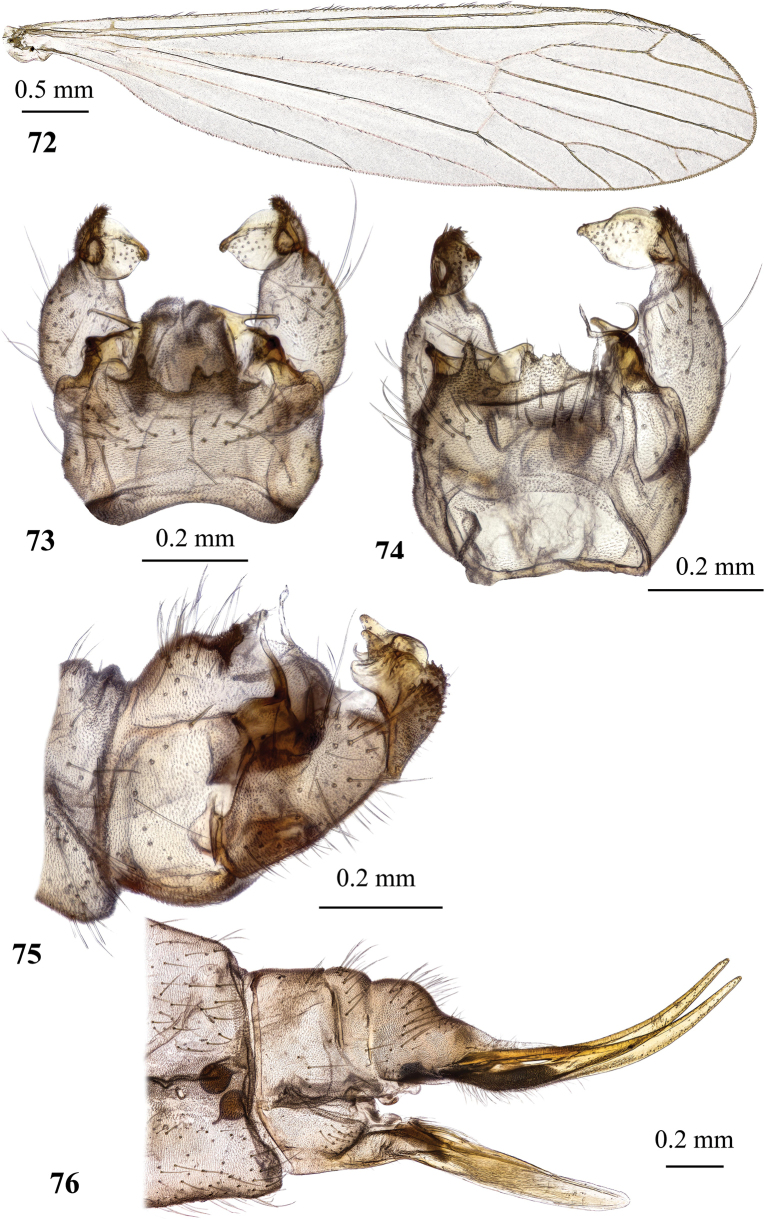
Dicranota (Rhaphidolabis) yeongokia Podenas, sp. nov., paratypes **72** wing **73** male genitalia, dorsal view **74** male genitalia, dorso-lateral view **75** male genitalia, lateral view **76** ovipositor, lateral view.

***Thorax*.** Pale brownish yellow. Cervical sclerites pale. Pronotum somewhat darker, bearing few erect setae dorsally. Presutural scutum uniformly pale brownish yellow, longitudinal stripes missing (stripes could be faded because of preservation in ethanol). Tubercular pit missing, pseudosutural fovea indistinct. Prothoracic spiracle surrounded by pale membrane. Scutal lobe concolourous with presutural scutum. Scutellum paler. Mediotergite pale brownish yellow. Pleuron paler than dorsum, darker areas missing. Wing (Fig. 72) elongate, length/width ratio 4.2, widest approximately at tip of vein *CuP*, semi-translucent, milky. Stigma and any darker areas missing. Veins greyish to brownish, paler at wing base. Venation: *Sc* long, reaching wing margin slightly beyond level of branching point of *R_2+3+4_*, *sc-r* slightly closer to humeral vein than to origin of radial sector. *Rs* short, 2.5 × as long as cross-vein *m-cu*, slightly arched. Free end of *R_1_* nearly missing, reaching wing margin together with *R_2_*. Vein *R_2_* oblique. *R_3_*, *R_4_*, and *R_5_* parallel to each other. Cell *r_3_* with distinct stem. Cross-vein *r-m* also distinct. Discal cell missing due to atrophy of vein *m-m*. Cell *m_1_* very short, its stem 4 × as long as cell itself. Cross-vein *m-cu* less than its own length beyond branching point of *M*. Vein *CuP* nearly straight, *A_1_* slightly sinuous. Anal angle long and narrow. Halter long, uniformly pale yellow. Length of male halter 0.8–0.9 mm, of female 0.8–0.9 mm. Coxae and trochanters pale yellow, distal margin of trochanter narrowly blackened. Femora pale brownish yellow. Tibiae pale yellow with slightly darkened apices. Basitarsi pale yellow at base, turning darker towards distal end, remaining tarsomeres brownish. Male femur I: 3.2 mm long, II: 3.8 mm, III: 3.6–4.0 mm, tibia I: 3.7 mm, II: 3.8 mm, III: 3.9–4.5 mm, tarsus II: 4.2 mm, III: 4.7–4.8 mm. Female femur I: 2.9 mm long, II: 3.4 mm, III: 3.5–3.7 mm, tibia I: 4.2 mm, II: 3.5 mm, III: 4.1 mm, tarsus I: 4.3 mm, II: 3.6 mm, III: 3.0–3.7 mm. Claw small and simple, nearly straight, without spines.

**Figures 77–93.**
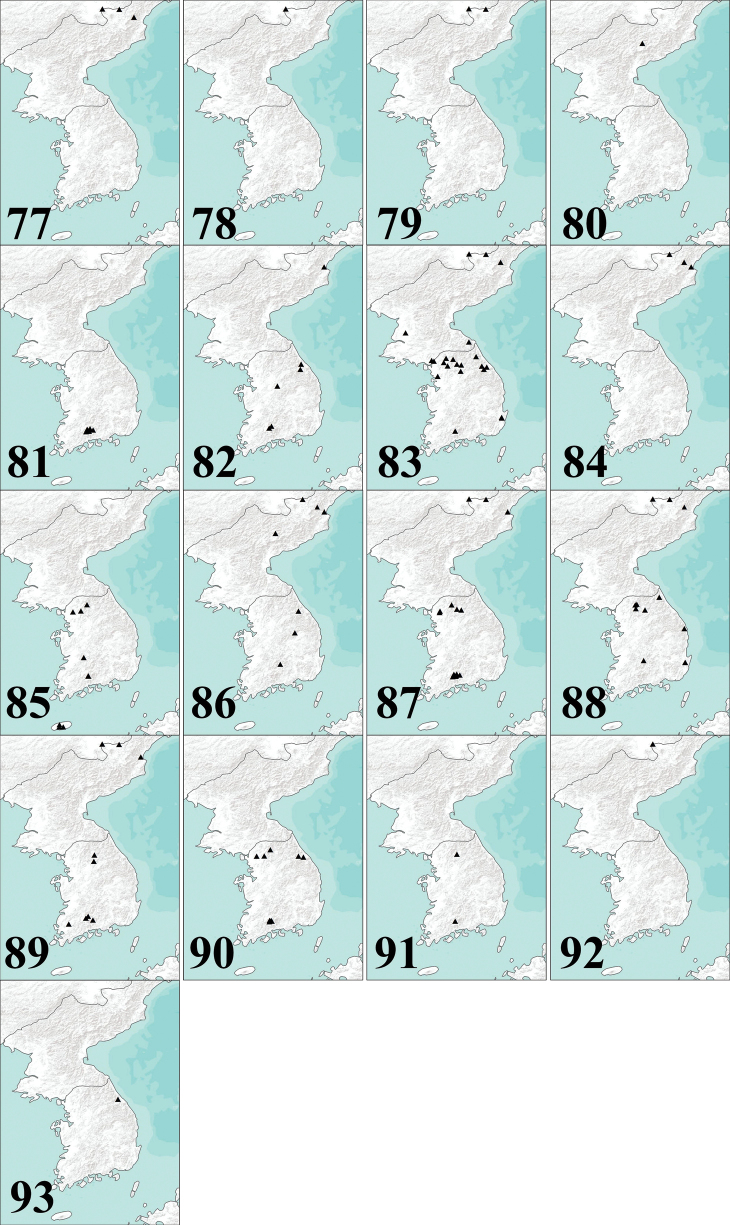
Sampling localities of Korean *Dicranota***77**D. (Dicranota) coreana Alexander, 1940, stat. nov.**78**D. (Dicranota) crassicauda Tjeder, 1972 **79**D. (Dicranota) guerini Zetterstedt, 1838 **80***D. (Dicranota) yezoensis* Alexander, 1924 **81**D. (Eudicranota) distincta Podenas, sp. nov. **82**D. (Eudicranota) perdistincta Alexander, 1940 **83**D. (Eudicranota) sibirica sibirica (Alexander, 1925) **84**D. (Ludicia) emarginata (Alexander, 1945) **85**D. (Rhaphidolabis) gibbera (Alexander, 1921) **86**D. (Rhaphidolabis) luteola Alexander, 1938 **87**D. (Rhaphidolabis) minuscula Alexander, 1938 **88**D. (Rhaphidolabis) neoconsors Alexander, 1938 **89**D. (Rhaphidolabis) ompoana Alexander, 1945 **90**D. (Rhaphidolabis) polymera Alexander, 1933 **91**D. (Rhaphidolabis) seoi Podenas, sp. nov. **92**D. (Rhaphidolabis) squarrosa Savchenko, 1976 **93**D. (Rhaphidolabis) yeongokia Podenas, sp. nov.

***Abdomen*.** Pale, pregenital segments slightly infuscate. Male terminalia (Figs 73–75) pale brownish yellow. Posterior margin of epandrium with U-shaped concavity at middle, bottom of which with few setae starting from small bumps. Setae often not symmetrically arranged on both sides. Margin of concavity extended into larger setose lobe, two or three much smaller lobules could be present further laterally. Often size of larger lobe is different on both sides, number of smaller lobules often varies too, and sometimes they are missing completely. Thus posterior margin of epandrium often, but not always, is asymmetrical. Gonocoxite slightly elongate, 1.4 × as long as wide, approximately egg-shaped, without additional spiny lobe distally, but with few short spine-shaped setae subapically. One pair of gonostyli. Outer part of gonostylus darkened, elongate, basal half of mesal surface strongly swollen, rounded, with darker margin, lateral part elongated, darker brown, covered with small spines on distal two-thirds and apex. Middle of mesal margin with large pale, subglobular lobe bearing small brownish rostral appendage, lower, expanded part with scattered sensilla. Interbase extended into large flattened lobe, postero-dorsal angle of which extended into slightly arched spine. Postero-ventral angle extended into long rostrum, bearing long curved spine, turned backwards. Aedeagus short, not visible in dorsal view, tip shallowly bifid. Paramere elongate, slightly curved. Posterior segments of female abdomen generally yellow, concolourous with the rest of abdomen. Tenth tergite yellow. Cercus (Fig. 76) yellow, slightly arched, tip raised upwards, blunt-apexed with paler distal part and distinctly blackened basal part of ventral margin, dorsal margin slightly infuscate. Hypogynial valve straight, obscure yellow with brownish base and pale distal part. Dorsal margin with few short subbasal setae, reaching to ~ 1/4 of valve. Two small subglobular spermathecae.

#### Habitat.

Slopes to the fast running mountainous stream with waterfalls covered with mixed forest, sparse herbaceous vegetation. Collected during small rain. Preimaginal stages unknown.

#### Elevation.

Near 300 m.

#### Period of activity.

Beginning of May.

#### Distribution.

Currently known only from Odaesan National Park, South Korea.

#### Remarks.

Dicranota (R.) yeongokia Podenas, sp. nov. generally similar to D. (R.) luteola, both are small yellow species, but distinct differences are observed in many structures: antenna of *D. yeongokia* Podenas, sp. nov. has 15–16 segments, that of *D. luteola* 13-segmented; wing cell *m_3_* with distinct stem, anal angle long and narrow in D. (R.) yeongokia Podenas, sp. nov., stem of cell *m_3_* very short or missing, anal angle wide in *D. luteola*; big differences are observed in male genitalia, especially in structure of epandrium and gonostylus. Despite crane flies are intensively collected for more than a decade in many different localities throughout South Korea, D. (R.) yeongokia Podenas, sp. nov. was observed only once in Odaesan National Park. Probably it has very short flying period and very limited distribution.

## Supplementary Material

XML Treatment for Dicranota

XML Treatment for Dicranota (Dicranota)

XML Treatment for Dicranota (Dicranota) coreana

XML Treatment for Dicranota (Dicranota) crassicauda

XML Treatment for Dicranota (Dicranota) guerini

XML Treatment for Dicranota (Dicranota) yezoensis

XML Treatment for Dicranota (Eudicranota)

XML Treatment for Dicranota (Eudicranota) distincta

XML Treatment for Dicranota (Eudicranota) perdistincta

XML Treatment for Dicranota (Eudicranota) sibiricasibirica

XML Treatment for Dicranota (Ludicia)

XML Treatment for Dicranota (Ludicia) emarginata

XML Treatment for Dicranota (Rhaphidolabis)

XML Treatment for Dicranota (Rhaphidolabis) gibbera

XML Treatment for Dicranota (Rhaphidolabis) luteola

XML Treatment for Dicranota (Rhaphidolabis) minuscula

XML Treatment for Dicranota (Rhaphidolabis) neoconsors

XML Treatment for Dicranota (Rhaphidolabis) ompoana

XML Treatment for Dicranota (Rhaphidolabis) polymera

XML Treatment for Dicranota (Rhaphidolabis) seoi

XML Treatment for Dicranota (Rhaphidolabis) squarrosa

XML Treatment for Dicranota (Rhaphidolabis) yeongokia

## ﻿Additional information

### Author ORCIDs

Sigitas Podenas https://orcid.org/0000-0002-2597-566X

Soen Yi Kim https://orcid.org/0009-0003-6967-3668

Jisoo Kim https://orcid.org/0000-0002-2570-1124

Virginija Podeniene https://orcid.org/0000-0002-3807-2347

### Data availability

All of the data that support the findings of this study are available in the main text.
